# Reinvestigating Pyrrol-2-One-Based Compounds: From Antimicrobial Agents to Promising Antitumor Candidates

**DOI:** 10.3390/ph18121813

**Published:** 2025-11-27

**Authors:** Natalia Simionescu, Ashraf Al-Matarneh, Ionel I. Mangalagiu, Narcis Cibotariu, Cristina Mariana Uritu, Cristina Maria Al-Matarneh, Mariana Pinteala

**Affiliations:** 1 Centre of Advanced Research in Bionanoconjugates and Biopolymers, “Petru Poni” Institute of Macrmoleclar Chemistry of Romanian Academy, 41A Grigore Ghica Voda Alley, 700487 Iasi, Romania; natalia.simionescu@icmpp.ro (N.S.);; 2 Faculty of Chemistry, Alexandru Ioan Cuza University of Iasi, 11 Carol I, 700506 Iasi, Romania; ionelm@uaic.ro; 3 Advanced Center for Research and Development in Experimental Medicine “Prof. Ostin C. Mungiu”, “Grigore T. Popa” University of Medicine and Pharmacy, 700115 Iasi, Romania

**Keywords:** pyrrol-2-one, antitumor activity, iodine, osteosarcoma

## Abstract

**Background:** Heteroaromatic iodine-containing compounds have been previously recognized for their broad-spectrum antimicrobial activity. This study aims to systematically investigate their potential repurposing as anticancer agents, with a particular focus on understanding the structural determinants that influence their cytotoxicity and selectivity toward malignant cells. **Methods:** A series of heteroaromatic iodine-containing derivatives were synthesized and evaluated for anticancer activity. Their cytotoxic effects were measured and compared between cancerous and normal cell lines to determine selectivity. Structural features, including heteroaromatic moieties and substituents, were analyzed to identify correlations with biological activity. **Results:** Among the tested compounds, derivatives 3e, 3g, and 3l demonstrated significant cytotoxic effects while exhibiting favorable selectivity indices. These findings indicate that these compounds preferentially target malignant cells over normal cells, thereby mitigating the issue of systemic toxicity often associated with traditional chemotherapeutics. The enhanced anticancer activity appears to be influenced by specific structural elements within the heteroaromatic framework. **Conclusions:** The study highlights the potential of heteroaromatic iodine-containing compounds as promising anticancer candidates. Rational structural modifications within these heterocyclic systems can effectively modulate bioactivity and improve therapeutic selectivity. These results support further development of this compound class for anticancer applications.

## 1. Introduction

Cancer remains one of the leading causes of mortality worldwide, accounting for approximately one in every six deaths and affecting millions of families each year [1]. In 2022 alone, around 20 million people were newly diagnosed with cancer, while 9.7 million lost their lives to the disease. With global incidence projected to rise by nearly 77% by 2050, the impact on healthcare systems, economies, and communities will become increasingly severe [2].

Beyond its devastating health burden, cancer often strikes during an individual’s most productive years, leading to workforce attrition, escalating healthcare costs, and setbacks in achieving sustainable development goals [3]. The most prevalent cancers include breast, lung, colorectal, and prostate cancers, of which lung cancer is the leading cause of cancer-related deaths, while breast cancer is the most frequently diagnosed type worldwide [4].

While these common malignancies dominate the global landscape, less prevalent but highly aggressive tumors, such as osteosarcoma, present unique therapeutic challenges [5]. Osteosarcoma, the most frequent primary bone cancer, predominantly affects children and young adults [6]. Despite advances in surgery and chemotherapy, survival rates for metastatic or recurrent osteosarcoma remain dismally low, underscoring the urgent need for innovative therapeutic strategies [7]. As with many cancers, drug resistance and systemic toxicity limit the long-term success of current treatments, reinforcing the importance of developing safer and more effective anticancer agents [5].

One promising area of research lies in the design of heteroaromatic compounds, particularly nitrogen-containing heterocycles, which are foundational in modern drug discovery [8,9]. Indeed, nearly 59% of FDA-approved small-molecule drugs contain at least one heterocycle, and nitrogen is present in 85% of these compounds [10]. Nitrogen atoms enhance lipophilicity, hydrogen bonding, and molecular stability, improving pharmacokinetic and pharmacodynamic profiles [8,11]. Classes such as benzimidazoles, benzothiazoles, indoles, acridines, oxadiazoles, imidazoles, pyrazoles, triazoles, quinolines, and quinazolines exhibit diverse biological effects, including cytotoxic, apoptosis-inducing, and metastasis-inhibiting properties [12,13,14]. Pyrrole-based derivatives, in particular, have attracted significant attention due to their multifunctionality: beyond antitumor activity, they display anti-inflammatory, antibacterial, antioxidant, tyrosinase-inhibitory, and protein–protein interaction-disrupting properties [15,16]. This breadth of activity makes them adaptable scaffolds for multifunctional drug design, capable of targeting multiple cellular pathways simultaneously to overcome resistance and improve treatment outcomes. Among these, pyrrol-2-one derivatives have been widely studied for their antitumor potential and are frequently associated with kinase inhibition mechanisms, particularly against targets such as EGFR and VEGFR [15]. This behavior parallels that of well-known tyrosine kinase inhibitors like sunitinib, which contains a related indolin-2-one core structure. The structural similarity and biological relevance of these scaffolds underscore the continued interest in pyrrol-2-one systems as versatile frameworks for anticancer drug development.

In parallel, repurposing of existing chemical structures has emerged as a strategic approach to accelerate drug development [17]. By leveraging compounds with established pharmacological, physicochemical, or safety profiles, researchers can streamline therapeutic innovation while reducing costs, risks, and timelines [18,19]. Repurposing not only provides regulatory advantages—such as expedited approval pathways—but also fosters equitable access to therapies, particularly in resource-limited settings [20,21]. Importantly, repurposed molecules can reveal unanticipated applications: drugs initially designed as antimicrobials, imaging agents, or enzyme inhibitors may demonstrate anticancer properties when re-evaluated in new biological contexts [22,23].

Within this framework, iodine-substituted compounds exemplify the power of repurposing and chemical modification in cancer research. Initially developed as radiographic contrast agents (e.g., iohexol, iopamidol, iodixanol), iodinated molecules have been successfully adapted for nuclear medicine applications [24]. Radioiodine isotopes such as ^123^I, ^125^I, and ^131^I are widely used in imaging and targeted therapies, including the treatment of thyroid cancer and radioimmunotherapy for non-Hodgkin’s lymphoma [25]. Beyond radiolabeling, iodination imparts crucial physicochemical benefits: it alters lipophilicity, enhances membrane permeability, modulates metabolic stability, and strengthens molecular interactions with biological targets [26]. These modifications can significantly improve therapeutic efficacy, whether through direct anticancer action, photoablation and photothermal strategies using iodine-functionalized nanoparticles, or by enhancing the activity of existing scaffolds like heteroaromatics [27]. Moreover, iodine-containing compounds such as povidone-iodine and iodoacetamide demonstrate the element’s versatility, functioning as antimicrobials, protein-labeling reagents, and modulators of tumor biology [28,29].

Taken together, the integration of nitrogen-based heterocycles, chemical repurposing, and iodination strategies represents a powerful frontier in oncology drug discovery. By combining structural innovation with translational efficiency, it is possible to design multifunctional molecules with enhanced selectivity, reduced toxicity, and broader therapeutic potential. Such advances hold particular promise not only for prevalent malignancies like breast and lung cancer but also for aggressive, underserved tumors such as osteosarcoma—ultimately contributing to the development of next-generation anticancer agents capable of addressing critical gaps in global cancer care. Given the clinical need for cytotoxic agents, the current work encompasses a systematic investigation of the design, presumed mechanisms of action, and anticancer potential of heteroaromatic iodine-containing compounds previously characterized as broad-spectrum antimicrobials, with the goal of assessing their repurposing value.

## 2. Results and Discussions

### 2.1. Synthesis

**Figure 1:**
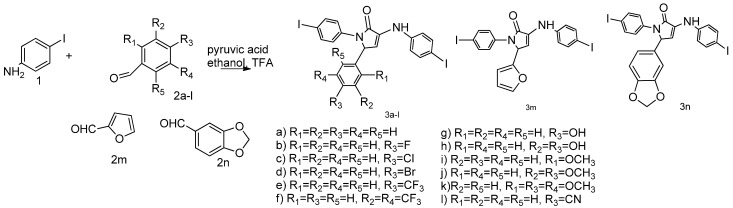
Reaction pathway to compounds **3a**–**n**.

Pyrrole derivatives have emerged as promising candidates in anticancer therapy [30] due to their ability to modulate key cellular targets such as microtubules, tyrosine kinases, cytochrome P450 enzymes, histone deacetylases, and Bcl-2 proteins, thereby enhancing the efficacy of existing treatments like tamoxifen [31]. We previously synthesized hybrid molecules that have two iodine atoms on their sides and a core constituted of a small and active heterocycle, pyrrolo-2-one, respectively [29]. Thus, we used iodoaniline (1), aromatic substituted benzaldehyde (**2a***–***n**), pyruvic acid and a catalytic amount of trifluoroacetic acid in ethanol media (Figure 1) to obtain the desired derivatives. Accordingly, with the withdrawing effect of the substituted aniline, we obtained 1H-pyrrol-2(5H)-one derivatives **3a**–**n**, and not carboxyquinolines, although the used conditions are well known as the Doebner reaction, a classic pathway for the construction of quinolines [32].

The compounds, previously investigated for their broad-spectrum antimicrobial activity, demonstrated significant efficacy against both Gram-positive and Gram-negative bacteria as well as yeasts. Notably, derivatives such as **3g** (*p*-OH), **3m** (furan-2-yl), and **3n** (benzodioxol-5-yl) showed the highest antibacterial activity, while **3l** (*p*-CN), and **3i** (*o*-OMe) were most effective against fungal strains [28]. These results underscored the influence of structural variations on microbial envelope interactions and highlighted the potential of certain compounds, particularly those active against both *C. albicans* and *S. aureus*, as leads for addressing polyspecific infections. Building on these promising findings, the current research redirects focus toward exploring the antitumor potential of these same compounds, aiming to leverage their bioactive scaffolds in targeting cancer cells.

### 2.2. In Silico Adsorption, Distribution, Metabolism, Excretion (ADME) and Toxicity Predictions

The first step was to evaluate the predicted parameters, including molecular properties, pharmacokinetics, drug-likeness, and medicinal chemistry, for compounds **3a**–**n**. The results of this analysis are summarized in Table 1.

Overall, the radar plots for these molecules would show shapes extending beyond the optimal region in multiple axes—most notably size, lipophilicity, and solubility—indicating that structural modifications are needed to improve their drug-likeness while retaining favorable polarity and flexibility.

The unified ADME summary table highlights clear trends in the theoretical physicochemical properties of compounds **3a**–**n**, revealing both strengths and limitations from a drug-likeness perspective. All molecules possess high molecular weights (568–714 g/mol), exceeding the Lipinski-recommended threshold of 500 Da, which can limit passive membrane permeability. LogP values range from 4.28 to 7.13, indicating predominantly lipophilic structures; while moderate lipophilicity supports membrane penetration, the upper values observed here risk poor aqueous solubility and increased metabolic liabilities. Indeed, most compounds are classified as poorly soluble, with only **3m** reaching moderate solubility, likely due to its heteroatom-containing ring systems. Despite their bulk, most compounds exhibit high predicted GI absorption and blood–brain barrier permeability, suggesting that low polarity (TPSA ~ 32 Å^2^ for most) and limited rotatable bonds favor passive diffusion. Notably, bioavailability scores are generally low (0.17), except for **3h**, **3k**, and **3m** (0.55), which combine more balanced lipophilicity with slightly increased polarity, offering a better physicochemical profile for oral delivery. This pattern underscores the need to reduce lipophilicity and molecular size while enhancing solubility to optimize these scaffolds for drug development.

Cancer cell line predictions indicated that the most prevalent targets among the studied compounds are colon, ovarian, lung, breast, urinary tract, and stomach cancers (Table S1–S14). Theoretical predictions from the CLC-Pred analysis further revealed that several compounds display promising probabilities of activity (Pa) against both osteosarcoma and glioblastoma cell lines, with corresponding Invariant Accuracy of Prediction (IAP) values above 0.80, suggesting strong model reliability. These findings are particularly significant because osteosarcoma and glioblastoma are high-mortality malignancies with limited effective treatment options and are not currently included as dedicated panels within the National Cancer Institute (NCI) standard screening program. By focusing on these underrepresented cancer types, our predictive modeling approach prioritizes compounds with potential translational relevance, directly addressing an unmet need in therapeutic development. This strategy not only fills a critical gap in current drug discovery pipelines but also aligns with precision oncology goals by directing research efforts toward cancers lacking standardized high-throughput screening models.

### 2.3. Anticancer Activity

**Figure 2:**
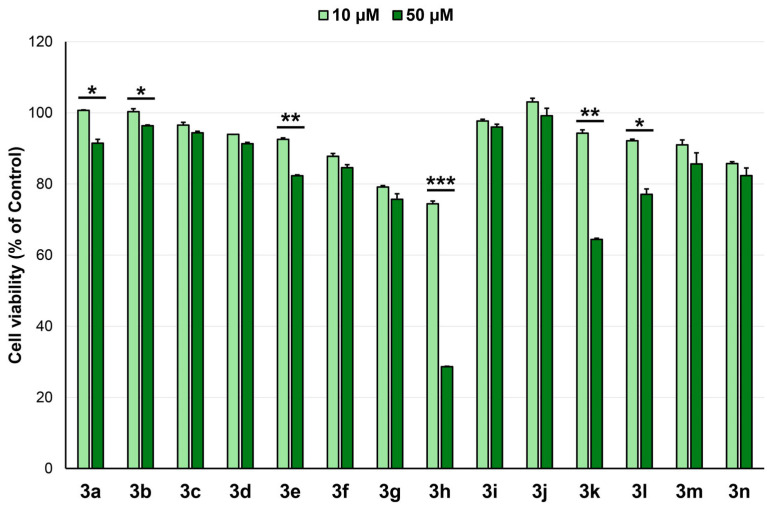
Cell viability of normal fibroblasts (HGF) incubated with compounds (10 and 50 µM) for 24 h, * *p* < 0.05, ** *p* < 0.01, *** *p* < 0.001 (10 µM vs. 50 µM, Student’s *t*-test).

The evaluation of anticancer potential, expressed as cytotoxicity, was performed in two steps: first on normal fibroblasts (HGF) and second on cancer cell lines. First, cytotoxicity tests performed on HGF cells (Figure 2) revealed that, at the tested concentration of 10 µM (10^–5^ M), all compounds are biocompatible (cell viability > 74%). At the tested concentration of 50 µM, compound **3h** presented acute cytotoxicity, while compound **3k** presented borderline cytotoxicity (64% cell viability).

In the next step, all synthesized compounds were electronically submitted and accepted for testing to the National Cancer Institute (NCI) platform, for the single dose (10^−5^ M) screening against a panel of 60 human tumor cell lines, representing leukemia, melanoma, and cancers of the lung, colon, central nervous system, ovary, kidney, prostate, and breast [33,34,35,36,37,38]. The results are summarized in Table 2, with the best values highlighted in red (Figures S1–S14).

Analysis of the data revealed that several compounds demonstrated moderate to strong inhibitory activity across multiple cancer cell lines, whereas others showed selective or weak effects. Compounds **3e**, **3h**, and **3l** emerged as the most active derivatives, achieving near-complete or complete inhibition (≥100% GI) in several tumor types. In contrast, compounds such as **3a**, **3b**, and **3c** exhibited relatively weak inhibition, with GI% values often below 30%, suggesting limited cytotoxic potential at the tested concentration.

Leukemia and breast cancer cell lines were among the most sensitive to the tested compounds. Notably, compound **3h** induced complete growth inhibition in multiple leukemia sublines, including CCRF-CEM, HL-60(TB), MOLT-4, RPMI-8226, and SR. Similarly, compound **3g** exhibited high potency against RPMI-8226, MOLT-4, and SR, while **3e** also showed strong activity across this panel. These findings suggest that some derivatives preferentially target rapidly proliferating hematological malignancies.

Several non-small cell lung cancer sublines also responded strongly to the tested compounds. Compounds **3e** and **3l** inhibited NCI-H226, and EKVX cells with GI values of 100%. In the colon cancer panel, **3h** was particularly active, showing significant growth inhibition (>80%). CNS cancer cell lines displayed variable sensitivity. **3b**, **3e**, **3f** and **3l** inhibited SNB-75 and SF-268 with GI% values ≥100%, suggesting strong potential against these tumor types. Among melanoma lines, **3e** and **3h** once again demonstrated superior potency, completely inhibiting the growth of SK-MEL-2, and MALME-3M. Also compounds **3l** and **3m** presented selectivity in SK-MEL-2 line. In ovarian cancer, **3e**, **3h** and **3l** were notably effective against OVCAR-4 with selectivity.

Renal cancer cell lines, including A498, SNC-12, TK-10 and ACHN, also showed strong inhibition by **3e**, **3h** and **3l**, whereas prostate cancer lines were generally less sensitive, with the exception of PC-3, which showed >70% inhibition under treatment with **3h**. In the breast cancer panel, the triple-negative subtype MDA-MB-231/ATCC and HS 578T cells were highly responsive to **3l** and **3e**, **3f**, **3g**, exhibiting GI values of 100%, while **3m** and **3h** inhibited MDA-MB-468.

When comparing across all derivatives, compound **3h** consistently demonstrated the strongest and broadest-spectrum growth inhibition, followed by **3e** and **3l**, which showed potent but somewhat more selective activity. In contrast, compounds such as **3m** and **3n** were less effective, with GI% values remaining below 40% in most tumor lines. The consistent high activity of **3e**, **3h**, and **3l** across multiple tumor types suggests that having only *para* position occupied of substituents such as -CF_3_, -OH or -CN may be critical for enhanced antiproliferative activity. Their broad activity, especially against leukemia, melanoma, and breast cancer cell lines, highlights their potential as lead scaffolds for further optimization. The reduced activity observed in prostate cancer lines (except PC-3) indicates possible tumor selectivity, which warrants further mechanistic studies.

In addition to broad-spectrum inhibition, several compounds displayed distinct tumor-type selectivity, suggesting possible mechanistic preferences or tumor-specific vulnerabilities. Compound **3f** showed preferential activity toward leukemia cell line CCRF-CEM, SNB-75 (CNS Cancer) and two lines in the breast cancer panel (HS 578T and T-47D). Compound 3b demonstrated a narrower profile, with moderate activity observed primarily in the CNS but with selectivity on the SNB-75 line. Compound **3m** exhibited selective inhibition in the melanoma cancer panel, with the SK-Mel-2 line and in breast cancer on MDA-MB-468, while compound **3a** displayed selectivity on RPMI-8226 from the Leukemia panel.

Collectively, these selective profiles suggest that, although less potent overall than the broad-spectrum agents, compounds **3a**, **3b**, **3f**, and **3m** may serve as useful scaffolds for developing targeted therapies. Their tumor-type restrictions may offer an advantage in reducing systemic toxicity while providing efficacy in specific cancer subtypes. Further mechanistic studies are warranted to elucidate the molecular basis of this selectivity.

Based on their performance in the single-dose screen, compounds **3e**, **3h**, and **3l** were selected for further evaluation against the full panel of 60 tumor cell lines in the five-dose assays. The results of this extended analysis are summarized in Table 3. The in vitro antiproliferative activity of compounds **3e**, **3h**, and **3l** was investigated against a panel of human cancer cell lines representing different tumor types, including leukemia, non-small cell lung cancer, colon cancer, CNS cancer, melanoma, ovarian cancer, renal cancer, breast cancer, and prostate cancer [34,35,36,37,38]. The cytotoxicity parameters were expressed as GI_50_ (concentration required to inhibit 50% of cell growth), TGI (concentration producing total growth inhibition), and LC_50_ (concentration required to kill 50% of cells) (Figures S15–S17).

Among the tested derivatives, compound **3e** emerged as the most potent and broad-spectrum inhibitor. It showed remarkable activity against CNS cancer cell lines, with GI_50_ values of 2.60 μM for SNB-75, indicating strong cytotoxic potential in this cancer subtype. Likewise, melanoma cell lines were highly sensitive to **3e**, with GI_50_ values of 2.00 μM (MALME-3M) and ovarian cancer 1.82 μM (OVCAR-4). Line A498 from renal Cancer panel was also sensitive to **3e** with a value of 1.95 μM, while for line HOP-92 from Non-small Cell Lung Cancer, the value is 3.15 μM. Interestingly, for the breast cancer panel, **3e** was highly efficient for two lines, HS-578T and t-47D with values of 3.45 and 2.74 μM, respectively. Notably, line HOP-92 from Non-small Cell Lung Cancer proved to be sensitive to all tested compounds, **3h** and **3l** demonstrating GI50 values of 4.08 and 1.46 μM, respectively. Compound **3l** showed good activity against CNS cancer cell lines, with GI_50_ values of 1.34 μM for SNB-75, and 2.50 and 2.10 μM for MDA-MB-231/ATCC and HS 578T, respectively. On the OVCAR-4 line from ovarian cancer panel compound **3l** presented excellent activity of 422 nM, the best activity observed across all tested lines.

**Figure 3:**
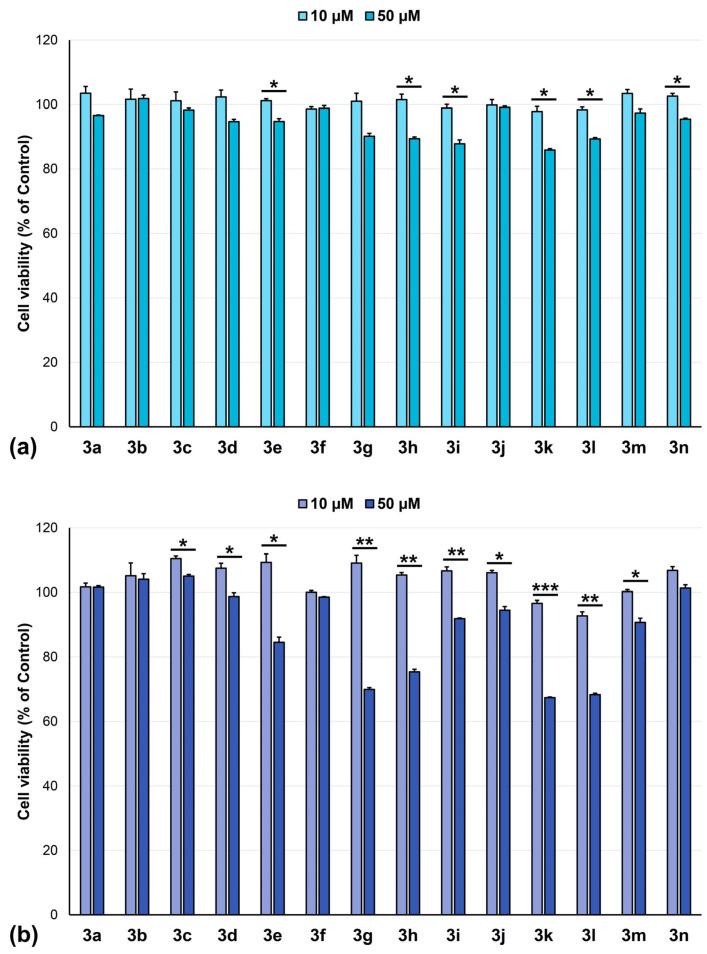
Cytotoxicity of compounds (10 and 50 µM) over 24 h incubation on glioblastoma cell lines: (**a**) LN-229, (**b**) U-118MG, * *p* < 0.05, ** *p* < 0.01, *** *p* < 0.001 (10 µM vs. 50 µM, Student’s *t*-test).

Based on favorable predictions and our research focus, the compounds were further evaluated for their activity against glioblastoma cell lines LN-229 and U-118MG (Figure 3). Tests on glioblastoma cell lines, consistent with CNS cancer results from NCI, showed that compounds **3e**, **3g**, **3h**, **3k**, and **3l** exhibited moderate activity against U-118MG cells at 50 µM, while being ineffective on LN-229 cell line. This could be explained by molecular differences between the two cell lines. According to the Cellosaurus database [39], LN229 is derived from the right frontal parieto-occipital cortex of a 60-year-old White female patient with glioblastoma, while U-118MG is derived from the astrocytoma of a 47-year-old Caucasian male patient. Although both cell lines exhibit adherent growth, their morphology is markedly different: LN-229 cells presenting epithelial-like morphology, while U-118MG mixed morphology. The presence of the mutated p53 gene in these cell lines confers aggressive behavior and resistance to apoptosis, but other molecular particularities could increase their susceptibility to specific drugs.

**Figure 4:**
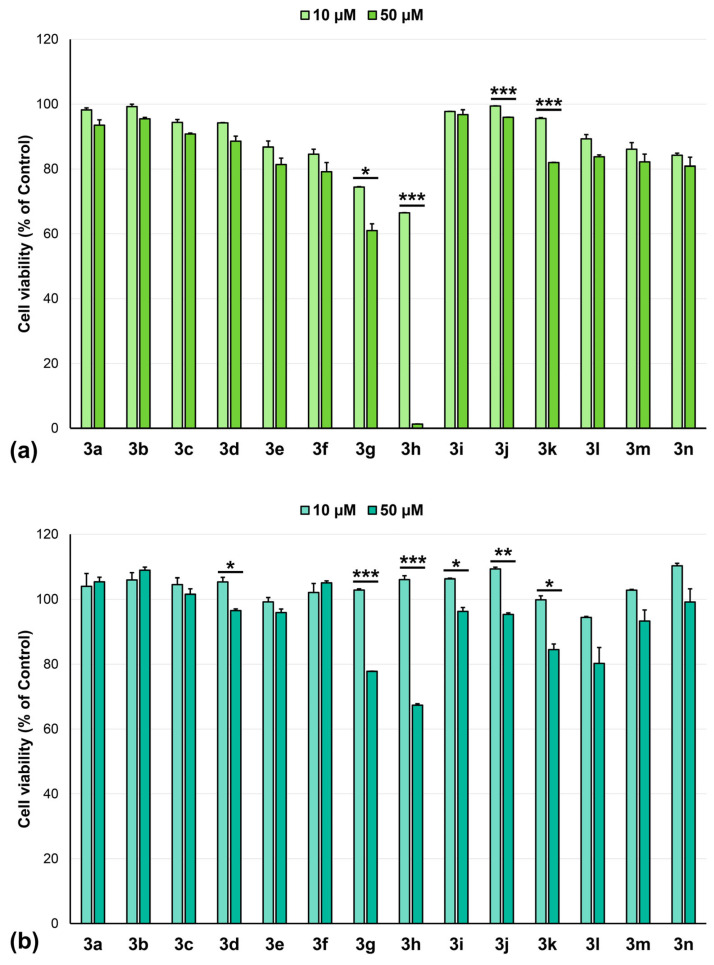
Cytotoxicity of compounds (10 and 50 µM) over 24 h incubation on osteosarcoma cell lines: (**a**) HOS, (**b**) MG-63, * *p* < 0.05, ** *p* < 0.01, *** *p* < 0.001 (10 µM vs. 50 µM, Student’s *t*-test).

Of particular interest were the tests on osteosarcoma cell lines, which are absent from the NCI panel. Compounds **3g** and **3h** showed notable activity, especially **3h** against HOS cells at 50 µM, while the other derivatives displayed only minor effects (Figure 4). As we have previously speculated [39], the susceptibility of different osteosarcoma cell lines, and implicitly of different tumor subtypes, to anticancer drugs may be due to differences in their molecular and genetic makeup, leading to specific therapeutic responses or resistance.

Comparing the anticancer activity of newly developed molecules with well-established drugs, such as sunitinib, is essential to contextualize their efficacy and guide optimization; in this study, the structural corroboration of the tested compounds with sunitinib, together with its experimentally validated anticancer results, served as a leading reference model for our evaluations [40,41].

### 2.4. Molecular Docking

Molecular docking performed on the VEGFR2 receptor aimed to compare the binding affinity of the reference inhibitor sunitinib with the best three candidates **3e**, **3h**, and **3l** (selection based on cytotoxicity assays).

During the blind docking phase, which explored the entire surface of the enzyme, the most energetically favorable binding poses of, as well as those of the selected ligands, were located within the same region identified as the binding site in the crystallographic structure. Therefore, subsequent local docking was focused on this region to refine the ligand-receptor interactions.

**Figure 5:**
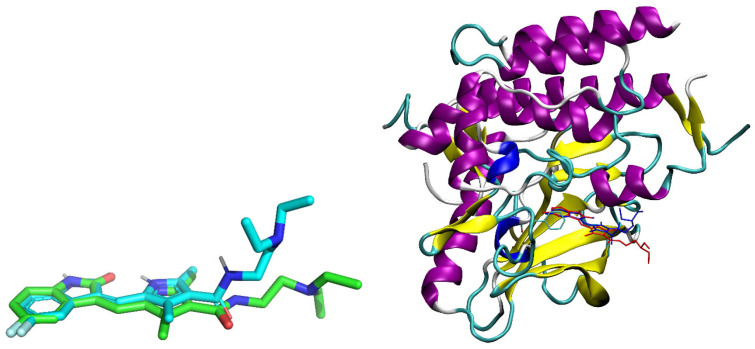
Superposition of the docked sunitinib pose (cyan) with the crystallographic structure (green).

The docking of sunitinib yielded a binding energy of −9.34 kcal/mol and provided strong evidence of the utility of the docking protocol since the predicted binding pose of sunitinib was found to closely match its experimentally orientation in the active site of VEGFR2. The only apparent difference lies in the flexibility of the ethylamine side chain which assumes a more bent shape in the docked form than it does in the experimentally determined form (Figure 5). However, this local flexibility is not an unrealistic expectation for the aliphatic chain of the sidechain and should not have affected the docked orientation of the pharmacophore portion of the compound. There is also a π–π stacking interaction between the fused benzene ring of the fluorophenyl fragment in the indolizine core and PHE1047, at a distance of 5.38 Å, very close to that observed experimentally (5.40 Å). Two hydrogen bonds were identified: one with GLU917 and the other with CYS919, contributing to anchoring the indolizine moiety. The hydrophobic environment of the ligand is represented by VAL848, LEU840, VAL916, PHE921, ALA866, and LEU1035, CYS1045, PHE1047 which stabilize the complex through van der Waals (vdW) interactions, while the hydrophilic region (ASN923, THR926, SER930) contributes to the polar orientation of the ligand in the cavity. Notably, the docking of sunitinib produced only two large clusters, showing great convergence of solutions and a stable, well-defined binding mode.

Compound **3e** binds with an energy of −9.13 kcal/mol and retains the π–π stacking interaction with PHE1047, as seen for sunitinib. The hydrophobic environment of the ligand consists of LEU840, ALA866, VAL899, VAL916, PHE918, CYS919 and PHE1047, residues that may contribute to the stabilization of the complex in the protein cavity through vdW interactions.

Compound **3h** is energetically comparable to the binding energy of sunitinib (−9.44 kcal/mol) and has a broader interaction network, comprising a hydrogen interaction with the ASN923 and two other bonds with the hydroxyl groups and the LYS838 and PRO839 side-chains. These multiple polar contacts increase the interaction in the hydrophilic region and contribute to the stability of the complex. The exposure is hydrophobic and comprises VAL848, LEU840, PHE1047, VAL916, and ALA866, CYS919, PHE921.

In the case of compound **3l**, docking revealed the most favorable binding energy in the series studied (−9.58 kcal/mol), associated with two hydrogen bonds with ASN923. At the same time, the ligand is well anchored in a hydrophobic environment composed of LEU840, VAL848, CYS919, PHE921, GLY922, ASN923, THR926, LEU1035, and PHE1047, which stabilizes the structure of the complex.

**Figure 6:**
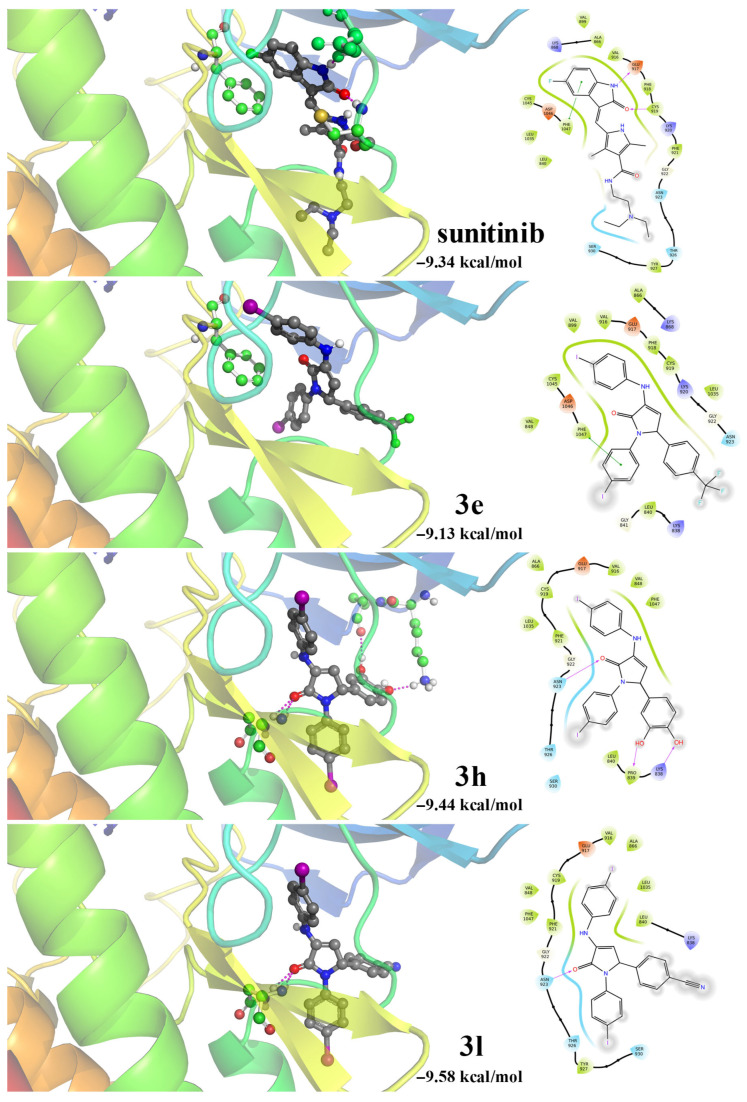
Binding modes of sunitinib and compounds **3e**, **3h**, and **3l**. left-3D views of each ligand within the binding pocket. Ligands are shown in CPK colors, and residues involved in hydrogen bonding or π–π stacking interactions are shown in ball-and-stick representation. Right-2D interaction diagrams illustrating ligand–protein contacts within a 4 Å cutoff. Protein residues are represented as droplet-shaped symbols whose orientation indicates whether the backbone (tip facing away) or side chain (tip facing the ligand) participates in the interaction. The residues are highlighted in color according to physicochemical properties: positive (blue), negative (red-orange), polar (cyan), and hydrophobic (green). Hydrogen bonds are shown in violet arrows, while the regions in gray are solvent-exposed.

Comparative analysis of the complexes (Figure 6) shows that all compounds retain the essential interactions in the pyrrolic core region or stacking with PHE1047, but differ in how they balance hydrophobic and hydrophilic components.

## 3. Materials and Methods

### 3.1. In Silico ADME and Toxicity Predictions

The ADME in silico evaluation for the compounds **3a**–**n** was performed using the SwissADME web tool (http://swissadme.ch/index.php accesed on 25 July 2025) in terms of molecular properties, pharmacokinetics, drug-likeness, and medicinal chemistry.

The in silico toxicological evaluation for the active compounds **3a**–**n** was performed using the web service Cell-Line Cytotoxicity Predictor (https://www.way2drug.com/clc-pred/ accesed on 26 July 2025), which screens for in silico cytotoxicity on a panel of 278 tumor cells and 27 normal human cell lines from different tissues.

### 3.2. In Vitro Cytotoxicity Assessment

Cytotoxicity of compounds was assessed using the CellTiter-Glo^®^ 2.0 Assay (Promega, Madison, WI, USA) on human gingival fibroblasts (HGF), osteosarcoma (HOS and MG-63) and glioblastoma (LN-229 and U-118MG) cells, according to the manufacturer’s instructions. HGF, HOS, MG-63 and U-118MG cells were purchased from Cytion GmbH, Eppelheim, Germany, and LN-229 (CRL-2611) from the American Type Culture Collection (ATCC), Manassas, VA, USA. Cells were seeded (50,000 cells/mL—HGF, 100,000 cells/mL—tumor cells) into 96-well opaque white tissue culture-treated plates and allowed to adhere overnight in αMEM medium with 10% fetal bovine serum and 1% antibiotic-antimycotic (all from PAN—Biotech GmbH, Aidenbach, Germany). Cells were incubated with compounds (10 or 50 µM) for 24 h, then CellTiter-Glo^®^ reagent was added and luminescence was recorded using a FLUOstar^®^ Omega microplate reader (BMG LABTECH, Ortenberg, Germany). The experiments were carried out in triplicate, and the viability of treated cells was expressed as a percentage of the viability of control cells (untreated). Data were represented as means ± standard deviations. Stock solutions of compounds were prepared in DMSO, then diluted in complete cell culture media. The final concentration of DMSO in cell culture media was lower than 0.5% (which does not affect cell viability). Data were analyzed with independent two-tailed (Student’s) *t*-test, considering *p* < 0.05 statistically significant.

### 3.3. Molecular Docking

The atomic coordinates of the VEGFR-2 receptor kinase domain were obtained from the Protein Data Bank (RCSB PDB) (PDB ID: 4AGD [42], which contains the enzyme in complex with the inhibitor sunitinib. The co-crystalized inhibitor and water molecules were removed using AutoDockTools 1.5.7, and polar hydrogen atoms were added, as well as Kollman charges were assigned to the protein, which was considered rigid in all simulations.

Due to structural similarity, sunitinib was chosen as the reference ligand.

The reference ligand (sunitinib) and the compounds of interest (**3e**, **3h**, and **3l**) were constructed in GaussView 5.0.9, and their geometry was optimized at the PM6 semiempirical level using Gaussian16 [43]. After obtaining the minimum energy geometry, Gasteiger partial charges were assigned to each ligand, and the corresponding rotational bonds were defined.

To identify favorable binding regions on the protein surface, blind docking was initially performed using an extended searching box that covered its entire surface. Simulations were performed with AutoDock 4.2 Release 4.2.6 [44] using the Lamarckian genetic algorithm [45]. For each ligand, nine independent simulations were performed, with 2000 genetic rounds, a population of 300 individuals, and a maximum number of 2.5 × 10^6^ energy evaluations. The resulting conformations were clustered hierarchically using a RMSD tolerance of 2.0 Å, which allows the identification of the most stable conformations based on the minimum energy and frequency of occurrence within the clusters. The blind docking results showed that, in their most favorable conformation, all compounds tend to bind to the same binding site as sunitinib, located in the catalytic cavity of the protein. Based on these observations, for the local docking stage, a new search box was defined around the active site identified in the crystallographic complex, centered at coordinates 50.07, −2.07, −14.87. Grid maps were generated using AutoGrid 4.2.6 with a spacing of 0.375 Å and effective grid dimensions of 44 × 44 × 44 points. The genetic parameters were kept identical to those in the initial simulation, except for the number of genetic rounds, which was reduced to 1000 for local refinement of the complexes. The 3D representations of the protein-ligand complexes were generated using PyMOL, while two-dimensional interaction diagrams were created using Maestro 2025-2.

## 4. Conclusions

We present herein a methodical study concerning the design, mechanism of action and anticancer activity of a series of heteroaromatic iodine-containing compounds.

Among the tested derivatives, compounds **3e**, **3h**, **3l**, and **3m** exhibited notable anticancer activity while demonstrating favorable selectivity indices, indicating a preferential effect toward malignant cells compared to normal cells. This selectivity is of particular importance, as it suggests a reduced likelihood of systemic toxicity, which remains a major limitation of conventional chemotherapeutics. The selective activity observed in these compounds can be attributed to the presence of specific heteroaromatic moieties and substituents that may enhance interactions with cancer-specific molecular targets.

The findings reinforce the concept that rational structural modifications within heterocyclic frameworks can significantly modulate biological activity and improve therapeutic windows.

Overall, these results highlight **3e**, **3h** and **3l** as promising lead candidates for further optimization and preclinical evaluation. A special remark should be addressed to compound **3l**, which possesses an excellent selective activity against the ovarian cancer cell line OVCAR-4, in the range of nanomolar concentration. Future work should focus on mechanistic studies to elucidate their precise molecular targets, as well as in vivo assessments to validate their efficacy and safety profiles. The molecular docking study suggests that all three compounds analyzed (**3e**, **3h**, and **3l**) interact favorably in the active site of VEGFR2, showing similarities to the reference inhibitor sunitinib, but also distinct interaction patterns. They share common features such as π–π stacking with residue PHE1047 and form hydrogen bonds with key residues in the active site, contributing to the stabilization of the ligand-receptor complexes. The differences between the compounds are reflected in the number and distribution of hydrophobic and hydrophilic contacts, which influence their overall binding profiles. Such investigations may pave the way for the development of novel, selective, and multifunctional anticancer agents based on heteroaromatic scaffolds.

## Figures and Tables

**Table 1:** In silico prediction of ADME parameters for compounds **3a**–**n** (selection).

Cmp	MW (g/mol)	LogP	Solubility	GI Absorption	BBB Permeant	Log *K*_p_ (Skin Permeation (cm/s)	Bioavailability	Synthetic Accessibility	Radar
**3a**	578.18	5.06	Poorly soluble	High	Yes	−5.47	0.17	3.68	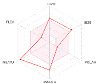
**3b**	596.17	5.41				−5.51	0.17	3.70	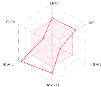
**3c**	612.63	5.63				−5.24	0.17	3.70	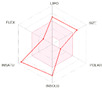
**3d**	657.08	5.72				−5.47	0.17	3.72	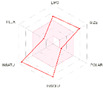
**3e**	646.18	6.12		Low	No	−5.26	0.17	3.81	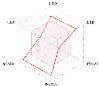
**3f**	714.18	7.13	Insoluble			−5.05	0.17	4.00	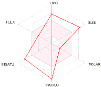
**3g**	594.18	4.70	Poorly soluble	High	Yes	−5.82	0.17	3.66	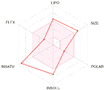
**3h**	610.18	4.28				−6.17	0.55	3.71	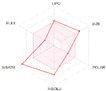
**3i**	608.21	5.10				−5.68	0.17	3.81	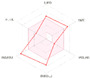
**3j**	638.24	5.05				−5.88	0.17	3.92	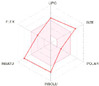
**3k**	668.26	4.98				−6.08	0.55	4.18	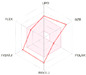
**3l**	603.19	4.87				−5.83	0.17	3.75	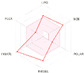
**3m**	568.15	4.37				−6.05	0.55	3.89	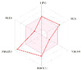
**3n**	622.19	4.86				−5.88	0.17	3.84	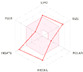

BBB—Blood–brain barrier, GI—gastrointestinal Absorption.

**Table 2:** Selected results of the in vitro growth inhibition (GI%) of tested compounds against human cancer cell lines in the single-dose assay ^a^.

Cell Type	Compound Cell Line	GI (%) (10^–5^ M) *													
		3a	3b	3c	3d	3e	3f	3g	3h	3i	3j	3k	3l	3m	3n
Leukemia	CCRF-CEM	4	74	15	17	100 ^c^	100 ^p^	94	100 ^w^	0	0	0	74	59	3
	HL-60(TB)	0	35	0	0	51	59	75	100 ^x^	0	0	2	0	30	16
	K-562	0	5	10	15	16	26	78	89	9	0	0	14	14	6
	MOLT-4	2	66	29	20	75	89	100 ^t^	100 ^y^	17	0	0	78	70	20
	RPMI-8226	100 ^a^	35	30	34	60	43	100 ^u^	100 ^z^	32	7	14	83	93	20
	SR	44	92	41	0	99	47	100 ^d^	100 ^a1^	23	0	7	87	76	27
Non-small Cell Lung Cancer	A549/ATCC	5	13	4	0	30	16	22	73	6	5	3	31	16	15
	EKVX	37	24	37	36	100 ^d^	35	64	22	49	40	0	99	39	12
	HOP-62	-	-	-	-	-	-	5	82	0	0	0	-	50	0
	HOP-92	0	36	27	0	62	19	66	66	20	0	0	75	29	4
	NCI-H226	4	4	62	17.	100 ^e^	81	47	35	36	^0^	0	100 ^i1^	22	3
	NCI-H23	6	8	13	3	11	12	45	73	30	8	0	36	19	18
	NCI-H322M	17	10	0	0	97	10	20	34	0	0	4	51	19	3
	NCI-H460	1	17	7	0	43	35	69	98	9	0	0	40	0	3
	NCI-H522	50	26	24	2	53	34	70	82	37	0	2	32	31	26
Colon Cancer	COLO205	0	27	0	0	61	59	42	98	0	9	0	46	8	5
	HCC-2998	0	0	0	0	0	2	53	98	21	0	5	50	10	6
	HCT-116	14	21	25	19	58	43	61	96	3	0	4	48	20	9
	HCT-15	19	9	13	13	12	17	84	84	13	0	0	21	16	9
	HT-29	0	16	19	14	32	5	96	96	5	0	0	40	6	5
	KM12	8	26	17	17	35	13	80	100 ^b1^	9	0	2	52	48	19
	SW-620	0	24	12	16	47	20	56	100 ^c1^	14	0	0	50	16	9
CNS Cancer	SF-268	16	68	24	15	100 ^f^	21	35	82	24	3	1	93	79	25
	SF-295							48	56	28	0	2		63	9
	SF-539	12	51	11	0	37	17	69	72	31	28	18	35	63	87
	SNB-19	8	8	7	6	71	19	17	69	0	0	0	35	8	5
	SNB-75	15	100 ^b^	12	3	100 ^g^	100 ^q^	77	86	37	0	0	100 ^j1^	9	20
	U251							29	58	0	0	0		0	0
Melanoma	LOX IMVI	18	0	11	1	7	17	44	57	2	8	5	89	63	10
	MALME-3M	0	6	17	0	78	0	83	100 ^d1^	45	0	0	46	34	0
	M14	0	4	0	0	64	0	52	99	10	0	0	63	4	5
	MDA-MB-435	0	0	0	0	0	0	38	73	0	0	0	19	84	0
	SK-MEL-2	26	31	43	16	100 ^h^	35	56	100 ^e1^	44	0	0	100 ^k1^	100 ^p1^	56
	SK-MEL-28	2	8	12	12	31	9	42	75	12	0	0	29	20	3
	SK-MEL-5	1	5	18	20	0	12	27	94	4	11	12	24	17	1
	UACC-257	13	8	23	3	100 ^i^	0	42	68	13	0	0	21	32	0
	UACC-62	0	0	5	0	12	0	33	62	21	0	0	17	61	0
Ovarian Cancer	IGROV1	16	7	16	16	12	0	27	26	57	0	2	54	8	5
	OVCAR-3	1	9	9	5	72	35	59	96	46	0	0	60	8	10
	OVCAR-4	20	38	34	30	100 ^j^	69	85	100 ^f1^	60	0	3	100 ^l1^	0	14
	OVCAR-5	0	0	0	0	18	9	24	22	0	0	0	11	0	0
	OVCAR-8	15	8	11	0	43	26	31	37	17	0	0	30	11	0
	NCI/ADR-RES	0	9	1	0	35	7	44	35	3	0	0	37	15	0
	SK-OV-3	8	12	13	0	50	10	25	64	19	0	0	75	67	13
Renal cancer	786-0	17	27	18	6	76	29		52		0	3	55	36	9
	A498	16	21	19	5	100 ^k^	90	48	61	6	0	0	88	12	16
	ACHN	13	35	21	15	100 ^l^	49		61		0	0	100 ^m1^	9	0
	CAKI-1	3	13	19	12	91	32	41	44	28	5	15	62	53	16
	SN12C	23	28	25	15	60	61	60	100 ^g1^	20	0	2	59	27	16
	TK-10	12	27	1	0	100 ^m^	57	43	50	30	0	1	99	7	0
	UO-31							54	54	27	0	0		17	6
Prostate cancer	PC-3	0	0	1	0	26	25	73	71	15	0	0	45	28	1
	DU-145	0	9	10	0	28	5	58	40	13	0	0	37	0	0
Breast cancer	MCF7	44	56	47	60	44	46	83	91	50	6	23	52	93	30
	MDA-MB-231/ATCC	0	6	5	0	62	42	44	42	25	0	0	100 ^n1^	11	0
	HS 578T	71	90	55	41	100 ^n^	100 ^r^	100 ^v^	63	84	0	0	100 ^o1^	39	25
	BT-549	-	-	-	-	-	-	54	81	2	0	10	-	17	3
	T-47D	34	32	48	49	100 ^o^	100 ^s^	80	65	39	6	8	59	49	22
	MDA-MB-468	-	-	-	-	-	-	96	100 ^h1^	43	22	44	-	100 ^q1^	83

* Data obtained from NCI’s in vitro 60 cell one dose screening at 10^–5^ M; Cytotoxic effect; Cell growth percent: ^a^ = −40, ^b^ = −2.5, ^c^ = −2.8, ^d^ = −7.5, ^e^ = −9.5, ^f^ = −8.36, ^g^ = −58.51, ^h^= −13.59, ^i^ = −5.48, ^j^ = −22.37, ^k^ = −84.15, ^l^ = −52.45, ^m^= −38, ^n^ = −68.58, ^o^ = −38.45, ^p^ = −7.36, ^q^ = −18.57, ^r^ = −34.84, ^s^ = −46.59, ^t^ = −77.31, ^u^ = −1.36, ^v^ = −0.46, ^w^ = −37.61, ^x^ = −96.03, ^y^ = −97.63, ^z^ = −81.29, ^a1^ = −94.10, ^b1^ = −24.54, ^c1^ = −37.34, ^d1^ = −11.84, ^e1^ = −2.44, ^f1^ = −3.18, ^g1^ = −10.67, ^h1^ = −9.99, ^i1^ = −19.81, ^j1^ = −51.73, ^k1^ = −2.74, ^l1^ = −89.48, ^m1^ = −9.55, ^n1^ = −11.56, ^o1^ = −20.41, ^p1^ = −40.23, ^q1^ = −6.43.

**Table 3:** Selected results of the 5-dose in vitro human cancer cell growth inhibition for compounds **3e**, **3h** and **3l**.

Cell Type	Compound Cell Line ↓	3e			3h			3l		
		GI_50_ (μM)	TGI (μM)	LC_50_ (μM)	GI_50_ (μM)	TGI (μM)	LC_50_ (μM)	GI_50_ (μM)	TGI (μM)	LC_50_ (μM)
Leukemia	HL-60(TB)	4.48	>100	>100	2.19	4.28	8.38	>100	>100	>100
	SR	2.84	>100	>100	1.55	2.99	5.75	6.28	>100	>100
	CCRF-CEM	3.35	>100	>100	3.09	>100	3.61	3.26	>100	>100
	MOLT-4	2.92	>100	>100	2.08	4.17	8.35	2.11	>100	>100
	RPMI-8226	5.38	>100	>100	2.40	6.01	>100	3.07	>100	>100
Non-small Cell Lung Cancer	NCI-H226	8.48	>100	>100	>100	>100	>100	4.42	>100	>100
	HOP-92	3.15	7.22	>100	4.08	8.97	>100	1.46	4.89	>100
	A549/ATCC	>100	>100	>100	>100	>100	>100	8.08	>100	>100
	HOP-62	>100	>100	>100	>100	>100	>100	5.21	>100	>100
Colon Cancer	HT29	>100	>100	>100	8.80	>100	>100	9.43	>100	>100
	SW-620	>100	>100	>100	>100	>100	>100	5.91	>100	>100
	KM12	>100	>100	>100	5.22	>100	>100	9.70	>100	>100
	HCT-116	5.92	>100	>100	7.43	>100	>100	3.83	>100	>100
	COLO 205	4.57	>100	>100	6.65	>100	>100	5.29	>100	>100
CNS Cancer	SF-268	4.66	>100	>100	6.04	>100	>100	4.69	>100	>100
	SNB-75	2.60	5.06	9.85	>100	>100	>100	1.34	3.42	8.76
	U251	8.97	>100	>100	>100	>100	>100	4.17	>100	>100
Melanoma	MALME-3M	2.00	4.32	9.34	2.68	>100	>100	2.07	>100	>100
	M14	8.94	>100	>100	5.34	>100	>100	7.24	>100	>100
	SK-MEL-2	>100	>100	>100	6.64	>100	>100	3.70	>100	>100
	LOX IMVI	>100	>100	>100	>100	>100	>100	3.38	>100	>100
Ovarian Cancer	OVCAR-3	7.48	>100	>100	9.16	>100	>100	4.72	>100	>100
	OVCAR-4	1.82	4.41	>100	3.27	>100	>100	0.422	1.69	4.82
	IGROV1	>100	>100	>100	>100	>100	>100	8.67	>100	>100
	SK-OV-3	>100	>100	>100	7.60	>100	>100	6.41	>100	>100
Renal Cancer	CAKI-1	3.72	>100	>100	>100	>100	>100	9.48	>100	>100
	A498	1.95	3.56	6.50	>100	>100	>100	2.99	>100	>100
	ACHN	4.05	>100	>100	>100	>100	>100	5.36	>100	>100
	RXF 393	4.23	>100	>100	6.25	>100	>100	3.72	>100	>100
	TK-10	>100	>100	>100	>100	>100	>100	3.85	>100	>100
Breast cancer	MDA-MB-231/ATCC	5.36	>100	>100	>100	>100	>100	2.50	9.63	>100
	HS 578T	3.45	9.97	>100	6.65	>100	>100	2.10	5.64	>100
	BT-549	9.61	>100	>100	>100	>100	>100	5.81	>100	>100
	T-47D	2.74	7.57	>100	2.41	>100	>100	7.13	>100	>100
	MDA-MB-468	3.24	>100	>100	3.10	>100	>100	2.70	>100	>100
Prostate cancer	PC-3	9.99	>100	>100	8.83	>100	>100	2.40	>100	>100

Data obtained from NCI’s in vitro 60 cell 5-dose screen. GI_50_—the molar concentration of the tested compound causing 50% growth inhibition of tumor cells. TGI—the molar concentration of the tested compound causing total growth inhibition of tumor cells. The best values shown by compounds are highlighted in red, god results are in blue, best result in green.

## Data Availability

The original contributions presented in this study are included in the article/supplementary material. Further inquiries can be directed to the corresponding author.

## Supplementary Materials

The following supporting information can be downloaded at: https://www.mdpi.com/article/10.3390/ph18121813/s1, Figure S1: Anticancer activity (single-dose (10^−5^ M) assay) of the derivative **3a**; Figure S2: Anticancer activity (single-dose (10^−5^ M) assay) of the derivative **3b**; Figure S3: Anticancer activity (single-dose (10^−5^ M) assay) of the derivative **3c**; Figure S4: Anticancer activity (single-dose (10^−5^ M) assay) of the derivative **3d**; Figure S5: Anticancer activity (single-dose (10^−5^ M) assay) of the derivative **3e**; Figure S6: Anticancer activity (single-dose (10^−5^ M) assay) of the derivative **3f**; Figure S7: Anticancer activity (single-dose (10^−5^ M) assay) of the derivative **3g**; Figure S8: Anticancer activity (single-dose (10^−5^ M) assay) of the derivative **3h**; Figure S9: Anticancer activity (single-dose (10^−5^ M) assay) of the derivative **3i**; Figure S10: Anticancer activity (single-dose (10^−5^ M) assay) of the derivative **3j**; Figure S11: Anticancer activity (single-dose (10^−5^ M) assay) of the derivative **3k**; Figure S12: Anticancer activity (single-dose (10^−5^ M) assay) of the derivative **3l**; Figure S13: Anticancer activity (single-dose (10^−5^ M) assay) of the derivative **3m**; Figure S14: Anticancer activity (single-dose (10^−5^ M) assay) of the derivative **3n**; Figure S15. Anticancer activity (five-dose assay) of the azaheterocyclic derivative **3e**. Figure S16. Anticancer activity (five-dose assay) of the azaheterocyclic derivative **3h**. Figure S17. Anticancer activity (five-dose assay) of the azaheterocyclic derivative **3l**. Table S1. CLC-Pred data of the azaheterocyclic derivative **3a**; Table S2. CLC-Pred data of the azaheterocyclic derivative **3b**; Table S3. CLC-Pred data of the azaheterocyclic derivative **3c**; Table S4. CLC-Pred data of the azaheterocyclic derivative **3d**; Table S5. CLC-Pred data of the azaheterocyclic derivative **3e**; Table S6. CLC-Pred data of the azaheterocyclic derivative **3f**; Table S7. CLC-Pred data of the azaheterocyclic derivative **3g**; Table S8. CLC-Pred data of the azaheterocyclic derivative **3h**; Table S9. CLC-Pred data of the azaheterocyclic derivative **3i**; Table S10. CLC-Pred data of the azaheterocyclic derivative **3j**; Table S11. CLC-Pred data of the azaheterocyclic derivative **3k**; Table S12. CLC-Pred data of the azaheterocyclic derivative **3l**; Table S13. CLC-Pred data of the azahetetrocyclic derivative **3m**; Table S14. CLC-Pred data of the azaheterocyclic derivative **3n**.
