# Supplementary material for: Reinvestigating Pyrrol-2-One-Based Compounds: From Antimicrobial Agents to Promising Antitumor Candidates
Source: Pharmaceuticals (Basel). 2025 Nov 27;18(12):1813. doi: 10.3390/ph18121813 (PMC12736255; doi:10.3390/ph18121813)
Supplement: Supplementary file 1 [file pharmaceuticals-18-01813-s001.zip › pharmaceuticals-3985801-supplementary.pdf]

Supplementary file for

Reinvestigating Pyrrol-2-One-Based Compounds: From Antimicrobial Agents to Promising Antitumor Candidates

Natalia Simionescu[1,§], Ashraf Al-Matarneh[1,2,§], Ionel I. Mangalagiu[2], Narcis Cibotariu[1], Cristina Uritu[3], Cristina M. Al-Matarneh[1,*], Mariana Pinteala[1]

[1]**Center of Advanced Research in Bionanoconjugates and Biopolymers,** *"Petru Poni" Institute of Macromolecular Chemistry of Romanian Academy, 41A Grigore*

Ghica Voda Alley, Iasi 700487, Romania

[2]*Faculty of Chemistry, Alexandru Ioan Cuza University of Iasi, 11 Carol I, Iasi 700506, Romania;*

[3]*Advanced Center for Research and Development in Experimental Medicine*

"Prof. Ostin C. Mungiu", "Grigore T. Popa" University of Medicine and Pharmacy,

Iasi, Romania.

§ *authors with equal contribution*

*almatarneh.cristina@icmpp.ro

Developmental Therapeutics Program One Dose Bar Graph	NSC: D-852167 / 1	Conc: 1.000E-5 M	Test Date: Apr 22, 2024
	Experiment ID: 2404HT36		Report Date: Jun 4, 2024

Panel/Cell Line	Growth Percent
Leukemia	
CCRF-CEM	95.27
HL-60(TB)	114.67
K-562	100.38
MOLT-4	98.04
RPMI-8226	-40.26
SR	56.27
Non-Small Cell Lung Cancer	
A549/ATCC	95.04
EKVX	62.67
HOP-92	112.69
NCI-H226	96.17
NCI-H23	93.90
NCI-H322M	82.77
NCI-H460	99.31
NCI-H522	49.97
Colon Cancer	
COLO 205	103.81
HCC-2998	112.39
HCT-116	86.26
HCT-15	80.55
HT29	100.20
KM12	91.58
SW-620	104.00
CNS Cancer	
SF-268	83.74
SF-539	87.58
SNB-19	91.73
SNB-75	84.63
Melanoma	
LOX IMVI	81.95
MALME-3M	114.44
M14	113.71
MDA-MB-435	123.70
SK-MEL-2	73.57
SK-MEL-28	98.35
SK-MEL-5	89.97
UACC-257	87.12
UACC-62	102.61
Ovarian Cancer	
IGROV1	84.26
OVCAR-3	99.11
OVCAR-4	80.41
OVCAR-5	108.67
OVCAR-8	84.81
NCI/ADR-RES	100.88
SK-OV-3	91.92
Renal Cancer	
786-0	82.62
A498	83.71
ACHN	86.89
CAKI-1	97.03
SN12C	77.02
TK-10	87.53
Prostate Cancer	
PC-3	109.37
DU-145	113.47
Breast Cancer	
MCF7	56.26
MDA-MB-231/ATCC	110.03
HS 578T	28.94
T-47D	66.30

Bar Graph

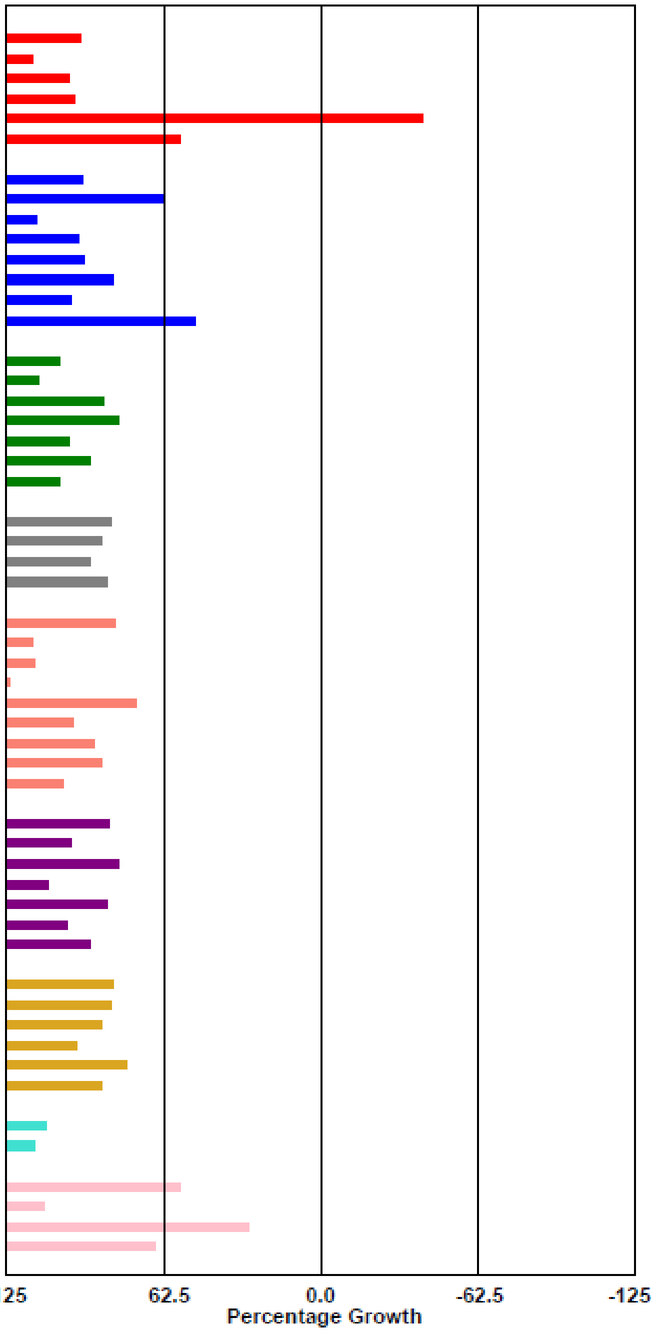

Figure S1: Anticancer activity (single-dose (10^{-5} M) assay) of the derivative **3a**;

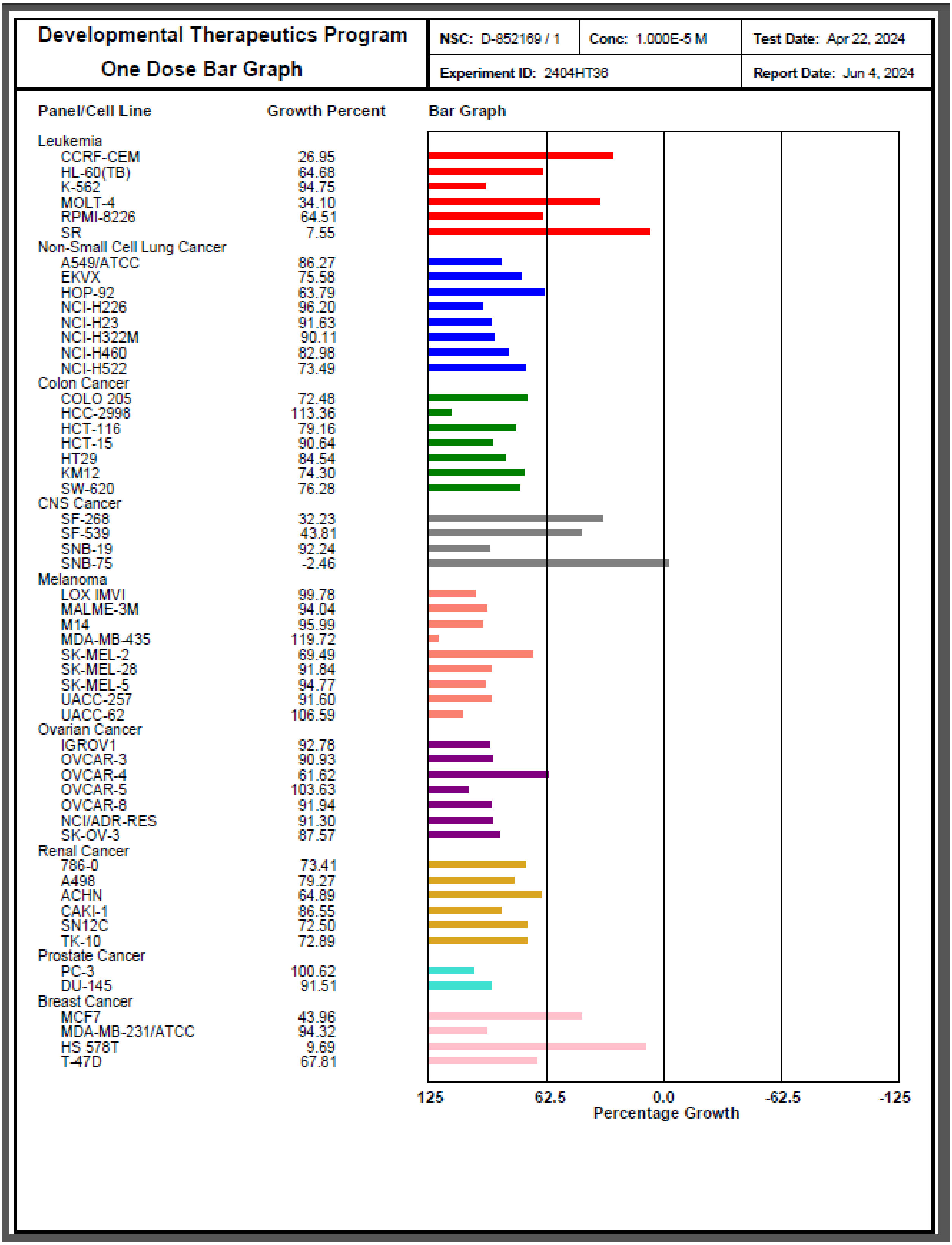

Figure S2: Anticancer activity (single-dose (10^{-5} M) assay) of the derivative **3b**;

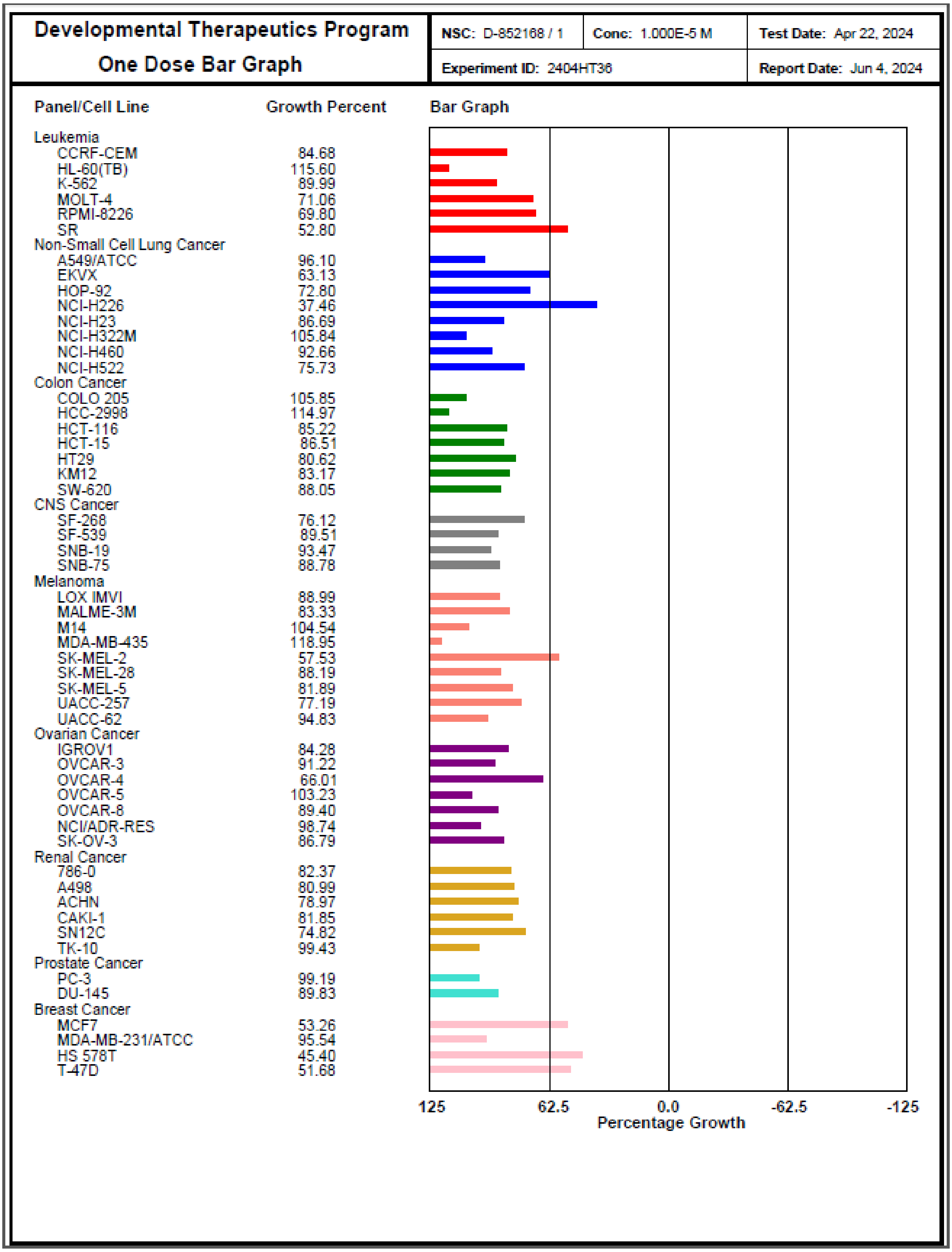

Figure S3: Anticancer activity (single-dose (10^{-5} M) assay) of the derivative **3c**;

Developmental Therapeutics Program<br>One Dose Bar Graph	NSC: D-852164 / 1	Conc: 1.000E-5 M	Test Date: Apr 22, 2024
	Experiment ID: 2404HT36		Report Date: Jun 4, 2024

Panel/Cell Line	Growth Percent
**Leukemia**	
CCRF-CEM	83.39
HL-60(TB)	111.07
K-562	84.66
MOLT-4	80.36
RPMI-8226	66.04
SR	101.82
**Non-Small Cell Lung Cancer**	
A549/ATCC	105.57
EKVX	63.95
HOP-92	105.47
NCI-H226	71.89
NCI-H23	97.27
NCI-H322M	129.13
NCI-H460	99.86
NCI-H522	97.88
**Colon Cancer**	
COLO 205	123.55
HCC-2998	124.05
HCT-116	80.51
HCT-15	86.71
HT29	86.45
KM12	82.65
SW-620	84.49
**CNS Cancer**	
SF-268	85.39
SF-539	107.77
SNB-19	94.51
SNB-75	96.61
**Melanoma**	
LOX IMVI	98.91
MALME-3M	102.40
M14	105.66
MDA-MB-435	121.20
SK-MEL-2	83.86
SK-MEL-28	87.81
SK-MEL-5	80.22
UACC-257	97.47
UACC-62	107.71
**Ovarian Cancer**	
IGROV1	84.43
OVCAR-3	94.70
OVCAR-4	69.97
OVCAR-5	114.18
OVCAR-8	103.85
NCI/ADR-RES	108.97
SK-OV-3	104.78
**Renal Cancer**	
786-0	93.71
A498	95.26
ACHN	84.67
CAKI-1	87.69
SN12C	84.74
TK-10	100.10
**Prostate Cancer**	
PC-3	103.56
DU-145	110.35
**Breast Cancer**	
MCF7	39.69
MDA-MB-231/ATCC	102.97
HS 578T	59.27
T-47D	50.80

Bar Graph

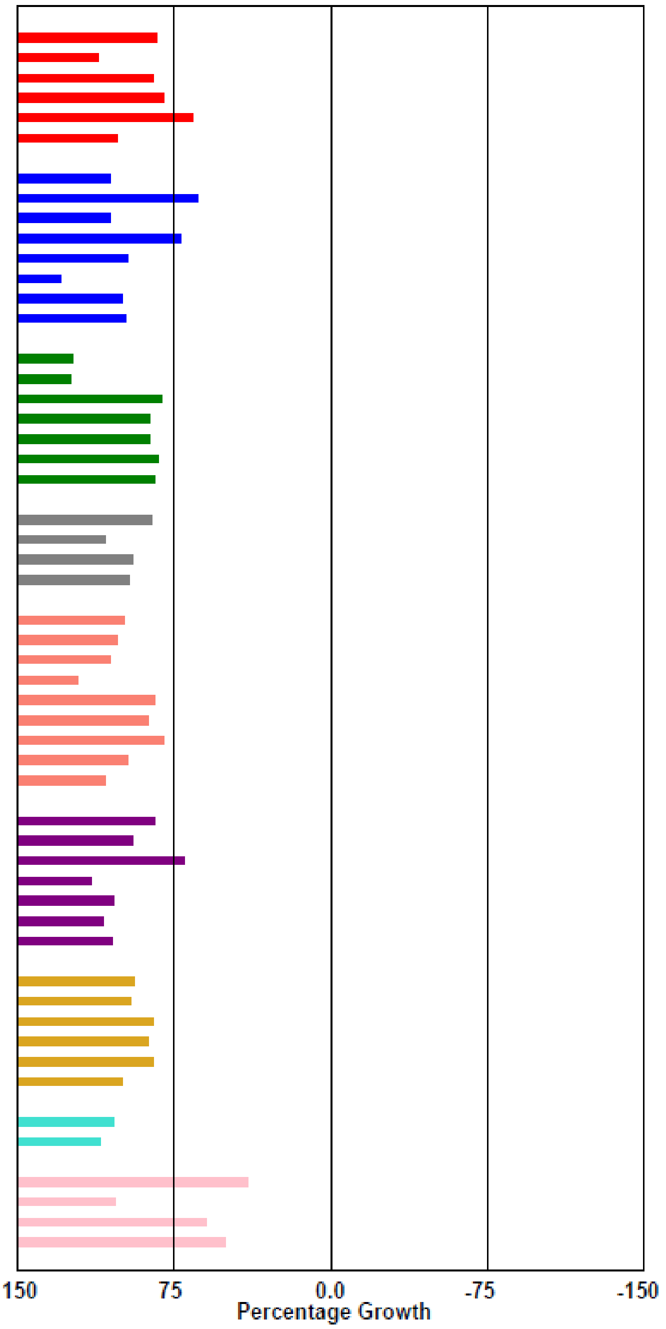

Figure S4: Anticancer activity (single-dose (10^{-5} M) assay) of the derivative **3d**;

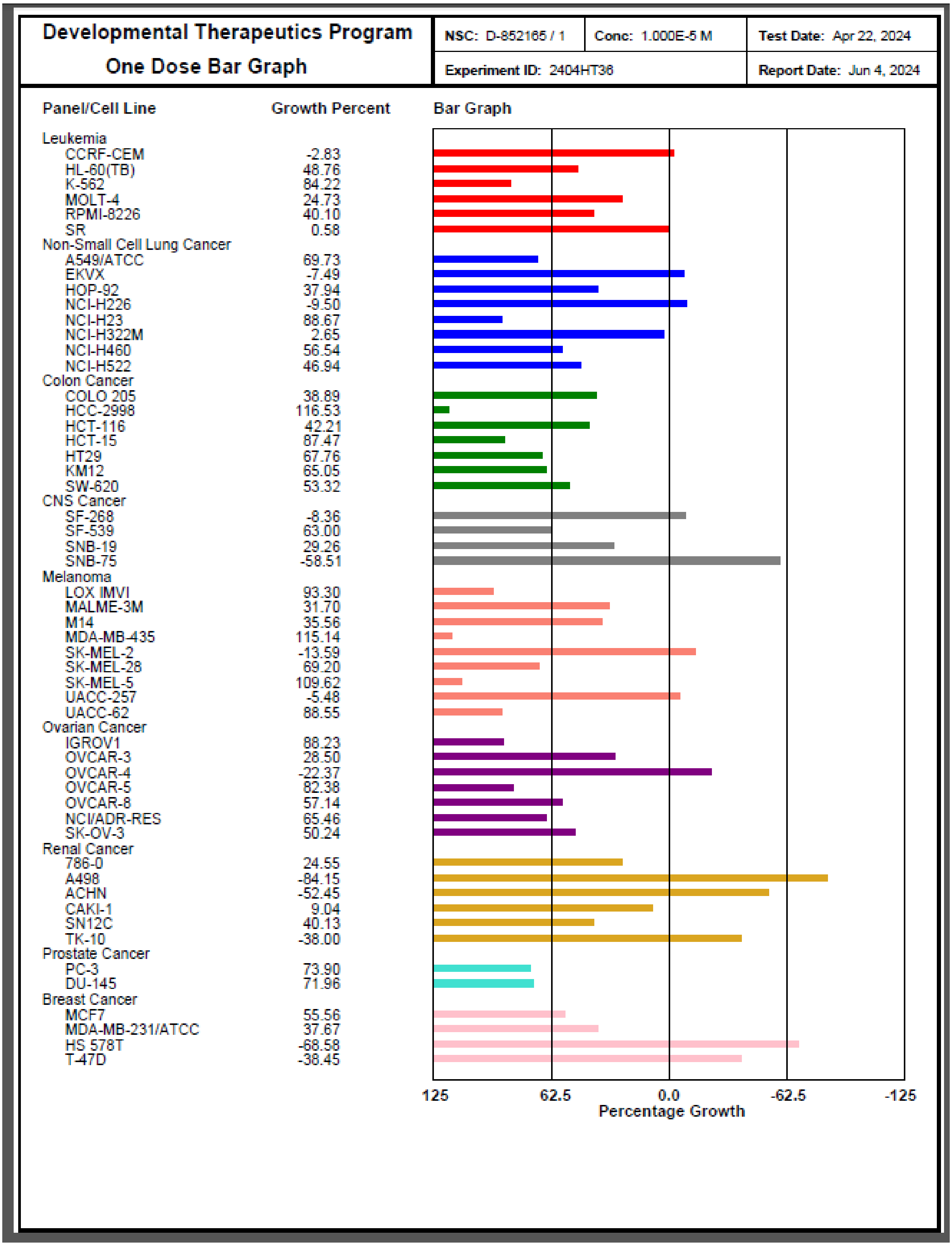

Figure S5: Anticancer activity (single-dose (10^{-5} M) assay) of the derivative **3e**;

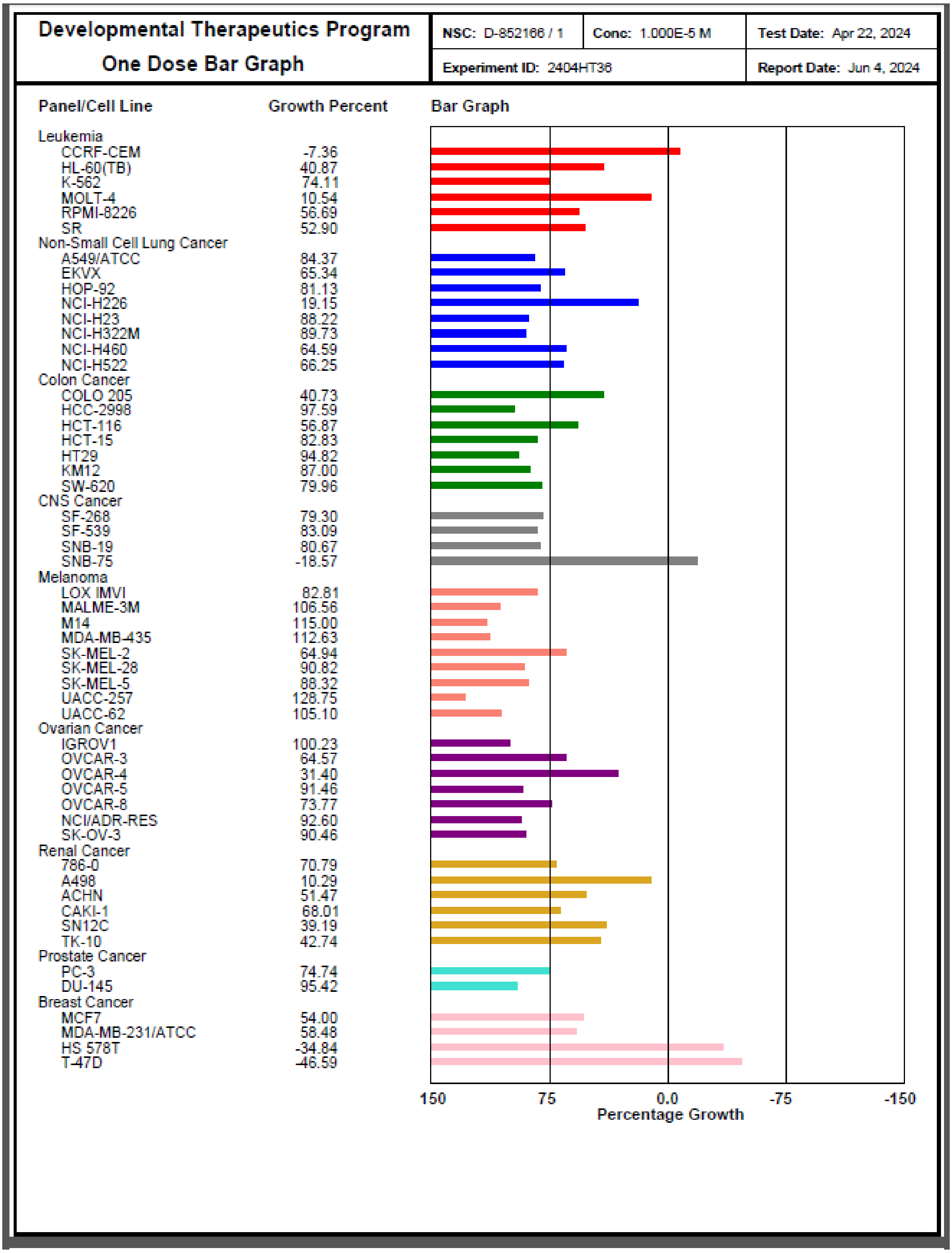

Figure S6: Anticancer activity (single-dose (10^{-5} M) assay) of the derivative **3f**;

Developmental Therapeutics Program

One Dose Bar Graph

**NSC:** D-852633 / 1	**Conc:** 1.000E-5 M	**Test Date:** Apr 29, 2024	
**Experiment ID:** 2404HT38		**Report Date:** Jun 4, 2024	

Panel/Cell Line	Growth Percent
**Leukemia**	
CCRF-CEM	5.60
HL-60(TB)	24.89
K-562	21.78
MOLT-4	-77.31
RPMI-8226	-1.36
SR	-7.50
**Non-Small Cell Lung Cancer**	
A549/ATCC	77.63
EKVX	36.19
HOP-62	95.11
HOP-92	34.34
NCI-H226	53.20
NCI-H23	55.08
NCI-H322M	80.12
NCI-H460	31.21
NCI-H522	30.81
**Colon Cancer**	
COLO 205	58.33
HCC-2998	46.61
HCT-116	38.54
HCT-15	16.49
HT29	4.30
KM12	20.20
SW-620	43.90
**CNS Cancer**	
SF-268	65.31
SF-295	51.70
SF-539	30.70
SNB-19	82.51
SNB-75	22.54
U251	70.88
**Melanoma**	
LOX IMVI	55.68
MALME-3M	17.14
M14	48.30
MDA-MB-435	62.06
SK-MEL-2	44.08
SK-MEL-28	57.56
SK-MEL-5	72.66
UACC-257	58.11
UACC-62	66.56
**Ovarian Cancer**	
IGROV1	72.52
OVCAR-3	40.74
OVCAR-4	15.23
OVCAR-5	75.77
OVCAR-8	69.27
NCI/ADR-RES	55.52
SK-OV-3	74.67
**Renal Cancer**	
A498	52.49
CAKI-1	58.65
SN12C	39.95
TK-10	56.53
UO-31	46.32
**Prostate Cancer**	
PC-3	26.70
DU-145	42.24
**Breast Cancer**	
MCF7	17.05
MDA-MB-231/ATCC	56.18
HS 578T	-0.46
BT-549	45.91
T-47D	20.38
MDA-MB-468	4.32

Bar Graph

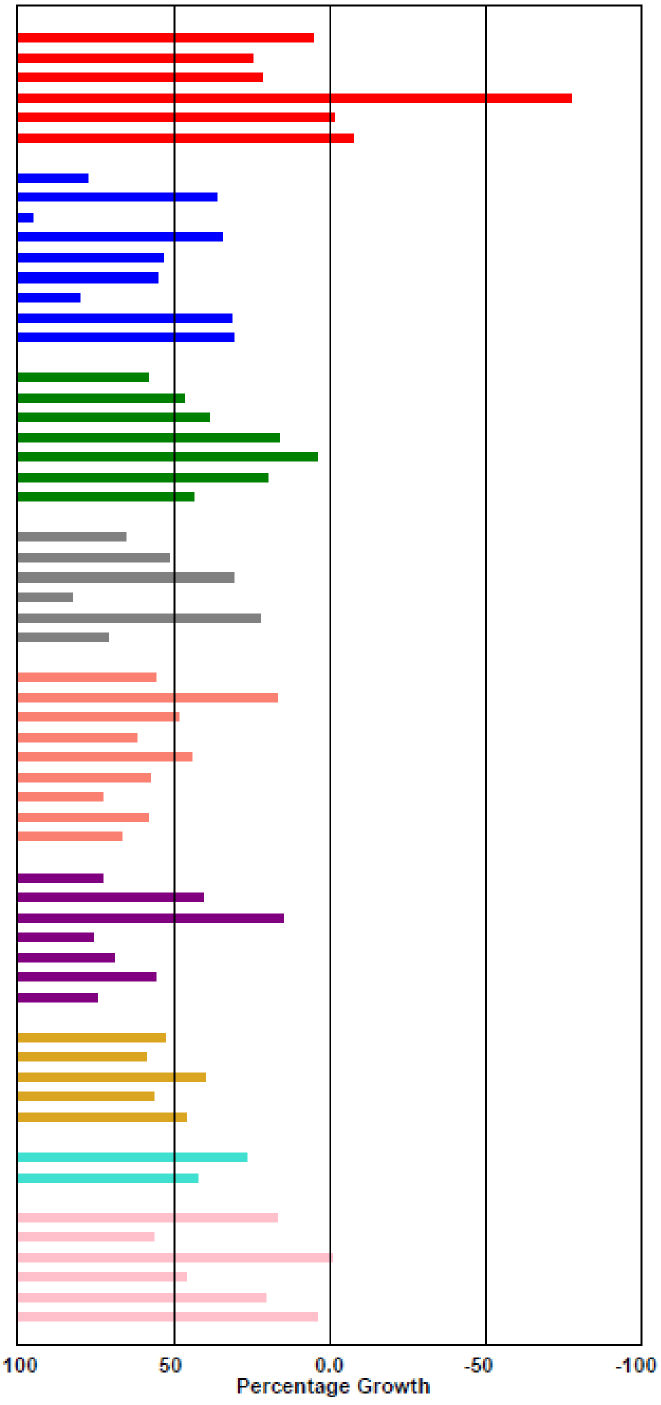

Figure S7: Anticancer activity (single-dose (10^{-5} M) assay) of the derivative **3g**;

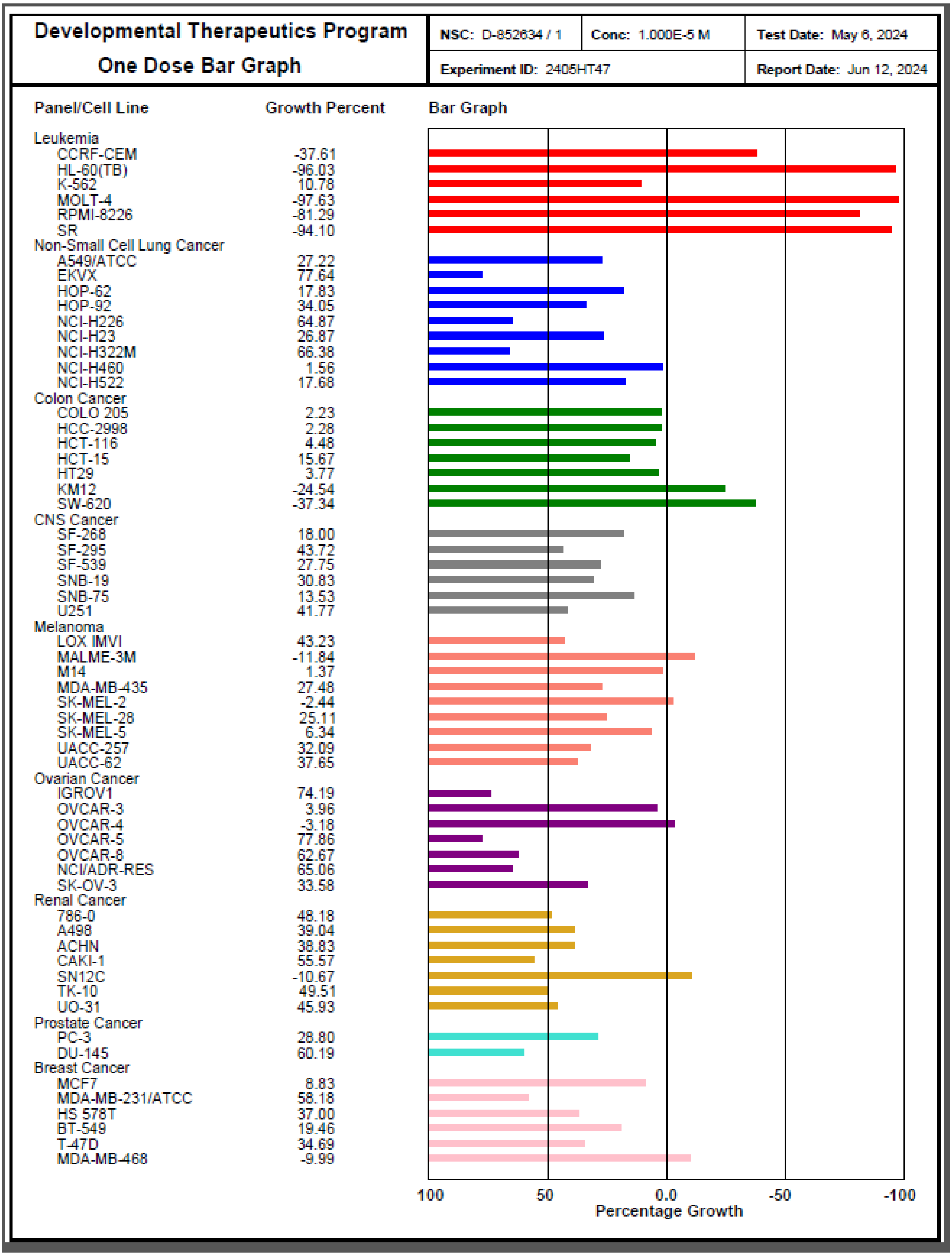

Figure S8: Anticancer activity (single-dose (10^{-5} M) assay) of the derivative **3h**;

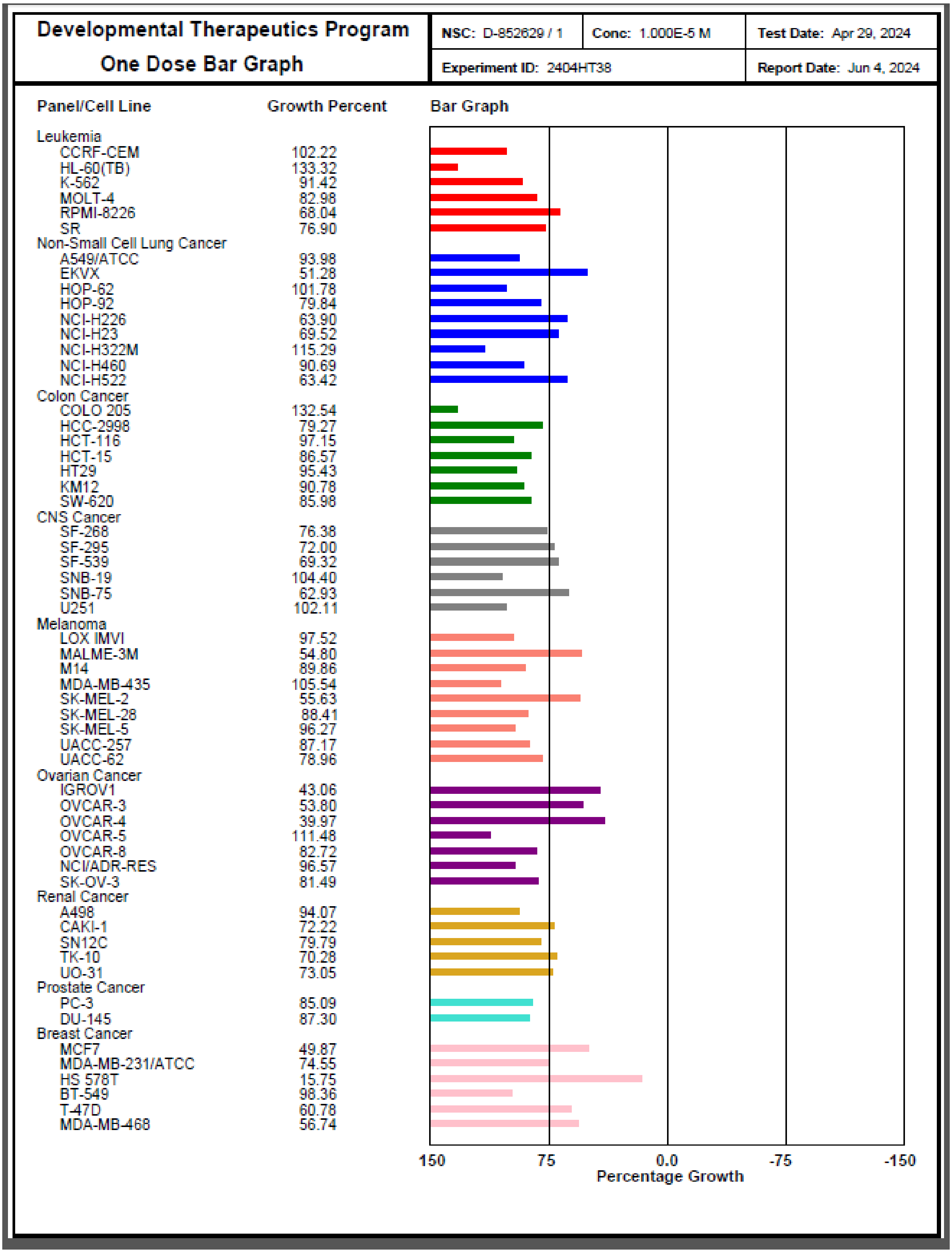

Figure S9: Anticancer activity (single-dose (10^{-5} M) assay) of the derivative **3i**;

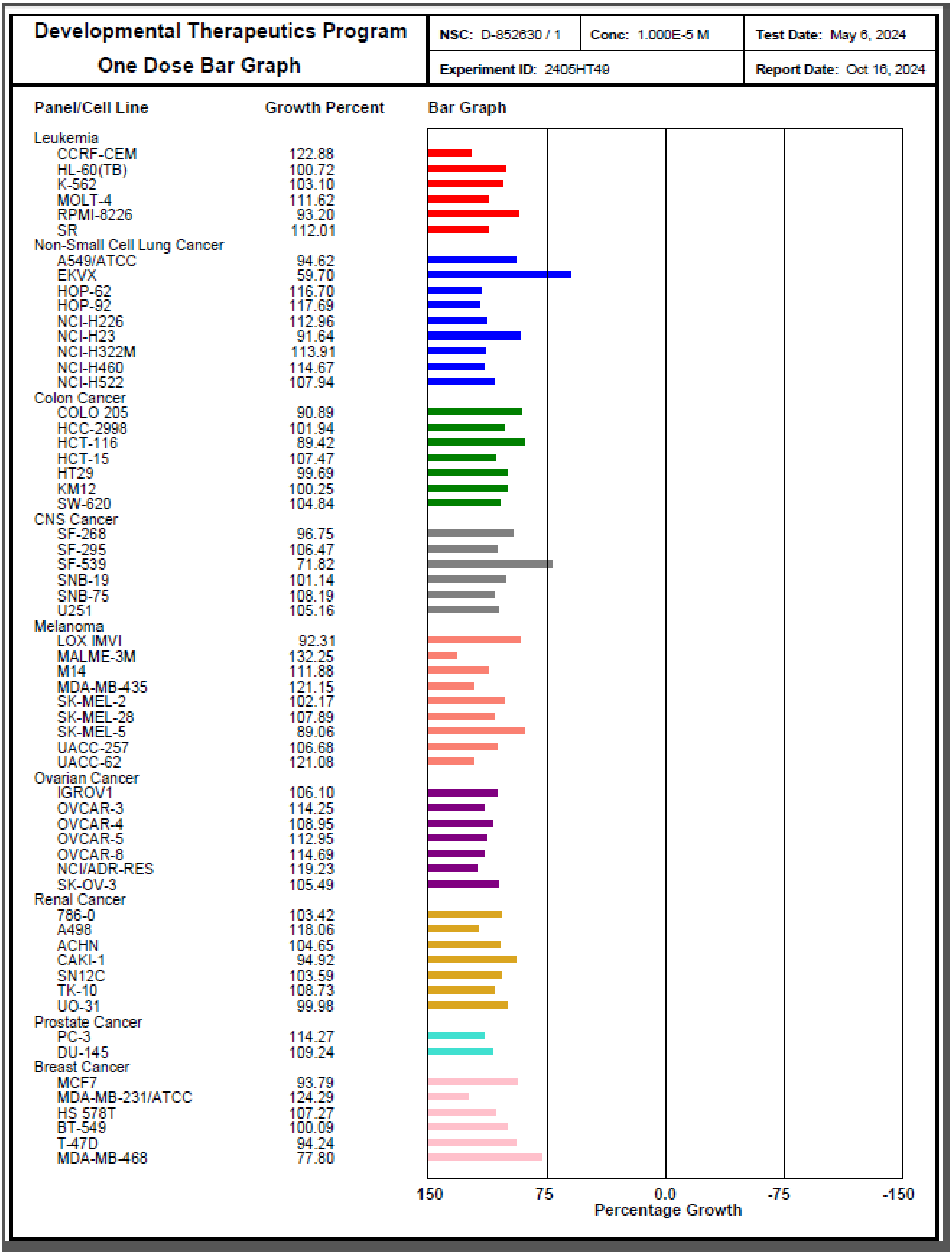

Developmental Therapeutics Program — One Dose Bar Graph

NSC: D-852630 / 1	Conc: 1.000E-5 M	Test Date: May 6, 2024
Experiment ID: 2405HT49		Report Date: Oct 16, 2024

Panel/Cell Line	Growth Percent
**Leukemia**	
CCRF-CEM	122.88
HL-60(TB)	100.72
K-562	103.10
MOLT-4	111.62
RPMI-8226	93.20
SR	112.01
**Non-Small Cell Lung Cancer**	
A549/ATCC	94.62
EKVX	59.70
HOP-62	116.70
HOP-92	117.69
NCI-H226	112.96
NCI-H23	91.64
NCI-H322M	113.91
NCI-H460	114.67
NCI-H522	107.94
**Colon Cancer**	
COLO 205	90.89
HCC-2998	101.94
HCT-116	89.42
HCT-15	107.47
HT29	99.69
KM12	100.25
SW-620	104.84
**CNS Cancer**	
SF-268	96.75
SF-295	106.47
SF-539	71.82
SNB-19	101.14
SNB-75	108.19
U251	105.16
**Melanoma**	
LOX IMVI	92.31
MALME-3M	132.25
M14	111.88
MDA-MB-435	121.15
SK-MEL-2	102.17
SK-MEL-28	107.89
SK-MEL-5	89.06
UACC-257	106.68
UACC-62	121.08
**Ovarian Cancer**	
IGROV1	106.10
OVCAR-3	114.25
OVCAR-4	108.95
OVCAR-5	112.95
OVCAR-8	114.69
NCI/ADR-RES	119.23
SK-OV-3	105.49
**Renal Cancer**	
786-0	103.42
A498	118.06
ACHN	104.65
CAKI-1	94.92
SN12C	103.59
TK-10	108.73
UO-31	99.98
**Prostate Cancer**	
PC-3	114.27
DU-145	109.24
**Breast Cancer**	
MCF7	93.79
MDA-MB-231/ATCC	124.29
HS 578T	107.27
BT-549	100.09
T-47D	94.24
MDA-MB-468	77.80

Figure S10: Anticancer activity (single-dose (10^{-5} M) assay) of the derivative **3j**;

Developmental Therapeutics Program
One Dose Bar Graph

NSC: D-852631 / 1	Conc: 1.000E-5 M	Test Date: May 6, 2024
Experiment ID: 2405HT49		Report Date: Oct 16, 2024

Panel/Cell Line	Growth Percent
Leukemia	
CCRF-CEM	122.83
HL-60(TB)	98.15
K-562	104.79
MOLT-4	105.82
RPMI-8226	85.79
SR	92.75
Non-Small Cell Lung Cancer	
A549/ATCC	97.01
EKVX	111.63
HOP-62	118.43
HOP-92	107.93
NCI-H226	115.09
NCI-H23	95.49
NCI-H322M	111.18
NCI-H460	112.46
NCI-H522	97.61
Colon Cancer	
COLO 205	109.32
HCC-2998	95.35
HCT-116	95.91
HCT-15	104.41
HT29	100.45
KM12	97.69
SW-620	100.23
CNS Cancer	
SF-268	99.30
SF-295	98.26
SF-539	82.01
SNB-19	101.26
SNB-75	99.63
U251	101.19
Melanoma	
LOX IMVI	94.81
MALME-3M	125.43
M14	106.10
MDA-MB-435	113.80
SK-MEL-2	101.52
SK-MEL-28	100.27
SK-MEL-5	88.01
UACC-257	117.51
UACC-62	111.25
Ovarian Cancer	
IGROV1	98.37
OVCAR-3	115.53
OVCAR-4	96.60
OVCAR-5	110.94
OVCAR-8	116.83
NCI/ADR-RES	110.61
SK-OV-3	99.99
Renal Cancer	
786-0	97.08
A498	121.64
ACHN	106.66
CAKI-1	85.12
SN12C	98.03
TK-10	98.99
UO-31	104.03
Prostate Cancer	
PC-3	108.39
DU-145	110.88
Breast Cancer	
MCF7	77.42
MDA-MB-231/ATCC	123.76
HS 578T	103.53
BT-549	89.64
T-47D	92.11
MDA-MB-468	56.56

Bar Graph

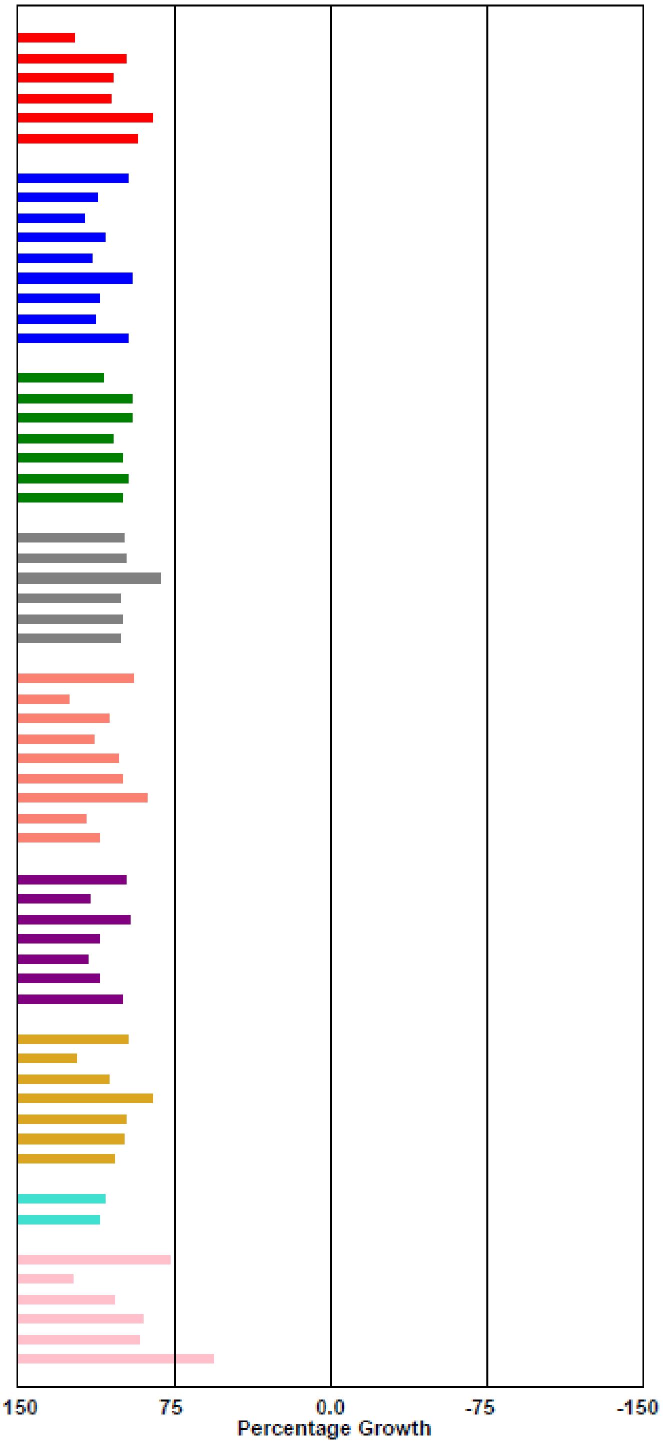

Figure S11: Anticancer activity (single-dose (10^{-5} M) assay) of the derivative **3k**;

Developmental Therapeutics Program One Dose Bar Graph	NSC: D-852170 / 1	Conc: 1.000E-5 M	Test Date: Apr 22, 2024
	Experiment ID: 2404HT36		Report Date: Jun 4, 2024

Panel/Cell Line	Growth Percent
**Leukemia**	
CCRF-CEM	26.49
HL-60(TB)	104.26
K-562	86.02
MOLT-4	22.39
RPMI-8226	17.25
SR	12.61
**Non-Small Cell Lung Cancer**	
A549/ATCC	68.80
EKVX	0.88
HOP-92	24.78
NCI-H226	-19.81
NCI-H23	64.10
NCI-H322M	48.85
NCI-H460	60.29
NCI-H522	68.40
**Colon Cancer**	
COLO 205	54.15
HCC-2998	50.34
HCT-116	52.27
HCT-15	79.24
HT29	60.31
KM12	47.66
SW-620	49.66
**CNS Cancer**	
SF-268	7.48
SF-539	65.12
SNB-19	65.15
SNB-75	-51.73
**Melanoma**	
LOX IMVI	10.73
MALME-3M	54.27
M14	37.30
MDA-MB-435	80.90
SK-MEL-2	-2.74
SK-MEL-28	70.70
SK-MEL-5	76.08
UACC-257	78.09
UACC-62	83.11
**Ovarian Cancer**	
IGROV1	45.82
OVCAR-3	40.16
OVCAR-4	-89.48
OVCAR-5	88.66
OVCAR-8	70.36
NCI/ADR-RES	62.66
SK-OV-3	25.02
**Renal Cancer**	
786-0	45.34
A498	12.46
ACHN	-9.55
CAKI-1	38.27
SN12C	40.83
TK-10	1.05
**Prostate Cancer**	
PC-3	55.34
DU-145	63.35
**Breast Cancer**	
MCF7	48.43
MDA-MB-231/ATCC	-11.56
HS 578T	-20.41
T-47D	41.13

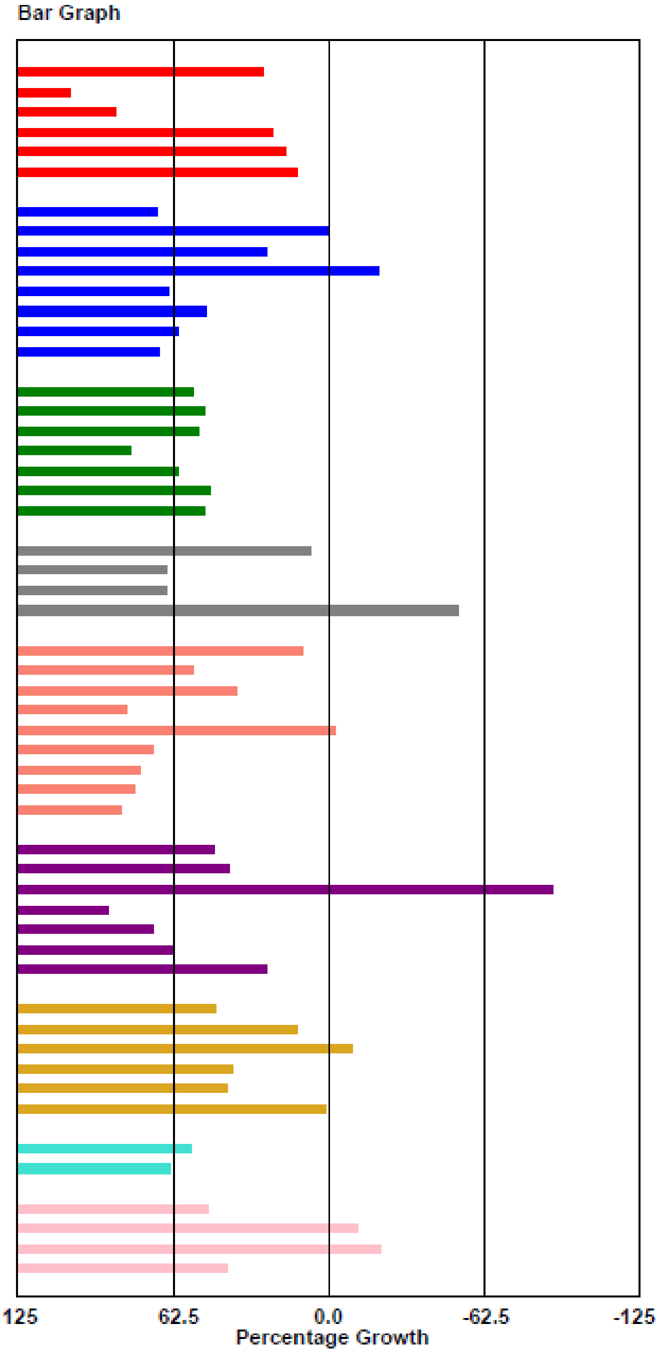

Figure S12: Anticancer activity (single-dose (10^{-5} M) assay) of the derivative **3l**;

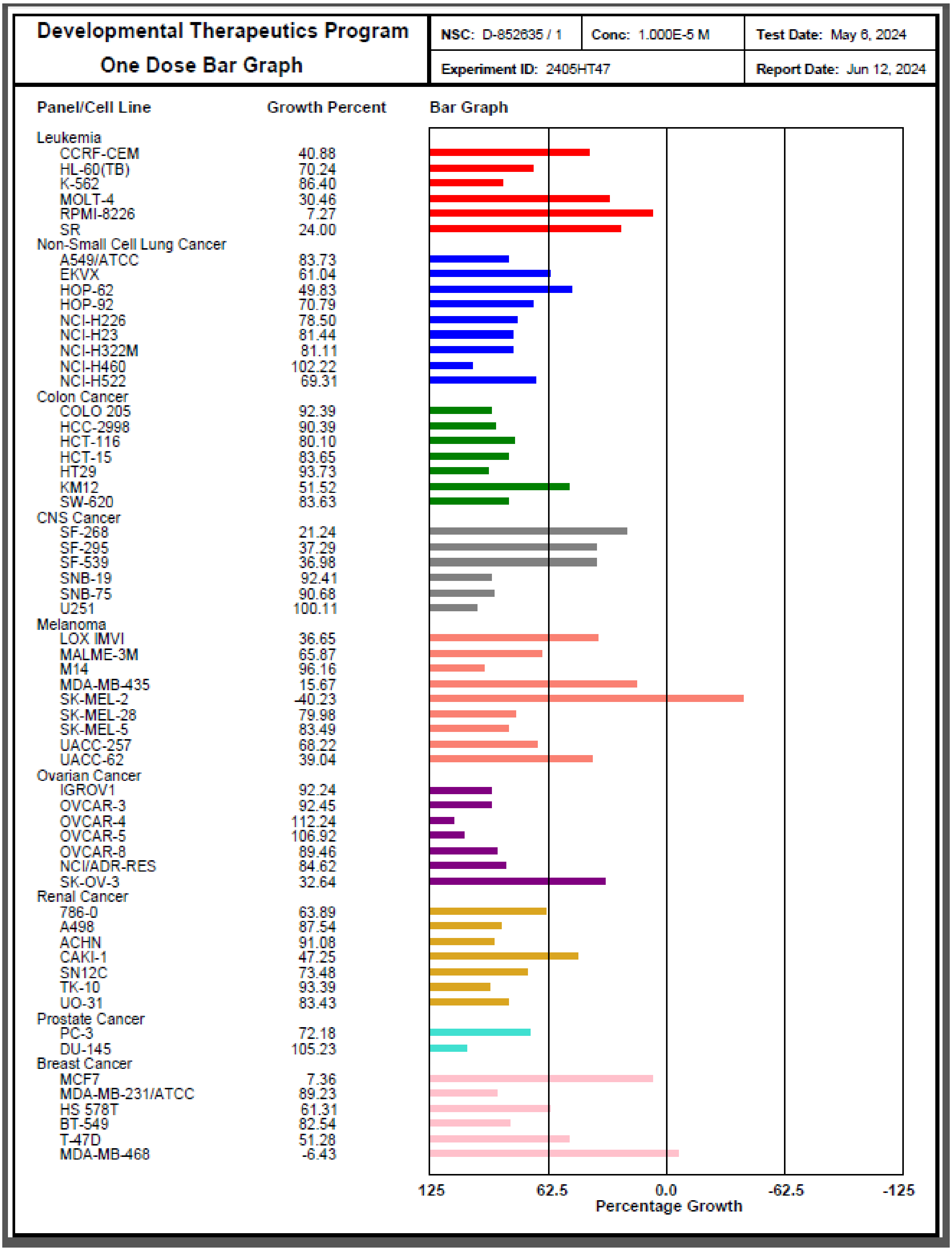

Figure S13: Anticancer activity (single-dose (10^{-5} M) assay) of the derivative **3m**;

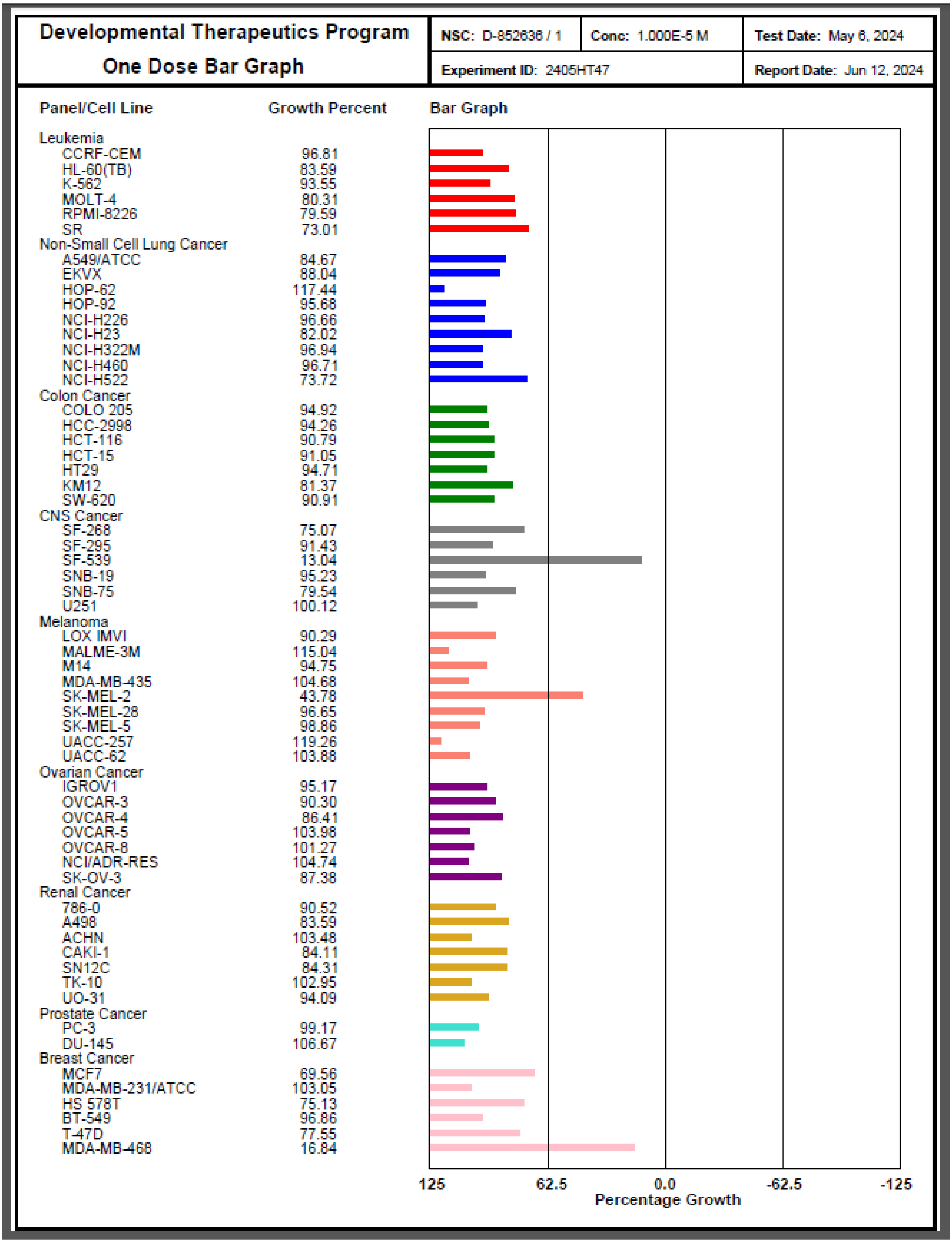

Developmental Therapeutics Program — One Dose Bar Graph

NSC: D-852636 / 1 | Conc: 1.000E-5 M | Test Date: May 6, 2024

Experiment ID: 2405HT47 | Report Date: Jun 12, 2024

Panel/Cell Line	Growth Percent
**Leukemia**	
CCRF-CEM	96.81
HL-60(TB)	83.59
K-562	93.55
MOLT-4	80.31
RPMI-8226	79.59
SR	73.01
**Non-Small Cell Lung Cancer**	
A549/ATCC	84.67
EKVX	88.04
HOP-62	117.44
HOP-92	95.68
NCI-H226	96.66
NCI-H23	82.02
NCI-H322M	96.94
NCI-H460	96.71
NCI-H522	73.72
**Colon Cancer**	
COLO 205	94.92
HCC-2998	94.26
HCT-116	90.79
HCT-15	91.05
HT29	94.71
KM12	81.37
SW-620	90.91
**CNS Cancer**	
SF-268	75.07
SF-295	91.43
SF-539	13.04
SNB-19	95.23
SNB-75	79.54
U251	100.12
**Melanoma**	
LOX IMVI	90.29
MALME-3M	115.04
M14	94.75
MDA-MB-435	104.68
SK-MEL-2	43.78
SK-MEL-28	96.65
SK-MEL-5	98.86
UACC-257	119.26
UACC-62	103.88
**Ovarian Cancer**	
IGROV1	95.17
OVCAR-3	90.30
OVCAR-4	86.41
OVCAR-5	103.98
OVCAR-8	101.27
NCI/ADR-RES	104.74
SK-OV-3	87.38
**Renal Cancer**	
786-0	90.52
A498	83.59
ACHN	103.48
CAKI-1	84.11
SN12C	84.31
TK-10	102.95
UO-31	94.09
**Prostate Cancer**	
PC-3	99.17
DU-145	106.67
**Breast Cancer**	
MCF7	69.56
MDA-MB-231/ATCC	103.05
HS 578T	75.13
BT-549	96.86
T-47D	77.55
MDA-MB-468	16.84

Figure S14: Anticancer activity (single-dose (10^{-5} M) assay) of the derivative **3n**;

National Cancer Institute Developmental Therapeutics Program<br>In-Vitro Testing Results			
NSC : D - 852165 / 1	Experiment ID : 2407HT57	Test Type : HTS	Units : Molar
Report Date : September 10, 2024	Test Date : July 22, 2024	QNS :	MC :
COMI : QC4	Stain Reagent :	SSPL : 0GLI	

			Log10 Concentration												
	Time		Mean Optical Densities					Percent Growth							
Panel/Cell Line	Zero	Ctrl	-8.0	-7.0	-6.0	-5.0	-4.0	-8.0	-7.0	-6.0	-5.0	-4.0	GI50	TGI	LC50
Leukemia															
CCRF-CEM	0.971	5.778	5.321	5.420	4.930	1.984	0.468	91	92	83	20	-52	* 3.35E-6	* 1.91E-5	* 9.45E-5
HL-60(TB)	0.502	2.873	2.885	2.994	2.769	1.109	0.115	100	105	96	26	-77	* 4.48E-6	* 1.78E-5	* 5.45E-5
K-562	0.421	7.233	7.355	7.132	6.858	6.374	5.020	102	98	95	87	67	> 1.00E-4	> 1.00E-4	> 1.00E-4
MOLT-4	0.728	3.915	3.762	3.953	3.117	1.395	0.545	95	101	75	21	-25	* 2.92E-6	* 2.84E-5	> 1.00E-4
RPMI-8226	4.586	10.694	11.390	11.370	10.692	6.518	2.639	111	111	100	32	-42	* 5.38E-6	* 2.67E-5	> 1.00E-4
SR	0.953	6.344	6.402	6.396	5.627	1.257	0.073	101	101	87	6	-92	* 2.84E-6	* 1.14E-5	* 3.70E-5
Non-Small Cell Lung Cancer															
A549/ATCC	0.392	3.908	4.085	4.031	4.004	2.334	0.558	105	104	103	55	5	* 1.27E-5	> 1.00E-4	> 1.00E-4
EKVX	5.120	12.856	12.355	13.122	12.338	10.700	4.036	94	103	93	72	-21	* 1.73E-5	* 5.93E-5	> 1.00E-4
HOP-62	1.512	4.540	4.836	4.602	4.809	3.848	.	110	102	109	77	.	> 1.00E-5	> 1.00E-5	> 1.00E-5
HOP-92	8.037	10.362	10.868	11.314	10.805	6.461	0.838	122	141	119	-20	-90	* 3.15E-6	* 7.22E-6	* 2.72E-5
NCI-H226	6.623	16.738	16.452	16.172	16.302	11.323	.	97	94	96	46	.	* 8.48E-6	> 1.00E-5	> 1.00E-5
NCI-H23	4.634	12.756	12.361	13.500	12.087	10.340	4.166	95	109	92	70	-10	* 1.79E-5	* 7.49E-5	> 1.00E-4
NCI-H322M	6.734	14.344	14.824	14.680	15.124	11.108	9.551	106	104	110	58	37	* 2.32E-5	> 1.00E-4	> 1.00E-4
NCI-H460	0.969	14.460	15.212	14.994	14.606	7.754	1.600	106	104	101	50	5	* 1.02E-5	> 1.00E-4	> 1.00E-4
NCI-H522	3.316	8.861	9.128	8.959	9.001	7.035	2.256	105	102	103	67	-32	* 1.49E-5	* 4.76E-5	> 1.00E-4
Colon Cancer															
COLO 205	0.835	4.041	4.519	4.626	4.321	1.467	0.597	115	118	109	20	-28	* 4.57E-6	* 2.57E-5	> 1.00E-4
HCC-2998	4.833	13.588	11.579	11.456	10.937	10.650	10.939	77	76	70	66	70	> 1.00E-4	> 1.00E-4	> 1.00E-4
HCT-116	0.373	4.693	4.952	4.327	3.963	2.111	0.738	106	92	83	40	8	* 5.92E-6	> 1.00E-4	> 1.00E-4
HCT-15	1.263	11.913	12.587	14.431	12.808	8.412	7.247	106	123	109	67	56	> 1.00E-4	> 1.00E-4	> 1.00E-4
HT29	0.509	4.912	4.807	5.057	4.674	3.652	2.080	98	103	95	71	36	* 3.97E-5	> 1.00E-4	> 1.00E-4
KM12	0.587	3.873	4.067	4.143	4.009	3.139	2.318	106	108	104	78	53	> 1.00E-4	> 1.00E-4	> 1.00E-4
SW-620	0.580	4.852	5.213	5.062	4.820	3.613	1.851	108	105	99	71	30	* 3.23E-5	> 1.00E-4	> 1.00E-4
CNS Cancer															
SF-268	1.084	3.232	3.040	3.218	3.256	1.613	0.449	91	99	101	25	-59	* 4.66E-6	* 1.98E-5	* 7.89E-5
SF-295	1.714	4.332	4.406	4.187	4.179	3.511	1.438	103	94	94	69	-16	* 1.66E-5	* 6.45E-5	> 1.00E-4
SF-539	4.105	12.554	13.243	13.136	12.349	12.265	6.851	108	107	98	96	32	* 5.33E-5	> 1.00E-4	> 1.00E-4
SNB-19	1.783	4.810	5.044	4.735	4.716	3.821	1.141	108	98	97	67	-36	* 1.47E-5	* 4.48E-5	> 1.00E-4
SNB-75	2.056	3.045	3.135	3.125	3.259	1.005	0.085	109	108	122	-51	-96	* 2.60E-6	* 5.06E-6	* 9.85E-6
U251	0.623	3.611	3.999	3.922	3.743	2.036	0.419	113	110	104	47	-33	* 8.97E-6	* 3.90E-5	> 1.00E-4
Melanoma															
LOX IMVI	0.362	2.388	1.996	1.502	2.444	2.261	1.676	81	56	103	94	65	> 1.00E-4	> 1.00E-4	> 1.00E-4
MALME-3M	2.638	4.344	4.532	4.490	4.269	1.201	0.802	111	108	95	-54	-70	* 2.00E-6	* 4.32E-6	* 9.34E-6
M14	4.190	12.645	13.516	12.848	13.474	8.142	1.094	110	102	110	47	-74	* 8.94E-6	* 2.45E-5	* 6.34E-5
MDA-MB-435	1.129	3.754	4.130	4.276	4.068	3.800	1.939	114	120	112	102	31	* 5.37E-5	> 1.00E-4	> 1.00E-4
SK-MEL-2	2.588	5.976	5.592	5.766	5.440	4.795	1.038	89	94	84	65	-60	* 1.32E-5	* 3.32E-5	* 8.33E-5
SK-MEL-28	1.628	5.149	5.085	5.032	5.085	4.500	1.822	98	97	98	82	6	* 2.60E-5	> 1.00E-4	> 1.00E-4
SK-MEL-5	2.807	8.771	8.899	8.207	8.431	7.508	8.317	103	91	94	79	93	> 1.00E-4	> 1.00E-4	> 1.00E-4
UACC-257	4.071	9.092	9.573	8.928	9.086	7.330	2.871	110	97	100	65	-30	* 1.44E-5	* 4.87E-5	> 1.00E-4
UACC-62	0.765	3.448	3.823	3.677	3.442	3.480	2.013	114	109	100	101	46	* 8.64E-5	> 1.00E-4	> 1.00E-4
Ovarian Cancer															
IGROV1	1.599	4.239	4.381	4.276	4.116	3.435	.	105	101	95	70	.	> 1.00E-5	> 1.00E-5	> 1.00E-5
OVCAR-3	2.879	11.483	11.693	11.557	11.866	6.500	0.626	103	101	104	42	-78	* 7.48E-6	* 2.24E-5	* 5.83E-5
OVCAR-4	4.638	8.145	7.959	7.721	7.580	2.497	0.221	95	88	84	-46	-95	* 1.82E-6	* 4.41E-6	* 1.20E-5
OVCAR-5	5.432	11.759	12.204	12.292	12.052	11.176	5.880	107	108	105	91	7	* 3.07E-5	> 1.00E-4	> 1.00E-4
OVCAR-8	0.988	4.758	4.571	4.624	4.543	2.880	0.810	95	96	94	50	-18	* 1.01E-5	* 5.43E-5	> 1.00E-4
NCI/ADR-RES	3.228	11.002	11.858	11.586	10.956	8.068	11.061	111	107	99	62	99	> 1.00E-4	> 1.00E-4	> 1.00E-4
SK-OV-3	2.770	12.794	12.391	12.486	11.227	9.966	14.185	96	97	84	72	115	> 1.00E-4	> 1.00E-4	> 1.00E-4
Renal Cancer															
786-0	1.731	.	.	.	.	.	.	.	.	.	.	.	.	.	.
A498	2.609	8.743	9.289	9.421	9.076	0.373	0.042	109	111	105	-86	-98	* 1.95E-6	* 3.56E-6	* 6.50E-6
ACHN	1.003	4.360	4.260	4.133	4.242	1.684	0.167	96	93	96	20	-83	* 4.05E-6	* 1.57E-5	* 4.77E-5
CAKI-1	1.015	4.749	4.697	4.598	4.434	1.713	0.277	99	96	92	19	-73	* 3.72E-6	* 1.60E-5	* 5.65E-5
RXF 393	2.078	3.544	3.483	3.509	3.449	2.432	2.146	96	98	94	24	5	* 4.23E-6	> 1.00E-4	> 1.00E-4
SN12C	0.660	2.686	2.688	2.783	2.697	2.034	0.725	100	105	101	68	3	* 1.89E-5	> 1.00E-4	> 1.00E-4
TK-10	7.459	17.461	16.994	16.955	17.156	7.814	0.646	95	95	97	3	-91	* 3.17E-6	* 1.08E-5	* 3.66E-5
UO-31	1.590	6.064	6.013	5.910	5.851	4.578	.	99	97	95	67	.	> 1.00E-5	> 1.00E-5	> 1.00E-5
Prostate Cancer															
PC-3	4.255	11.426	11.759	12.049	11.864	7.831	4.501	105	109	106	50	3	* 9.99E-6	> 1.00E-4	> 1.00E-4
DU-145	0.789	3.473	3.745	3.509	3.568	2.812	1.373	110	101	104	75	22	* 2.98E-5	> 1.00E-4	> 1.00E-4
Breast Cancer															
MCF7	0.974	8.476	8.418	7.463	7.634	5.003	2.374	98	86	89	54	19	* 1.28E-5	> 1.00E-4	> 1.00E-4
MDA-MB-231/ATCC	4.073	9.051	9.517	9.360	9.140	5.610	1.119	109	106	102	31	-72	* 5.36E-6	* 1.99E-5	* 6.05E-5
HS 578T	2.251	4.094	4.215	4.190	4.249	2.247	0.134	107	105	108	-0	-94	* 3.45E-6	* 9.97E-6	* 3.40E-5
BT-549	6.928	12.569	13.506	12.596	12.959	9.699	1.879	117	101	107	49	-73	* 9.61E-6	* 2.52E-5	* 6.49E-5
T-47D	6.839	11.355	11.509	11.871	11.334	5.902	5.902	104	111	100	-14	-14	* 2.74E-6	* 7.57E-6	> 1.00E-4
MDA-MB-468	5.295	9.590	9.580	9.478	9.372	5.592	.	100	97	95	7	.	* 3.24E-6	> 1.00E-5	> 1.00E-5

Figure S15. Anticancer activity (five-dose assay) of the azatetracyclic derivative **3e**.

National Cancer Institute Developmental Therapeutics Program
In-Vitro Testing Results

NSC : D - 852634 / 1	Experiment ID : 2407HT60	Test Type : HTS	Units : Molar
Report Date : September 11, 2024	Test Date : July 29, 2024	QNS :	MC :
COMI : QC7	Stain Reagent :	SSPL : 0GLI	

Log10 Concentration

Panel/Cell Line	Time Zero	Ctrl	Mean Optical Densities -8.0	-7.0	-6.0	-5.0	-4.0	Percent Growth -8.0	-7.0	-6.0	-5.0	-4.0	GI50	TGI	LC50
**Leukemia**															
CCRF-CEM	0.676	4.286	4.021	3.868	3.801	1.118	0.005	93	88	86	12	-99	* 3.09E-6	* 1.28E-5	* 3.61E-5
HL-60(TB)	0.587	2.824	3.539	3.782	3.001	0.216	0.008	131	144	108	-63	-99	* 2.19E-6	* 4.28E-6	* 8.38E-6
K-562	0.506	7.390	6.807	6.659	6.973	2.725	0.009	92	89	94	32	-98	* 5.15E-6	* 1.77E-5	* 4.27E-5
MOLT-4	0.700	3.609	4.233	4.098	3.694	0.259	0.004	121	117	103	-63	-99	* 2.08E-6	* 4.17E-6	* 8.35E-6
RPMI-8226	4.400	10.261	10.517	10.721	10.130	3.180	0.010	104	108	98	-28	-100	* 2.40E-6	* 6.01E-6	* 2.04E-5
SR	0.057	0.620	0.589	0.698	0.529	0.004	0.000	94	114	84	-92	-100	* 1.55E-6	* 2.99E-6	* 5.75E-6
**Non-Small Cell Lung Cancer**															
A549/ATCC	0.612	4.388	4.244	4.509	4.377	2.763	0.005	96	103	100	57	-99	* 1.11E-5	* 2.32E-5	* 4.84E-5
EKVX	2.594	7.333	7.674	7.911	7.112	5.248	0.025	107	112	95	56	-99	* 1.09E-5	* 2.30E-5	* 4.83E-5
HOP-62	1.049	3.448	3.450	3.703	4.018	2.496	0.008	100	111	124	60	-99	* 1.16E-5	* 2.39E-5	* 4.92E-5
HOP-92	8.768	10.268	11.236	10.987	10.802	8.165	0.021	165	145	139	-7	-100	* 4.08E-6	* 8.97E-6	* 2.91E-5
NCI-H226	6.508	13.383	14.346	14.632	14.853	11.095	0.013	114	118	121	67	-100	* 1.26E-5	* 2.52E-5	* 5.02E-5
NCI-H23	4.317	11.185	10.373	11.524	10.258	7.041	0.016	88	105	86	40	-100	* 6.02E-6	* 1.93E-5	* 4.40E-5
NCI-H322M	4.446	11.942	12.870	13.259	13.145	9.428	0.090	112	118	116	66	-98	* 1.26E-5	* 2.54E-5	* 5.11E-5
NCI-H460	1.246	18.944	18.715	19.519	19.390	8.053	0.027	99	103	103	38	-98	* 6.60E-6	* 1.92E-5	* 4.46E-5
NCI-H522	3.641	9.201	9.116	9.423	8.985	7.203	0.009	98	104	96	64	-100	* 1.22E-5	* 2.46E-5	* 4.97E-5
**Colon Cancer**															
COLO 205	1.187	4.714	4.387	4.945	5.019	2.505	0.008	91	107	109	37	-99	* 6.65E-6	* 1.88E-5	* 4.36E-5
HCC-2998	1.982	5.441	7.832	9.946	5.638	2.391	0.011	169	230	106	12	-100	* 3.92E-6	* 1.28E-5	* 3.59E-5
HCT-116	0.434	4.709	4.846	4.628	4.493	2.287	0.006	103	98	95	43	-99	* 7.43E-6	* 2.02E-5	* 4.54E-5
HCT-15	1.389	11.919	13.056	12.185	12.593	4.608	0.052	111	103	106	31	-96	* 5.55E-6	* 1.74E-5	* 4.32E-5
HT29	0.630	5.226	5.347	5.347	5.553	2.772	0.007	103	103	107	47	-99	* 8.80E-6	* 2.09E-5	* 4.61E-5
KM12	0.537	3.106	3.365	3.502	2.986	1.364	0.005	110	115	95	32	-99	* 5.22E-6	* 1.76E-5	* 4.23E-5
SW-620	0.598	4.200	4.499	4.657	4.608	3.393	0.008	108	113	111	78	-99	* 1.43E-5	* 2.76E-5	* 5.29E-5
**CNS Cancer**															
SF-268	0.654	2.157	2.321	2.150	2.265	1.165	0.003	111	100	107	34	-100	* 6.04E-6	* 1.80E-5	* 4.26E-5
SF-295	1.921	4.564	4.884	4.502	4.509	4.041	0.010	112	98	98	80	-100	* 1.47E-5	* 2.79E-5	* 5.30E-5
SF-539	3.656	12.884	13.990	13.274	13.132	9.427	0.023	112	104	103	62	-99	* 1.19E-5	* 2.43E-5	* 4.95E-5
SNB-19	1.925	4.811	5.052	5.219	5.102	4.070	0.021	108	114	110	74	-99	* 1.38E-5	* 2.69E-5	* 5.22E-5
SNB-75	1.257	2.396	2.350	2.498	2.507	2.001	0.006	96	109	110	65	-100	* 1.24E-5	* 2.49E-5	* 5.01E-5
U251	0.750	4.047	4.543	4.608	4.470	2.688	0.007	115	117	113	59	-99	* 1.14E-5	* 2.36E-5	* 4.89E-5
**Melanoma**															
LOX IMVI	0.607	3.513	3.757	3.742	3.864	2.161	0.005	108	108	112	54	-99	* 1.05E-5	* 2.24E-5	* 4.76E-5
MALME-3M	6.325	8.944	8.932	8.737	8.535	6.431	0.020	100	92	84	4	-100	* 2.68E-6	* 1.10E-5	* 3.32E-5
M14	4.271	11.958	12.653	12.636	11.935	6.682	0.027	109	109	100	31	-99	* 5.34E-6	* 1.74E-5	* 4.19E-5
MDA-MB-435	1.473	4.874	4.876	4.937	4.841	3.330	0.011	100	102	99	55	-99	* 1.07E-5	* 2.26E-5	* 4.79E-5
SK-MEL-2	2.519	5.550	5.394	5.241	5.304	3.762	0.011	95	90	92	41	-100	* 6.64E-6	* 1.96E-5	* 4.44E-5
SK-MEL-28	1.431	3.164	3.348	3.312	3.200	2.431	0.003	111	109	102	58	-100	* 1.12E-5	* 2.32E-5	* 4.83E-5
SK-MEL-5	3.825	11.459	10.558	12.454	11.706	7.784	0.020	88	113	103	52	-100	* 1.03E-5	* 2.20E-5	* 4.71E-5
UACC-257	3.486	7.298	8.310	7.991	7.658	5.344	0.007	127	118	109	49	-100	* 9.56E-6	* 2.13E-5	* 4.62E-5
UACC-62	0.739	3.049	3.172	3.074	3.037	2.210	0.003	105	101	100	64	-100	* 1.21E-5	* 2.45E-5	* 4.97E-5
**Ovarian Cancer**															
IGROV1	1.018	3.394	3.607	3.839	3.487	2.657	0.009	109	119	104	69	-99	* 1.30E-5	* 2.57E-5	* 5.10E-5
OVCAR-3	4.654	18.685	17.897	19.554	18.084	11.412	0.040	94	106	96	48	-99	* 9.16E-6	* 2.12E-5	* 4.64E-5
OVCAR-4	5.328	9.372	9.967	9.884	8.686	6.083	0.030	115	113	83	19	-99	* 3.27E-6	* 1.44E-5	* 3.82E-5
OVCAR-5	6.474	14.805	14.593	14.806	14.869	14.655	0.028	97	100	101	98	-100	* 1.75E-5	* 3.14E-5	* 5.62E-5
OVCAR-8	0.673	3.725	4.003	3.751	3.714	2.793	0.007	109	101	100	70	-99	* 1.30E-5	* 2.59E-5	* 5.12E-5
NCI/ADR-RES	3.746	10.491	12.023	11.660	11.838	9.235	0.062	123	117	120	81	-98	* 1.49E-5	* 2.84E-5	* 5.38E-5
SK-OV-3	2.611	9.797	10.261	9.565	9.043	5.817	0.009	106	97	90	45	-100	* 7.60E-6	* 2.04E-5	* 4.53E-5
**Renal Cancer**															
786-0	1.778	6.495	6.248	6.427	6.136	4.683	0.013	95	99	92	62	-99	* 1.18E-5	* 2.41E-5	* 4.94E-5
A498	1.927	7.756	8.872	8.185	8.177	4.933	0.018	119	108	107	52	-99	* 1.02E-5	* 2.20E-5	* 4.72E-5
ACHN	1.537	6.486	6.219	6.281	6.357	5.003	0.022	95	96	97	70	-99	* 1.31E-5	* 2.60E-5	* 5.15E-5
CAKI-1	1.045	4.167	4.402	4.408	4.264	3.153	0.010	108	108	103	68	-99	* 1.27E-5	* 2.54E-5	* 5.08E-5
RXF 393	1.802	3.005	3.172	3.149	3.157	2.209	0.006	114	112	113	34	-100	* 6.25E-6	* 1.80E-5	* 4.25E-5
SN12C	0.623	2.418	2.618	2.611	2.697	1.190	0.004	111	111	116	31	-99	* 6.01E-6	* 1.74E-5	* 4.20E-5
TK-10	6.290	16.819	16.983	17.396	16.823	13.372	0.017	102	105	100	67	-100	* 1.27E-5	* 2.53E-5	* 5.04E-5
UO-31	1.550	5.879	5.608	5.647	5.320	4.221	0.022	94	95	87	62	-99	* 1.18E-5	* 2.43E-5	* 4.97E-5
**Prostate Cancer**															
PC-3	3.978	11.351	12.223	12.186	12.526	7.384	0.025	112	111	116	46	-99	* 8.83E-6	* 2.08E-5	* 4.58E-5
DU-145	0.636	3.759	3.762	3.814	3.740	2.864	0.006	100	102	99	71	-99	* 1.34E-5	* 2.63E-5	* 5.16E-5
**Breast Cancer**															
MCF7	2.550	15.066	11.991	11.640	10.989	5.393	0.027	76	73	68	23	-99	* 2.46E-6	* 1.54E-5	* 3.96E-5
MDA-MB-231/ATCC	5.261	12.471	12.454	12.416	12.381	10.243	0.046	100	99	99	69	-99	* 1.30E-5	* 2.58E-5	* 5.11E-5
HS 578T	1.006	2.714	2.597	3.115	2.709	1.674	0.005	93	123	100	39	-100	* 6.65E-6	* 1.92E-5	* 4.40E-5
BT-549	3.822	8.044	8.767	8.677	8.297	6.560	0.020	117	115	106	65	-100	* 1.23E-5	* 2.48E-5	* 4.99E-5
T-47D	5.838	11.540	11.038	10.890	9.969	6.614	0.025	91	89	72	14	-100	* 2.41E-6	* 1.32E-5	* 3.65E-5
MDA-MB-468	6.310	10.749	10.479	10.313	9.743	7.272	0.045	94	90	77	22	-99	* 3.10E-6	* 1.51E-5	* 3.91E-5

Figure S16. Anticancer activity (five-dose assay) of the azatetracyclic derivative **3h**.

National Cancer Institute Developmental Therapeutics Program<br>In-Vitro Testing Results			
NSC : D - 852170 / 1	Experiment ID : 2407HT57	Test Type : HTS	Units : Molar
Report Date : September 10, 2024	Test Date : July 22, 2024	QNS :	MC :
COMI : QC20	Stain Reagent :	SSPL : 0GLI	

			Log10 Concentration												
	Time		Mean Optical Densities					Percent Growth							
Panel/Cell Line	Zero	Ctrl	-8.0	-7.0	-6.0	-5.0	-4.0	-8.0	-7.0	-6.0	-5.0	-4.0	GI50	TGI	LC50
Leukemia															
CCRF-CEM	0.971	5.778	5.142	5.338	4.407	2.414	0.885	87	91	71	30	-9	3.26E-6	5.84E-5	> 1.00E-4
HL-60(TB)	0.502	2.873	2.724	2.895	2.350	1.854	1.164	94	101	78	57	28	1.73E-5	> 1.00E-4	> 1.00E-4
K-562	0.421	7.233	6.606	6.185	6.533	5.656	4.522	91	84	90	77	60	> 1.00E-4	> 1.00E-4	> 1.00E-4
MOLT-4	0.728	3.915	3.539	3.617	2.726	1.475	0.632	88	91	63	24	-13	2.11E-6	4.37E-5	> 1.00E-4
RPMI-8226	4.586	10.694	11.107	10.272	9.928	5.236	3.148	107	93	87	11	-31	3.07E-6	1.79E-5	> 1.00E-4
SR	0.953	6.344	6.172	5.812	5.615	3.149	1.158	97	90	86	41	4	6.28E-6	> 1.00E-4	> 1.00E-4
Non-Small Cell Lung Cancer															
A549/ATCC	0.392	3.908	3.604	3.649	3.650	1.998	0.648	91	93	93	46	7	8.08E-6	> 1.00E-4	> 1.00E-4
EKVX	5.120	12.856	12.192	11.639	11.860	9.247	6.130	91	84	87	53	13	1.21E-5	> 1.00E-4	> 1.00E-4
HOP-62	1.512	4.540	4.735	4.523	4.152	2.581	0.454	106	99	87	35	-70	5.21E-6	2.16E-5	6.46E-5
HOP-92	8.037	10.362	10.442	10.335	9.564	5.660	0.436	104	99	66	-30	-95	1.46E-6	4.89E-6	2.06E-5
NCI-H226	6.623	16.738	15.580	15.267	15.202	9.744	0.938	88	85	85	31	-86	4.42E-6	1.84E-5	4.93E-5
NCI-H23	4.634	12.756	12.341	12.497	11.754	9.688	4.887	95	97	88	62	3	1.61E-5	> 1.00E-4	> 1.00E-4
NCI-H322M	6.734	14.344	14.022	13.725	13.139	10.570	7.019	96	92	84	50	4	1.02E-5	> 1.00E-4	> 1.00E-4
NCI-H460	0.969	14.460	13.629	14.710	11.745	9.895	6.033	94	102	80	66	38	3.68E-5	> 1.00E-4	> 1.00E-4
NCI-H522	3.316	8.861	8.637	8.561	8.894	6.522	2.673	96	94	101	58	-19	1.26E-5	5.61E-5	> 1.00E-4
Colon Cancer															
COLO 205	0.835	4.041	4.424	4.046	3.540	2.016	1.149	112	100	84	37	10	5.29E-6	> 1.00E-4	> 1.00E-4
HCC-2998	4.833	13.588	12.909	11.964	11.461	9.942	5.656	92	82	76	58	9	1.47E-5	> 1.00E-4	> 1.00E-4
HCT-116	0.373	4.693	4.548	4.175	3.171	2.081	1.132	97	88	65	40	18	3.83E-6	> 1.00E-4	> 1.00E-4
HCT-15	1.263	11.913	11.887	10.258	10.063	8.968	8.472	100	84	83	74	68	> 1.00E-4	> 1.00E-4	> 1.00E-4
HT29	0.509	4.912	4.698	4.687	4.321	2.669	1.707	95	95	87	49	27	9.43E-6	> 1.00E-4	> 1.00E-4
KM12	0.587	3.873	3.656	3.670	3.163	2.217	1.587	93	94	78	50	30	9.70E-6	> 1.00E-4	> 1.00E-4
SW-620	0.580	4.852	4.706	4.830	3.974	2.344	1.324	97	99	80	41	17	5.91E-6	> 1.00E-4	> 1.00E-4
CNS Cancer															
SF-268	1.084	3.232	2.993	3.076	2.865	1.812	0.726	89	93	83	34	-33	4.69E-6	3.21E-5	> 1.00E-4
SF-295	1.714	4.332	4.422	4.184	4.070	3.184	0.549	103	94	90	56	-68	1.12E-5	2.83E-5	7.17E-5
SF-539	4.105	12.554	12.171	12.134	11.853	9.887	4.063	96	95	92	68	-4	1.80E-5	8.89E-5	> 1.00E-4
SNB-19	1.783	4.810	4.950	4.796	4.693	4.065	2.477	105	100	96	75	23	3.05E-5	> 1.00E-4	> 1.00E-4
SNB-75	2.056	3.045	2.982	2.838	2.702	0.884	0.054	94	79	65	-57	-97	1.34E-6	3.42E-6	8.76E-6
U251	0.623	3.611	3.745	3.600	3.253	1.422	0.329	104	100	88	27	-47	4.17E-6	2.30E-5	> 1.00E-4
Melanoma															
LOX IMVI	0.362	2.388	2.643	2.686	2.034	0.789	0.154	113	115	82	21	-58	3.38E-6	1.85E-5	8.04E-5
MALME-3M	2.638	4.344	4.211	3.850	3.862	2.658	0.440	93	70	72	3	-83	2.07E-6	1.07E-5	4.10E-5
M14	4.190	12.645	12.912	12.555	12.364	7.776	3.783	103	99	97	42	-10	7.24E-6	6.51E-5	> 1.00E-4
MDA-MB-435	1.129	3.754	3.671	3.766	3.584	3.128	1.958	97	100	94	76	32	3.86E-5	> 1.00E-4	> 1.00E-4
SK-MEL-2	2.588	5.976	5.959	5.977	5.940	3.021	0.513	100	100	99	13	-80	3.70E-6	1.37E-5	4.74E-5
SK-MEL-28	1.628	5.149	5.185	5.021	5.159	4.917	0.315	101	96	100	93	-81	1.78E-5	3.44E-5	6.67E-5
SK-MEL-5	2.807	8.771	8.676	7.225	7.911	6.855	3.896	98	74	85	68	18	2.29E-5	> 1.00E-4	> 1.00E-4
UACC-257	4.071	9.092	8.546	8.501	8.714	7.639	4.520	89	88	93	71	9	2.18E-5	> 1.00E-4	> 1.00E-4
UACC-62	0.765	3.448	3.348	3.352	3.251	2.823	1.413	96	96	93	77	24	3.22E-5	> 1.00E-4	> 1.00E-4
Ovarian Cancer															
IGROV1	1.599	4.239	3.995	4.146	3.999	2.848	1.186	91	96	91	47	-26	8.67E-6	4.43E-5	> 1.00E-4
OVCAR-3	2.879	11.483	9.897	10.072	10.051	5.784	1.266	82	84	83	34	-56	4.72E-6	2.38E-5	8.57E-5
OVCAR-4	4.638	8.145	8.357	7.861	5.512	0.707	0.105	106	92	25	-85	-98	4.22E-7	1.69E-6	4.82E-6
OVCAR-5	5.432	11.759	11.623	11.388	11.356	9.517	5.730	98	94	94	65	5	1.75E-5	> 1.00E-4	> 1.00E-4
OVCAR-8	0.988	4.758	4.681	4.607	4.529	3.290	0.896	98	96	94	61	-10	1.43E-5	7.33E-5	> 1.00E-4
NCI/ADR-RES	3.228	11.002	10.759	10.703	10.301	8.026	3.644	97	96	91	62	5	1.62E-5	> 1.00E-4	> 1.00E-4
SK-OV-3	2.770	12.794	12.058	12.172	12.213	6.715	1.813	93	94	94	39	-34	6.41E-6	3.41E-5	> 1.00E-4
Renal Cancer															
786-0	1.731	.	.	.	.	.	.	.	.	.	.	.	.	.	.
A498	2.609	8.743	9.010	8.468	7.752	3.394	0.682	104	95	84	13	-74	2.99E-6	1.40E-5	5.30E-5
ACHN	1.003	4.360	4.202	4.060	3.984	2.210	0.431	95	91	88	36	-57	5.36E-6	2.43E-5	8.39E-5
CAKI-1	1.015	4.749	4.663	4.575	4.612	2.840	0.659	98	95	96	49	-35	9.48E-6	3.82E-5	> 1.00E-4
RXF 393	2.078	3.544	3.425	3.559	3.559	2.248	0.178	92	101	101	12	-91	3.72E-6	1.29E-5	3.96E-5
SN12C	0.660	2.686	2.608	2.683	2.657	1.679	0.679	96	100	99	50	1	1.01E-5	> 1.00E-4	> 1.00E-4
TK-10	7.459	17.461	16.528	17.176	16.579	9.550	2.514	91	97	91	21	-66	3.85E-6	1.73E-5	6.50E-5
UO-31	1.590	6.064	5.856	5.872	5.949	4.774	1.563	95	96	97	71	-2	1.95E-5	9.48E-5	> 1.00E-4
Prostate Cancer															
PC-3	4.255	11.426	10.593	10.665	8.797	6.295	2.768	88	89	63	28	-35	2.40E-6	2.81E-5	> 1.00E-4
DU-145	0.789	3.473	3.608	3.677	3.096	2.252	1.276	105	108	86	54	18	1.33E-5	> 1.00E-4	> 1.00E-4
Breast Cancer															
MCF7	0.974	8.476	8.038	8.393	6.964	5.292	2.836	94	99	80	58	25	1.72E-5	> 1.00E-4	> 1.00E-4
MDA-MB-231/ATCC	4.073	9.051	9.111	9.147	8.265	4.026	0.279	101	102	84	-1	-93	2.50E-6	9.63E-6	3.39E-5
HS 578T	2.251	4.094	4.040	4.127	3.860	1.601	0.072	97	102	87	-29	-97	2.10E-6	5.64E-6	2.05E-5
BT-549	6.928	12.569	12.067	12.355	12.379	8.935	2.656	91	96	97	36	-62	5.81E-6	2.32E-5	7.59E-5
T-47D	6.839	11.355	10.884	10.524	10.738	8.818	7.511	89	82	86	44	15	7.13E-6	> 1.00E-4	> 1.00E-4
MDA-MB-468	5.295	9.590	9.664	9.354	9.027	5.363	2.362	102	94	87	2	-55	2.70E-6	1.07E-5	8.04E-5

Figure S17. Anticancer activity (five-dose assay) of the azatetracyclic derivative **3l**.

Table S1. CLC-Pred data of the azaheterocyclic derivative **3a**.

Pa	Pi	Cell-line	Description	Tissue/Organ	Type	IAP*
0.644	0.005	SW48	Colorectal Adenocarcinoma	Colon	Adenocarcinoma	0.806
0.559	0.130	A2780cisR	Cisplatin-resistant ovarian carcinoma	Ovarium	Carcinoma	0.838
0.529	0.035	SK-LU-1	Adenocarcinoma	Lung	Carcinoma	0.810
0.462	0.020	UMUC3	Bladder Carcinoma	Urinary tract	Carcinoma	0.810
0.460	0.107	MCF7	Breast carcinoma	Breast	Carcinoma	0.836
0.456	0.013	HEL299	Fibroblasts	Lung	Normal	0.899
0.444	0.050	HCC1937	Breast Carcinoma	Breast	Carcinoma	0.806
0.433	0.016	SK-MES-1	Squamous cell lung carcinoma	Lung	Carcinoma	0.834
0.422	0.089	DU-4475	Breast Carcinoma	Breast	Carcinoma	0.802
0.412	0.021	RD	Rhabdomyosarcoma	Muscle	Normal	0.828
0.410	0.055	SNU-5	Gastric Carcinoma	Stomach	Carcinoma	0.828
0.408	0.165	TMD8	Diffuse large B-cell lymphoma activated B-cell type	Lymphocytes	Lymphoma	0.811
0.399	0.028	NCI-H292	Mucoepidermoid Pulmonary Carcinoma	Lung	Carcinoma	0.845
0.397	0.024	HuP-T3	Pancreatic adenocarcinoma	Pancreas	Adenocarcinoma	0.868
0.391	0.040	COR-L23	Lung large cell carcinoma	Lung	Carcinoma	0.834
0.389	0.003	SW1116	Colorectal Adenocarcinoma	Colon	Adenocarcinoma	0.864
0.384	0.045	EOL1	Chronic eosinophilic leukemia, not otherwise specified	Blood	Leukemia	0.861
0.383	0.062	OE33	Barrett adenocarcinoma	Esophagus	Adenocarcinoma	0.805
0.377	0.100	NCI-H358	Bronchioalveolar Carcinoma	Lung; Bronchiole	Carcinoma	0.805
0.368	0.008	CFPAC-1	Pancreatic carcinoma	Pancreas	Carcinoma	0.905
0.367	0.055	KYSE-520	Esophageal squamous cell carcinoma	Esophagus	Carcinoma	0.858
0.363	0.109	Kasumi-1	Acute myeloblastic leukemia	Blood	Leukemia	0.816
0.360	0.022	PA-1	Ovarian carcinoma	Ovarium	Carcinoma	0.854
0.357	0.044	HCC1806	Acantholytic Squamous Cell Carcinoma	Breast	Carcinoma	0.824
0.352	0.137	SK-MEL-1	Metastatic melanoma	Skin	Melanoma	0.808
0.344	0.124	A172	Glioblastoma	Brain	Glioblastoma	0.807
0.340	0.097	RS4-11	Adult B acute lymphoblastic leukemia	Bone Marrow	Leukemia	0.835
0.340	0.126	CAL-51	Breast carcinoma	Breast	Carcinoma	0.824
0.339	0.122	HT1197	Carcinoma	Urinary bladder	Carcinoma	0.815
0.339	0.179	NCI-H441	Papillary adenocarcinoma	Lung	Adenocarcinoma	0.813
0.338	0.025	MSTO-211H	Biphasic Mesothelioma	Lung	Mesothelioma	0.878
0.338	0.231	OCI-AML2	Adult acute myeloid leukemia	Blood	Leukemia	0.803
0.330	0.039	NCI-H727	Carcinoid	Lung; Bronchus	Carcinoma	0.828
0.317	0.063	NCI-H520	Squamous Cell Carcinoma	Lung	Carcinoma	0.837
0.311	0.057	SR	Adult immunoblastic lymphoma	Haematopoietic and lymphoid tissue	Lymphoma	0.877
0.308	0.103	HCC1954	Breast Carcinoma	Breast	Carcinoma	0.829
0.306	0.185	GIST430	Gastrointestinal stromal tumor	Intestine	Carcinoma	0.820
0.298	0.092	RPMI-7951	Malignant Melanoma	Skin	Melanoma	0.854
0.297	0.054	CAL-27	Squamous Cell Carcinoma	Tongue	Carcinoma	0.854

Pa	Pi	Cell-line	Description	Tissue/Organ	Type	IAP*
0.297	0.080	HOS	Osteosarcoma	Bone	Sarcoma	0.863
0.291	0.058	NCI-H1581	Non Small Cell Lung Cancer	Lung	Carcinoma	0.871
0.291	0.140	NCI-H661	Lung carcinoma	Lung	Carcinoma	0.831
0.286	0.220	CAKI-2	Kidney carcinoma	Kidney	Carcinoma	0.829
0.283	0.061	AGS	Gastric adenocarcinoma	Stomach	Adenocarcinoma	0.875
0.272	0.101	A2058	Melanoma	Skin	Melanoma	0.859
0.268	0.144	8505C	Thyroid gland undifferentiated (anaplastic) carcinoma	Thyroid	Carcinoma	0.832
0.268	0.158	NCI-H1650	Bronchoalveolar carcinoma	Lung	Adenocarcinoma	0.839
0.266	0.225	DMS-114	Lung carcinoma	Lung	Carcinoma	0.834
0.263	0.124	H9	T-lymphoid	Haematopoietic and lymphoid tissue	Leukemia	0.872
0.256	0.109	U-266	Plasma cell myeloma	Blood	Myeloma	0.870
0.255	0.011	T98G	Glioblastoma	Brain	Carcinoma	0.912
0.253	0.120	OVCAR-5	Ovarian adenocarcinoma	Ovarium	Adenocarcinoma	0.866
0.243	0.169	MKN-74	Gastric tubular adenocarcinoma	Stomach	Adenocarcinoma	0.856
0.232	0.126	SK-OV-3	Ovarian carcinoma	Ovarium	Carcinoma	0.896
0.227	0.126	A-427	Lung carcinoma	Lung	Carcinoma	0.872
0.225	0.110	MRC5	Embryonic lung fibroblast	Lung	Normal	0.916
0.221	0.138	RCC4	Clear cell renal cell carcinoma	Kidney	Carcinoma	0.845
0.213	0.062	SW480	Colon adenocarcinoma	Colon	Adenocarcinoma	0.932
0.213	0.121	U2OS	Osteosarcoma	Bone	Sarcoma	0.885
0.211	0.192	MDA-MB-361	Breast adenocarcinoma	Breast	Adenocarcinoma	0.872
0.209	0.159	SW-620	Colon adenocarcinoma	Colon	Adenocarcinoma	0.887
0.201	0.008	NCI-H2228	Non-small cell lung cancer	Lung	Adenocarcinoma	0.920
0.200	0.163	CAPAN-1	Pancreas Adenocarcinoma	Pancreas	Adenocarcinoma	0.889
0.200	0.174	RKO	Colon carcinoma	Colon	Carcinoma	0.879
0.199	0.015	IMR-32	Neuroblastoma	Nervous system	Neuroblastoma	0.951
0.198	0.079	SUM149PT	Breast inflammatory carcinoma	Breast	Carcinoma	0.983
0.197	0.114	OCI-Ly10	Diffuse large B-cell lymphoma activated B-cell type	Lymphoid tissue	Lymphoma	0.919
0.193	0.064	HT-1080	Fibrosarcoma	Soft tissue	Sarcoma	0.918
0.184	0.108	SK-HEP1	Hepatocellular carcinoma	Liver	Carcinoma	0.912
0.179	0.167	HOP-18	Non-small cell lung carcinoma	Lung	Carcinoma	0.933
0.176	0.143	KG-1	Acute myelogenous leukemia	Blood	Leukemia	0.901
0.168	0.011	SJSA-1	Osteosarcoma	Bone	Sarcoma	0.940
0.160	0.008	KARPAS-299	Anaplastic large cell lymphoma	Haematopoietic and lymphoid tissue	Leukemia	0.939
0.156	0.058	MV4-11	Myeloid leukemia	Haematopoietic and lymphoid tissue	Leukemia	0.947
0.155	0.037	SKM-1	Adult acute myeloid leukemia	Blood	Leukemia	0.917
0.132	0.116	A-431	Epidermoid carcinoma	Skin	Carcinoma	0.937
0.126	0.094	DLD-1	Colon adenocarcinoma	Colon	Adenocarcinoma	0.949
0.102	0.100	WI-38	Embryonic lung fibroblast	Lung	Normal	0.943
0.100	0.029	BEAS-2B	Epithelial cells	bronchial epithelium	Normal	1.000
0.084	0.064	MOLM-13	Adult acute myeloid leukemia	Blood	Leukemia	0.967
0.064	0.032	NAMALVA	EBV-related Burkitt lymphoma	Blood	Lymphoma	0.986

0.049	0.010	M21	Melanoma	Skin	Melanoma	0.985
0.038	0.005	JY	EBV-positive lymphoblastoid B cell line	Blood	Normal	1.000
0.013	0.008	QG-56	Squamous cell lung carcinoma	Lung	Carcinoma	1.000

Table S2. CLC-Pred data of the azaheterocyclic derivative **3b**.

Pa	Pi	Cell-line	Description	Tissue/Organ	Type	IAP*
0.597	0.005	SW48	Colorectal Adenocarcinoma	Colon	Adenocarcinoma	0.806
0.511	0.047	SK-LU-1	Adenocarcinoma	Lung	Carcinoma	0.810
0.466	0.019	UMUC3	Bladder Carcinoma	Urinary tract	Carcinoma	0.810
0.446	0.049	HCC1937	Breast Carcinoma	Breast	Carcinoma	0.806
0.444	0.030	SNU-5	Gastric Carcinoma	Stomach	Carcinoma	0.828
0.426	0.019	SK-MES-1	Squamous cell lung carcinoma	Lung	Carcinoma	0.834
0.421	0.090	DU-4475	Breast Carcinoma	Breast	Carcinoma	0.802
0.412	0.161	TMD8	Diffuse large B-cell lymphoma activated B-cell type	Lymphocytes	Lymphoma	0.811
0.411	0.020	HEL299	Fibroblasts	Lung	Normal	0.899
0.397	0.024	HuP-T3	Pancreatic adenocarcinoma	Pancreas	Adenocarcinoma	0.868
0.397	0.048	OE33	Barrett adenocarcinoma	Esophagus	Adenocarcinoma	0.805
0.395	0.143	MCF7	Breast carcinoma	Breast	Carcinoma	0.836
0.392	0.027	RD	Rhabdomyosarcoma	Muscle	Normal	0.828
0.385	0.045	COR-L23	Lung large cell carcinoma	Lung	Carcinoma	0.834
0.382	0.038	NCI-H292	Mucoepidermoid Pulmonary Carcinoma	Lung	Carcinoma	0.845
0.372	0.004	SW1116	Colorectal Adenocarcinoma	Colon	Adenocarcinoma	0.864
0.372	0.201	GIST882	Gastrointestinal stromal tumor	Intestine	Carcinoma	0.824
0.364	0.114	GIST430	Gastrointestinal stromal tumor	Intestine	Carcinoma	0.820
0.363	0.008	CFPAC-1	Pancreatic carcinoma	Pancreas	Carcinoma	0.905
0.363	0.116	NCI-H358	Bronchioalveolar Carcinoma	Lung; Bronchiole	Carcinoma	0.805
0.362	0.111	Kasumi-1	Acute myeloblastic leukemia	Blood	Leukemia	0.816
0.362	0.140	NCI-H441	Papillary adenocarcinoma	Lung	Adenocarcinoma	0.813
0.358	0.069	EOL1	Chronic eosinophilic leukemia, not otherwise specified	Blood	Leukemia	0.861
0.357	0.068	KYSE-520	Esophageal squamous cell carcinoma	Esophagus	Carcinoma	0.858
0.354	0.047	HCC1806	Acantholytic Squamous Cell Carcinoma	Breast	Carcinoma	0.824
0.348	0.029	PA-1	Ovarian carcinoma	Ovarium	Carcinoma	0.854
0.347	0.087	RS4-11	Adult B acute lymphoblastic leukemia	Bone Marrow	Leukemia	0.835
0.346	0.252	A2780cisR	Cisplatin-resistant ovarian carcinoma	Ovarium	Carcinoma	0.838
0.339	0.158	SK-MEL-1	Metastatic melanoma	Skin	Melanoma	0.808
0.338	0.231	OCI-AML2	Adult acute myeloid leukemia	Blood	Leukemia	0.803
0.334	0.036	NCI-H727	Carcinoid	Lung; Bronchus	Carcinoma	0.828
0.330	0.138	HT1197	Carcinoma	Urinary bladder	Carcinoma	0.815
0.325	0.160	CAL-51	Breast carcinoma	Breast	Carcinoma	0.824
0.322	0.275	GIST48	Gastrointestinal stromal tumor	Intestine	Carcinoma	0.805

0.319	0.040	MSTO-211H	Biphasic Mesothelioma	Lung	Mesothelioma	0.878
0.316	0.202	A172	Glioblastoma	Brain	Glioblastoma	0.807
0.313	0.070	NCI-H520	Squamous Cell Carcinoma	Lung	Carcinoma	0.837
0.306	0.076	NCI-H1650	Bronchoalveolar carcinoma	Lung	Adenocarcinoma	0.839
0.305	0.046	CAL-27	Squamous Cell Carcinoma	Tongue	Carcinoma	0.854
0.300	0.128	HCC1954	Breast Carcinoma	Breast	Carcinoma	0.829
0.297	0.079	HOS	Osteosarcoma	Bone	Sarcoma	0.863
0.295	0.023	SUM149PT	Breast inflammatory carcinoma	Breast	Carcinoma	0.983
0.294	0.099	RPMI-7951	Malignant Melanoma	Skin	Melanoma	0.854
0.291	0.086	A2058	Melanoma	Skin	Melanoma	0.859
0.283	0.163	NCI-H661	Lung carcinoma	Lung	Carcinoma	0.831
0.280	0.075	NCI-H1581	Non Small Cell Lung Cancer	Lung	Carcinoma	0.871
0.271	0.085	SR	Adult immunoblastic lymphoma	Haematopoietic and lymphoid tissue	Lymphoma	0.877
0.270	0.114	H9	T-lymphoid	Haematopoietic and lymphoid tissue	Leukemia	0.872
0.262	0.093	AGS	Gastric adenocarcinoma	Stomach	Adenocarcinoma	0.875
0.261	0.010	T98G	Glioblastoma	Brain	Carcinoma	0.912
0.258	0.170	8505C	Thyroid gland undifferentiated (anaplastic) carcinoma	Thyroid	Carcinoma	0.832
0.239	0.134	U-266	Plasma cell myeloma	Blood	Myeloma	0.870
0.235	0.084	CAPAN-1	Pancreas Adenocarcinoma	Pancreas	Adenocarcinoma	0.889
0.227	0.159	OVCAR-5	Ovarian adenocarcinoma	Ovarium	Adenocarcinoma	0.866
0.222	0.058	SW480	Colon adenocarcinoma	Colon	Adenocarcinoma	0.932
0.212	0.134	RKO	Colon carcinoma	Colon	Carcinoma	0.879
0.212	0.187	MDA-MB-361	Breast adenocarcinoma	Breast	Adenocarcinoma	0.872
0.210	0.171	A-427	Lung carcinoma	Lung	Carcinoma	0.872
0.209	0.128	U2OS	Osteosarcoma	Bone	Sarcoma	0.885
0.198	0.092	KG-1	Acute myelogenous leukemia	Blood	Leukemia	0.901
0.196	0.013	NCI-H2228	Non-small cell lung cancer	Lung	Adenocarcinoma	0.920
0.195	0.145	MRC5	Embryonic lung fibroblast	Lung	Normal	0.916
0.194	0.093	SK-HEP1	Hepatocellular carcinoma	Liver	Carcinoma	0.912
0.182	0.020	IMR-32	Neuroblastoma	Nervous system	Neuroblastoma	0.951
0.168	0.095	HT-1080	Fibrosarcoma	Soft tissue	Sarcoma	0.918
0.167	0.012	SJSA-1	Osteosarcoma	Bone	Sarcoma	0.940
0.166	0.083	U-87MG	Glioblastoma	Brain	Blastoma	0.921
0.159	0.029	SKM-1	Adult acute myeloid leukemia	Blood	Leukemia	0.917
0.157	0.128	NCI-N87	Gastric carcinoma	Stomach	Carcinoma	0.923
0.153	0.061	MV4-11	Myeloid leukemia	Haematopoietic and lymphoid tissue	Leukemia	0.947
0.151	0.011	KARPAS-299	Anaplastic large cell lymphoma	Haematopoietic and lymphoid tissue	Leukemia	0.939
0.145	0.095	A-431	Epidermoid carcinoma	Skin	Carcinoma	0.937
0.142	0.069	Jurkat	Acute leukemic T-cells	Blood	Leukemia	0.946
0.132	0.119	THP-1	Acute monocytic leukemia	Blood	Leukemia	0.937
0.126	0.094	DLD-1	Colon adenocarcinoma	Colon	Adenocarcinoma	0.949
0.117	0.104	RWPE-1	Prostatic epithelial cell line	Prostate	Normal	0.922
0.104	0.091	Ramos	Burkitts lymhoma B-cells	Blood	Leukemia	0.958
0.103	0.032	MOLM-13	Adult acute myeloid leukemia	Blood	Leukemia	0.967

0.102	0.099	WI-38	Embryonic lung fibroblast	Lung	Normal	0.943
0.068	0.058	KBM5	Chronic myelogenous leukemia, BCR-ABL1 positive	Blood	Leukemia	0.931
0.062	0.037	NAMALVA	EBV-related Burkitt lymphoma	Blood	Lymphoma	0.986
0.047	0.013	M21	Melanoma	Skin	Melanoma	0.985

Table S3. CLC-Pred data of the azaheterocyclic derivative **3c**.

Pa	Pi	Cell-line	Description	Tissue/Organ	Type	IAP*
0.572	0.007	SW48	Colorectal Adenocarcinoma	Colon	Adenocarcinoma	0.806
0.502	0.054	SK-LU-1	Adenocarcinoma	Lung	Carcinoma	0.810
0.447	0.015	HEL299	Fibroblasts	Lung	Normal	0.899
0.440	0.054	HCC1937	Breast Carcinoma	Breast	Carcinoma	0.806
0.437	0.032	UMUC3	Bladder Carcinoma	Urinary tract	Carcinoma	0.810
0.412	0.028	SK-MES-1	Squamous cell lung carcinoma	Lung	Carcinoma	0.834
0.412	0.099	DU-4475	Breast Carcinoma	Breast	Carcinoma	0.802
0.389	0.075	SNU-5	Gastric Carcinoma	Stomach	Carcinoma	0.828
0.383	0.038	NCI-H292	Mucoepidermoid Pulmonary Carcinoma	Lung	Carcinoma	0.845
0.381	0.047	EOL1	Chronic eosinophilic leukemia, not otherwise specified	Blood	Leukemia	0.861
0.379	0.004	SW1116	Colorectal Adenocarcinoma	Colon	Adenocarcinoma	0.864
0.373	0.035	RD	Rhabdomyosarcoma	Muscle	Normal	0.828
0.370	0.036	HuP-T3	Pancreatic adenocarcinoma	Pancreas	Adenocarcinoma	0.868
0.368	0.161	MCF7	Breast carcinoma	Breast	Carcinoma	0.836
0.367	0.055	KYSE-520	Esophageal squamous cell carcinoma	Esophagus	Carcinoma	0.858
0.364	0.115	NCI-H358	Bronchioalveolar Carcinoma	Lung; Bronchiole	Carcinoma	0.805
0.362	0.072	COR-L23	Lung large cell carcinoma	Lung	Carcinoma	0.834
0.353	0.047	HCC1806	Acantholytic Squamous Cell Carcinoma	Breast	Carcinoma	0.824
0.351	0.009	CFPAC-1	Pancreatic carcinoma	Pancreas	Carcinoma	0.905
0.346	0.020	MSTO-211H	Biphasic Mesothelioma	Lung	Mesothelioma	0.878
0.346	0.252	A2780cisR	Cisplatin-resistant ovarian carcinoma	Ovarium	Carcinoma	0.838
0.341	0.033	PA-1	Ovarian carcinoma	Ovarium	Carcinoma	0.854
0.340	0.123	OE33	Barrett adenocarcinoma	Esophagus	Adenocarcinoma	0.805
0.339	0.098	RS4-11	Adult B acute lymphoblastic leukemia	Bone Marrow	Leukemia	0.835
0.324	0.050	SR	Adult immunoblastic lymphoma	Haematopoietic and lymphoid tissue	Lymphoma	0.877
0.321	0.170	CAL-51	Breast carcinoma	Breast	Carcinoma	0.824
0.318	0.200	SK-MEL-1	Metastatic melanoma	Skin	Melanoma	0.808
0.313	0.209	A172	Glioblastoma	Brain	Glioblastoma	0.807
0.311	0.064	NCI-H727	Carcinoid	Lung; Bronchus	Carcinoma	0.828
0.310	0.177	HT1197	Carcinoma	Urinary bladder	Carcinoma	0.815
0.310	0.184	Kasumi-1	Acute myeloblastic leukemia	Blood	Leukemia	0.816
0.309	0.075	NCI-H520	Squamous Cell Carcinoma	Lung	Carcinoma	0.837
0.306	0.237	NCI-H441	Papillary adenocarcinoma	Lung	Adenocarcinoma	0.813
0.300	0.276	OCI-AML2	Adult acute myeloid leukemia	Blood	Leukemia	0.803

0.299	0.046	NCI-H1581	Non Small Cell Lung Cancer	Lung	Carcinoma	0.871
0.292	0.085	HOS	Osteosarcoma	Bone	Sarcoma	0.863
0.292	0.154	HCC1954	Breast Carcinoma	Breast	Carcinoma	0.829
0.288	0.067	CAL-27	Squamous Cell Carcinoma	Tongue	Carcinoma	0.854
0.280	0.171	NCI-H661	Lung carcinoma	Lung	Carcinoma	0.831
0.272	0.146	NCI-H1650	Bronchoalveolar carcinoma	Lung	Adenocarcinoma	0.839
0.272	0.258	CAKI-2	Kidney carcinoma	Kidney	Carcinoma	0.829
0.268	0.253	OS-RC-2	Clear cell renal cell carcinoma	Kidney	Carcinoma	0.812
0.266	0.147	RPMI-7951	Malignant Melanoma	Skin	Melanoma	0.854
0.264	0.090	AGS	Gastric adenocarcinoma	Stomach	Adenocarcinoma	0.875
0.258	0.169	8505C	Thyroid gland undifferentiated (anaplastic) carcinoma	Thyroid	Carcinoma	0.832
0.258	0.190	EKVX	Non-small cell lung carcinoma	Lung	Carcinoma	0.841
0.257	0.115	OVCAR-5	Ovarian adenocarcinoma	Ovarium	Adenocarcinoma	0.866
0.253	0.151	MKN-74	Gastric tubular adenocarcinoma	Stomach	Adenocarcinoma	0.856
0.251	0.115	U-266	Plasma cell myeloma	Blood	Myeloma	0.870
0.246	0.130	A2058	Melanoma	Skin	Melanoma	0.859
0.242	0.014	T98G	Glioblastoma	Brain	Carcinoma	0.912
0.240	0.160	H9	T-lymphoid	Haematopoietic and lymphoid tissue	Leukemia	0.872
0.227	0.136	SW-620	Colon adenocarcinoma	Colon	Adenocarcinoma	0.887
0.224	0.203	Hs-578T	Invasive ductal breast carcinoma	Breast	Carcinoma	0.869
0.223	0.194	UACC-257	Melanoma	Skin	Melanoma	0.861
0.221	0.115	MRC5	Embryonic lung fibroblast	Lung	Normal	0.916
0.219	0.200	TK-10	Renal carcinoma	Kidney	Carcinoma	0.854
0.215	0.069	SK-HEP1	Hepatocellular carcinoma	Liver	Carcinoma	0.912
0.215	0.157	A-427	Lung carcinoma	Lung	Carcinoma	0.872
0.213	0.120	U2OS	Osteosarcoma	Bone	Sarcoma	0.885
0.212	0.136	RKO	Colon carcinoma	Colon	Carcinoma	0.879
0.201	0.009	NCI-H2228	Non-small cell lung cancer	Lung	Adenocarcinoma	0.920
0.191	0.106	KG-1	Acute myelogenous leukemia	Blood	Leukemia	0.901
0.184	0.019	IMR-32	Neuroblastoma	Nervous system	Neuroblastoma	0.951
0.179	0.005	SJSA-1	Osteosarcoma	Bone	Sarcoma	0.940
0.175	0.083	HT-1080	Fibrosarcoma	Soft tissue	Sarcoma	0.918
0.166	0.086	SW480	Colon adenocarcinoma	Colon	Adenocarcinoma	0.932
0.162	0.140	ASPC1	Pancreatic ductal adenocarcinoma	Pancreas	Adenocarcinoma	0.901
0.158	0.008	KARPAS-299	Anaplastic large cell lymphoma	Haematopoietic and lymphoid tissue	Leukemia	0.939
0.149	0.052	SKM-1	Adult acute myeloid leukemia	Blood	Leukemia	0.917
0.146	0.065	Jurkat	Acute leukemic T-cells	Blood	Leukemia	0.946
0.144	0.071	MV4-11	Myeloid leukemia	Haematopoietic and lymphoid tissue	Leukemia	0.947
0.132	0.115	A-431	Epidermoid carcinoma	Skin	Carcinoma	0.937
0.120	0.103	DLD-1	Colon adenocarcinoma	Colon	Adenocarcinoma	0.949
0.087	0.071	HA22T	Hepatocellular carcinoma	Liver	Carcinoma	0.946
0.060	0.042	NAMALVA	EBV-related Burkitt lymphoma	Blood	Lymphoma	0.986
0.047	0.012	M21	Melanoma	Skin	Melanoma	0.985
0.033	0.008	JY	EBV-positive lymphoblastoid B cell line	Blood	Normal	1.000

Table S4. CLC-Pred data of the azaheterocyclic derivative **3d**.

Pa	Pi	Cell-line	Description	Tissue/Organ	Type	IAP*
0.577	0.006	SW48	Colorectal Adenocarcinoma	Colon	Adenocarcinoma	0.806
0.549	0.068	MCF7	Breast carcinoma	Breast	Carcinoma	0.836
0.487	0.068	SK-LU-1	Adenocarcinoma	Lung	Carcinoma	0.810
0.424	0.040	UMUC3	Bladder Carcinoma	Urinary tract	Carcinoma	0.810
0.413	0.027	SK-MES-1	Squamous cell lung carcinoma	Lung	Carcinoma	0.834
0.411	0.020	HEL299	Fibroblasts	Lung	Normal	0.899
0.386	0.029	RD	Rhabdomyosarcoma	Muscle	Normal	0.828
0.381	0.031	HuP-T3	Pancreatic adenocarcinoma	Pancreas	Adenocarcinoma	0.868
0.381	0.039	NCI-H292	Mucoepidermoid Pulmonary Carcinoma	Lung	Carcinoma	0.845
0.380	0.139	DU-4475	Breast Carcinoma	Breast	Carcinoma	0.802
0.377	0.088	SNU-5	Gastric Carcinoma	Stomach	Carcinoma	0.828
0.369	0.004	SW1116	Colorectal Adenocarcinoma	Colon	Adenocarcinoma	0.864
0.366	0.020	PA-1	Ovarian carcinoma	Ovarium	Carcinoma	0.854
0.354	0.133	SK-MEL-1	Metastatic melanoma	Skin	Melanoma	0.808
0.351	0.089	COR-L23	Lung large cell carcinoma	Lung	Carcinoma	0.834
0.349	0.174	HCC1937	Breast Carcinoma	Breast	Carcinoma	0.806
0.346	0.252	A2780cisR	Cisplatin-resistant ovarian carcinoma	Ovarium	Carcinoma	0.838
0.341	0.030	NCI-H727	Carcinoid	Lung; Bronchus	Carcinoma	0.828
0.340	0.009	CFPAC-1	Pancreatic carcinoma	Pancreas	Carcinoma	0.905
0.340	0.090	EOL1	Chronic eosinophilic leukemia, not otherwise specified	Blood	Leukemia	0.861
0.334	0.028	MSTO-211H	Biphasic Mesothelioma	Lung	Mesothelioma	0.878
0.317	0.104	HCC1806	Acantholytic Squamous Cell Carcinoma	Breast	Carcinoma	0.824
0.309	0.075	NCI-H520	Squamous Cell Carcinoma	Lung	Carcinoma	0.837
0.307	0.183	HT1197	Carcinoma	Urinary bladder	Carcinoma	0.815
0.306	0.150	KYSE-520	Esophageal squamous cell carcinoma	Esophagus	Carcinoma	0.858
0.302	0.154	RS4-11	Adult B acute lymphoblastic leukemia	Bone Marrow	Leukemia	0.835
0.302	0.185	OE33	Barrett adenocarcinoma	Esophagus	Adenocarcinoma	0.805
0.298	0.253	NCI-H441	Papillary adenocarcinoma	Lung	Adenocarcinoma	0.813
0.290	0.210	Kasumi-1	Acute myeloblastic leukemia	Blood	Leukemia	0.816
0.280	0.081	CAL-27	Squamous Cell Carcinoma	Tongue	Carcinoma	0.854
0.272	0.115	HOS	Osteosarcoma	Bone	Sarcoma	0.863
0.262	0.158	RPMI-7951	Malignant Melanoma	Skin	Melanoma	0.854
0.259	0.130	H9	T-lymphoid	Haematopoietic and lymphoid tissue	Leukemia	0.872
0.255	0.119	A2058	Melanoma	Skin	Melanoma	0.859
0.254	0.253	NCI-H661	Lung carcinoma	Lung	Carcinoma	0.831
0.253	0.118	NCI-H1581	Non Small Cell Lung Cancer	Lung	Carcinoma	0.871
0.250	0.194	8505C	Thyroid gland undifferentiated (anaplastic) carcinoma	Thyroid	Carcinoma	0.832
0.247	0.047	SW480	Colon adenocarcinoma	Colon	Adenocarcinoma	0.932
0.247	0.111	SW-620	Colon adenocarcinoma	Colon	Adenocarcinoma	0.887

0.246	0.124	AGS	Gastric adenocarcinoma	Stomach	Adenocarcinoma	0.875
0.241	0.089	MRC5	Embryonic lung fibroblast	Lung	Normal	0.916
0.237	0.124	SR	Adult immunoblastic lymphoma	Haematopoietic and lymphoid tissue	Lymphoma	0.877
0.235	0.142	U-266	Plasma cell myeloma	Blood	Myeloma	0.870
0.216	0.024	T98G	Glioblastoma	Brain	Carcinoma	0.912
0.209	0.174	A-427	Lung carcinoma	Lung	Carcinoma	0.872
0.205	0.202	OVCAR-5	Ovarian adenocarcinoma	Ovarium	Adenocarcinoma	0.866
0.202	0.014	IMR-32	Neuroblastoma	Nervous system	Neuroblastoma	0.951
0.191	0.107	KG-1	Acute myelogenous leukemia	Blood	Leukemia	0.901
0.189	0.022	NCI-H2228	Non-small cell lung cancer	Lung	Adenocarcinoma	0.920
0.188	0.170	SK-OV-3	Ovarian carcinoma	Ovarium	Carcinoma	0.896
0.180	0.077	HT-1080	Fibrosarcoma	Soft tissue	Sarcoma	0.918
0.169	0.074	A-431	Epidermoid carcinoma	Skin	Carcinoma	0.937
0.156	0.033	SJSA-1	Osteosarcoma	Bone	Sarcoma	0.940
0.145	0.067	SKM-1	Adult acute myeloid leukemia	Blood	Leukemia	0.917
0.145	0.066	Jurkat	Acute leukemic T-cells	Blood	Leukemia	0.946
0.141	0.019	KARPAS-299	Anaplastic large cell lymphoma	Haematopoietic and lymphoid tissue	Leukemia	0.939
0.135	0.086	MV4-11	Myeloid leukemia	Haematopoietic and lymphoid tissue	Leukemia	0.947
0.134	0.129	U-87MG	Glioblastoma	Brain	Blastoma	0.921
0.134	0.133	T-24	Bladder carcinoma	Urinary tract	Carcinoma	0.925
0.119	0.105	DLD-1	Colon adenocarcinoma	Colon	Adenocarcinoma	0.949
0.110	0.018	BEAS-2B	Epithelial cells	bronchial epithelium	Normal	1.000
0.047	0.013	M21	Melanoma	Skin	Melanoma	0.985
0.029	0.010	JY	EBV-positive lymphoblastoid B cell line	Blood	Normal	1.000

Table S5. CLC-Pred data of the azaheterocyclic derivative **3e**.

Pa	Pi	Cell-line	Description	Tissue/Organ	Type	IAP*
0.556	0.008	SW48	Colorectal Adenocarcinoma	Colon	Adenocarcinoma	0.806
0.536	0.032	SK-LU-1	Adenocarcinoma	Lung	Carcinoma	0.810
0.485	0.012	UMUC3	Bladder Carcinoma	Urinary tract	Carcinoma	0.810
0.453	0.010	RD	Rhabdomyosarcoma	Muscle	Normal	0.828
0.416	0.081	HCC1937	Breast Carcinoma	Breast	Carcinoma	0.806
0.402	0.022	HEL299	Fibroblasts	Lung	Normal	0.899
0.401	0.037	SK-MES-1	Squamous cell lung carcinoma	Lung	Carcinoma	0.834
0.398	0.066	SNU-5	Gastric Carcinoma	Stomach	Carcinoma	0.828
0.396	0.118	DU-4475	Breast Carcinoma	Breast	Carcinoma	0.802

0.393	0.031	NCI-H292	Mucoepidermoid Pulmonary Carcinoma	Lung	Carcinoma	0.845
0.384	0.030	HuP-T3	Pancreatic adenocarcinoma	Pancreas	Adenocarcinoma	0.868
0.366	0.200	OCI-AML2	Adult acute myeloid leukemia	Blood	Leukemia	0.803
0.363	0.117	NCI-H358	Bronchioalveolar Carcinoma	Lung; Bronchiole	Carcinoma	0.805
0.356	0.004	SW1116	Colorectal Adenocarcinoma	Colon	Adenocarcinoma	0.864
0.356	0.069	KYSE-520	Esophageal squamous cell carcinoma	Esophagus	Carcinoma	0.858
0.356	0.081	COR-L23	Lung large cell carcinoma	Lung	Carcinoma	0.834
0.349	0.129	Kasumi-1	Acute myeloblastic leukemia	Blood	Leukemia	0.816
0.348	0.053	HCC1806	Acantholytic Squamous Cell Carcinoma	Breast	Carcinoma	0.824
0.347	0.081	EOL1	Chronic eosinophilic leukemia, not otherwise specified	Blood	Leukemia	0.861
0.343	0.151	SK-MEL-1	Metastatic melanoma	Skin	Melanoma	0.808
0.340	0.034	PA-1	Ovarian carcinoma	Ovarium	Carcinoma	0.854
0.335	0.103	RS4-11	Adult B acute lymphoblastic leukemia	Bone Marrow	Leukemia	0.835
0.329	0.142	OE33	Barrett adenocarcinoma	Esophagus	Adenocarcinoma	0.805
0.323	0.287	TMD8	Diffuse large B-cell lymphoma activated B-cell type	Lymphocytes	Lymphoma	0.811
0.320	0.039	MSTO-211H	Biphasic Mesothelioma	Lung	Mesothelioma	0.878
0.313	0.224	NCI-H441	Papillary adenocarcinoma	Lung	Adenocarcinoma	0.813
0.312	0.056	SR	Adult immunoblastic lymphoma	Haematopoietic and lymphoid tissue	Lymphoma	0.877
0.312	0.061	NCI-H727	Carcinoid	Lung; Bronchus	Carcinoma	0.828
0.312	0.214	A172	Glioblastoma	Brain	Glioblastoma	0.807
0.311	0.193	CAL-51	Breast carcinoma	Breast	Carcinoma	0.824
0.310	0.011	CFPAC-1	Pancreatic carcinoma	Pancreas	Carcinoma	0.905
0.309	0.283	A2780cisR	Cisplatin-resistant ovarian carcinoma	Ovarium	Carcinoma	0.838
0.306	0.185	HT1197	Carcinoma	Urinary bladder	Carcinoma	0.815
0.304	0.083	NCI-H520	Squamous Cell Carcinoma	Lung	Carcinoma	0.837
0.296	0.211	MCF7	Breast carcinoma	Breast	Carcinoma	0.836
0.284	0.068	NCI-H1581	Non Small Cell Lung Cancer	Lung	Carcinoma	0.871
0.284	0.095	HOS	Osteosarcoma	Bone	Sarcoma	0.863
0.277	0.087	CAL-27	Squamous Cell Carcinoma	Tongue	Carcinoma	0.854
0.276	0.129	RPMI-7951	Malignant Melanoma	Skin	Melanoma	0.854
0.274	0.188	NCI-H661	Lung carcinoma	Lung	Carcinoma	0.831
0.267	0.106	A2058	Melanoma	Skin	Melanoma	0.859
0.256	0.104	AGS	Gastric adenocarcinoma	Stomach	Adenocarcinoma	0.875
0.250	0.192	8505C	Thyroid gland undifferentiated (anaplastic) carcinoma	Thyroid	Carcinoma	0.832

0.247	0.222	NCI-H1650	Bronchoalveolar carcinoma	Lung	Adenocarcinoma	0.839
0.245	0.124	U-266	Plasma cell myeloma	Blood	Myeloma	0.870
0.243	0.134	OVCAR-5	Ovarian adenocarcinoma	Ovarium	Adenocarcinoma	0.866
0.242	0.039	KG-1	Acute myelogenous leukemia	Blood	Leukemia	0.901
0.242	0.172	MKN-74	Gastric tubular adenocarcinoma	Stomach	Adenocarcinoma	0.856
0.239	0.045	SUM149PT	Breast inflammatory carcinoma	Breast	Carcinoma	0.983
0.228	0.067	OCI-Ly10	Diffuse large B-cell lymphoma activated B-cell type	Lymphoid tissue	Lymphoma	0.919
0.228	0.122	H9c2	Rat DB1X heart myoblast cell line	Heart	Myoblast	0.897
0.227	0.182	H9	T-lymphoid	Haematopoietic and lymphoid tissue	Leukemia	0.872
0.224	0.192	UACC-257	Melanoma	Skin	Melanoma	0.861
0.221	0.022	T98G	Glioblastoma	Brain	Carcinoma	0.912
0.211	0.123	U2OS	Osteosarcoma	Bone	Sarcoma	0.885
0.210	0.192	MDA-MB-361	Breast adenocarcinoma	Breast	Adenocarcinoma	0.872
0.202	0.199	A-427	Lung carcinoma	Lung	Carcinoma	0.872
0.191	0.019	NCI-H2228	Non-small cell lung cancer	Lung	Adenocarcinoma	0.920
0.173	0.041	Jurkat	Acute leukemic T-cells	Blood	Leukemia	0.946
0.163	0.017	SJSA-1	Osteosarcoma	Bone	Sarcoma	0.940
0.160	0.112	HT-1080	Fibrosarcoma	Soft tissue	Sarcoma	0.918
0.159	0.030	IMR-32	Neuroblastoma	Nervous system	Neuroblastoma	0.951
0.148	0.054	SKM-1	Adult acute myeloid leukemia	Blood	Leukemia	0.917
0.143	0.017	KARPAS-299	Anaplastic large cell lymphoma	Haematopoietic and lymphoid tissue	Leukemia	0.939
0.134	0.129	U-87MG	Glioblastoma	Brain	Blastoma	0.921
0.132	0.118	SW480	Colon adenocarcinoma	Colon	Adenocarcinoma	0.932
0.128	0.099	MV4-11	Myeloid leukemia	Haematopoietic and lymphoid tissue	Leukemia	0.947
0.117	0.103	RWPE-1	Prostatic epithelial cell line	Prostate	Normal	0.922
0.102	0.096	Ramos	Burkitts lymhoma B-cells	Blood	Leukemia	0.958
0.086	0.031	NCI-H2286	Small cell lung cancer	Lung	Adenocarcinoma	0.968
0.059	0.046	NAMALVA	EBV-related Burkitt lymphoma	Blood	Lymphoma	0.986
0.045	0.038	Daudi	Burkitts Lymphoma	Blood	Lymphoma	0.990
0.044	0.017	M21	Melanoma	Skin	Melanoma	0.985
0.039	0.005	JY	EBV-positive lymphoblastoid B cell line	Blood	Normal	1.000

Table S6. CLC-Pred data of the azaheterocyclic derivative **3f**.

Pa	Pi	Cell-line	Description	Tissue/Organ	Type	IAP*
0.556	0.008	SW48	Colorectal Adenocarcinoma	Colon	Adenocarcinoma	0.806
0.537	0.031	SK-LU-1	Adenocarcinoma	Lung	Carcinoma	0.810

0.485	0.012	UMUC3	Bladder Carcinoma	Urinary tract	Carcinoma	0.810
0.446	0.012	RD	Rhabdomyosarcoma	Muscle	Normal	0.828
0.430	0.065	HCC1937	Breast Carcinoma	Breast	Carcinoma	0.806
0.408	0.023	NCI-H292	Mucoepidermoid Pulmonary Carcinoma	Lung	Carcinoma	0.845
0.406	0.058	SNU-5	Gastric Carcinoma	Stomach	Carcinoma	0.828
0.401	0.037	SK-MES-1	Squamous cell lung carcinoma	Lung	Carcinoma	0.834
0.394	0.024	HEL299	Fibroblasts	Lung	Normal	0.899
0.394	0.119	DU-4475	Breast Carcinoma	Breast	Carcinoma	0.802
0.384	0.180	OCI-AML2	Adult acute myeloid leukemia	Blood	Leukemia	0.803
0.381	0.031	HuP-T3	Pancreatic adenocarcinoma	Pancreas	Adenocarcinoma	0.868
0.374	0.103	NCI-H358	Bronchioalveolar Carcinoma	Lung; Bronchiole	Carcinoma	0.805
0.357	0.068	KYSE-520	Esophageal squamous cell carcinoma	Esophagus	Carcinoma	0.858
0.353	0.005	SW1116	Colorectal Adenocarcinoma	Colon	Adenocarcinoma	0.864
0.352	0.137	SK-MEL-1	Metastatic melanoma	Skin	Melanoma	0.808
0.347	0.054	HCC1806	Acantholytic Squamous Cell Carcinoma	Breast	Carcinoma	0.824
0.343	0.086	EOL1	Chronic eosinophilic leukemia, not otherwise specified	Blood	Leukemia	0.861
0.342	0.238	GIST48	Gastrointestinal stromal tumor	Intestine	Carcinoma	0.805
0.341	0.106	COR-L23	Lung large cell carcinoma	Lung	Carcinoma	0.834
0.340	0.142	Kasumi-1	Acute myeloblastic leukemia	Blood	Leukemia	0.816
0.337	0.036	PA-1	Ovarian carcinoma	Ovarium	Carcinoma	0.854
0.332	0.192	NCI-H441	Papillary adenocarcinoma	Lung	Adenocarcinoma	0.813
0.317	0.130	RS4-11	Adult B acute lymphoblastic leukemia	Bone Marrow	Leukemia	0.835
0.312	0.174	HT1197	Carcinoma	Urinary bladder	Carcinoma	0.815
0.310	0.066	NCI-H727	Carcinoid	Lung; Bronchus	Carcinoma	0.828
0.309	0.011	CFPAC-1	Pancreatic carcinoma	Pancreas	Carcinoma	0.905
0.309	0.048	MSTO-211H	Biphasic Mesothelioma	Lung	Mesothelioma	0.878
0.308	0.175	OE33	Barrett adenocarcinoma	Esophagus	Adenocarcinoma	0.805
0.307	0.059	SR	Adult immunoblastic lymphoma	Haematopoietic and lymphoid tissue	Lymphoma	0.877
0.306	0.079	NCI-H520	Squamous Cell Carcinoma	Lung	Carcinoma	0.837
0.303	0.212	CAL-51	Breast carcinoma	Breast	Carcinoma	0.824
0.302	0.242	A172	Glioblastoma	Brain	Glioblastoma	0.807
0.292	0.101	RPMI-7951	Malignant Melanoma	Skin	Melanoma	0.854
0.286	0.065	NCI-H1581	Non Small Cell Lung Cancer	Lung	Carcinoma	0.871
0.284	0.072	CAL-27	Squamous Cell Carcinoma	Tongue	Carcinoma	0.854
0.281	0.223	MCF7	Breast carcinoma	Breast	Carcinoma	0.836
0.272	0.114	HOS	Osteosarcoma	Bone	Sarcoma	0.863

0.271	0.197	NCI-H661	Lung carcinoma	Lung	Carcinoma	0.831
0.271	0.245	GIST430	Gastrointestinal stromal tumor	Intestine	Carcinoma	0.820
0.268	0.105	A2058	Melanoma	Skin	Melanoma	0.859
0.255	0.029	KG-1	Acute myelogenous leukemia	Blood	Leukemia	0.901
0.251	0.190	8505C	Thyroid gland undifferentiated (anaplastic) carcinoma	Thyroid	Carcinoma	0.832
0.247	0.041	SUM149PT	Breast inflammatory carcinoma	Breast	Carcinoma	0.983
0.246	0.124	AGS	Gastric adenocarcinoma	Stomach	Adenocarcinoma	0.875
0.245	0.124	U-266	Plasma cell myeloma	Blood	Myeloma	0.870
0.242	0.135	OVCAR-5	Ovarian adenocarcinoma	Ovarium	Adenocarcinoma	0.866
0.242	0.238	NCI-H1650	Bronchoalveolar carcinoma	Lung	Adenocarcinoma	0.839
0.233	0.175	UACC-257	Melanoma	Skin	Melanoma	0.861
0.232	0.116	H9c2	Rat DB1X heart myoblast cell line	Heart	Myoblast	0.897
0.218	0.081	OCI-Ly10	Diffuse large B-cell lymphoma activated B-cell type	Lymphoid tissue	Lymphoma	0.919
0.215	0.205	H9	T-lymphoid	Haematopoietic and lymphoid tissue	Leukemia	0.872
0.209	0.029	T98G	Glioblastoma	Brain	Carcinoma	0.912
0.206	0.137	U2OS	Osteosarcoma	Bone	Sarcoma	0.885
0.191	0.019	NCI-H2228	Non-small cell lung cancer	Lung	Adenocarcinoma	0.920
0.179	0.037	Jurkat	Acute leukemic T-cells	Blood	Leukemia	0.946
0.161	0.021	SJSA-1	Osteosarcoma	Bone	Sarcoma	0.940
0.154	0.125	HT-1080	Fibrosarcoma	Soft tissue	Sarcoma	0.918
0.149	0.051	SKM-1	Adult acute myeloid leukemia	Blood	Leukemia	0.917
0.148	0.036	IMR-32	Neuroblastoma	Nervous system	Neuroblastoma	0.951
0.143	0.017	KARPAS-299	Anaplastic large cell lymphoma	Haematopoietic and lymphoid tissue	Leukemia	0.939
0.136	0.113	SW480	Colon adenocarcinoma	Colon	Adenocarcinoma	0.932
0.125	0.106	MV4-11	Myeloid leukemia	Haematopoietic and lymphoid tissue	Leukemia	0.947
0.120	0.091	RWPE-1	Prostatic epithelial cell line	Prostate	Normal	0.922
0.103	0.095	Ramos	Burkitts lymhoma B-cells	Blood	Leukemia	0.958
0.088	0.028	NCI-H2286	Small cell lung cancer	Lung	Adenocarcinoma	0.968
0.045	0.038	Daudi	Burkitts Lymphoma	Blood	Lymphoma	0.990
0.043	0.017	M21	Melanoma	Skin	Melanoma	0.985
0.039	0.005	JY	EBV-positive lymphoblastoid B cell line	Blood	Normal	1.000

Table S7. CLC-Pred data of the azaheterocyclic derivative **3g**.

Pa	Pi	Cell-line	Description	Tissue/Organ	Type	IAP*

0.612	0.105	A2780cisR	Cisplatin-resistant ovarian carcinoma	Ovarium	Carcinoma	0.838
0.606	0.005	SW48	Colorectal Adenocarcinoma	Colon	Adenocarcinoma	0.806
0.515	0.044	SK-LU-1	Adenocarcinoma	Lung	Carcinoma	0.810
0.480	0.097	MCF7	Breast carcinoma	Breast	Carcinoma	0.836
0.449	0.014	HEL299	Fibroblasts	Lung	Normal	0.899
0.442	0.029	UMUC3	Bladder Carcinoma	Urinary tract	Carcinoma	0.810
0.438	0.056	HCC1937	Breast Carcinoma	Breast	Carcinoma	0.806
0.424	0.020	SK-MES-1	Squamous cell lung carcinoma	Lung	Carcinoma	0.834
0.414	0.096	DU-4475	Breast Carcinoma	Breast	Carcinoma	0.802
0.388	0.076	SNU-5	Gastric Carcinoma	Stomach	Carcinoma	0.828
0.383	0.038	NCI-H292	Mucoepidermoid Pulmonary Carcinoma	Lung	Carcinoma	0.845
0.383	0.047	COR-L23	Lung large cell carcinoma	Lung	Carcinoma	0.834
0.382	0.030	HuP-T3	Pancreatic adenocarcinoma	Pancreas	Adenocarcinoma	0.868
0.377	0.033	RD	Rhabdomyosarcoma	Muscle	Normal	0.828
0.369	0.080	OE33	Barrett adenocarcinoma	Esophagus	Adenocarcinoma	0.805
0.367	0.004	SW1116	Colorectal Adenocarcinoma	Colon	Adenocarcinoma	0.864
0.363	0.064	EOL1	Chronic eosinophilic leukemia, not otherwise specified	Blood	Leukemia	0.861
0.362	0.118	NCI-H358	Bronchioalveolar Carcinoma	Lung; Bronchiole	Carcinoma	0.805
0.361	0.083	A172	Glioblastoma	Brain	Glioblastoma	0.807
0.361	0.206	OCI-AML2	Adult acute myeloid leukemia	Blood	Leukemia	0.803
0.359	0.125	SK-MEL-1	Metastatic melanoma	Skin	Melanoma	0.808
0.353	0.073	KYSE-520	Esophageal squamous cell carcinoma	Esophagus	Carcinoma	0.858
0.351	0.159	NCI-H441	Papillary adenocarcinoma	Lung	Adenocarcinoma	0.813
0.348	0.053	HCC1806	Acantholytic Squamous Cell Carcinoma	Breast	Carcinoma	0.824
0.345	0.031	PA-1	Ovarian carcinoma	Ovarium	Carcinoma	0.854
0.338	0.131	CAL-51	Breast carcinoma	Breast	Carcinoma	0.824
0.336	0.026	MSTO-211H	Biphasic Mesothelioma	Lung	Mesothelioma	0.878
0.332	0.271	TMD8	Diffuse large B-cell lymphoma activated B-cell type	Lymphocytes	Lymphoma	0.811
0.329	0.140	HT1197	Carcinoma	Urinary bladder	Carcinoma	0.815
0.313	0.060	NCI-H727	Carcinoid	Lung; Bronchus	Carcinoma	0.828
0.313	0.061	HOS	Osteosarcoma	Bone	Sarcoma	0.863
0.310	0.074	NCI-H520	Squamous Cell Carcinoma	Lung	Carcinoma	0.837
0.309	0.166	CAKI-2	Kidney carcinoma	Kidney	Carcinoma	0.829
0.307	0.080	RPMI-7951	Malignant Melanoma	Skin	Melanoma	0.854
0.307	0.188	Kasumi-1	Acute myeloblastic leukemia	Blood	Leukemia	0.816
0.304	0.117	HCC1954	Breast Carcinoma	Breast	Carcinoma	0.829

0.297	0.065	SR	Adult immunoblastic lymphoma	Haematopoietic and lymphoid tissue	Lymphoma	0.877
0.295	0.163	RS4-11	Adult B acute lymphoblastic leukemia	Bone Marrow	Leukemia	0.835
0.293	0.013	CFPAC-1	Pancreatic carcinoma	Pancreas	Carcinoma	0.905
0.285	0.059	AGS	Gastric adenocarcinoma	Stomach	Adenocarcinoma	0.875
0.285	0.071	CAL-27	Squamous Cell Carcinoma	Tongue	Carcinoma	0.854
0.284	0.069	RCC4	Clear cell renal cell carcinoma	Kidney	Carcinoma	0.845
0.284	0.185	DMS-114	Lung carcinoma	Lung	Carcinoma	0.834
0.278	0.130	NCI-H1650	Bronchoalveolar carcinoma	Lung	Adenocarcinoma	0.839
0.278	0.232	GIST430	Gastrointestinal stromal tumor	Intestine	Carcinoma	0.820
0.277	0.178	NCI-H661	Lung carcinoma	Lung	Carcinoma	0.831
0.276	0.097	A2058	Melanoma	Skin	Melanoma	0.859
0.268	0.095	NCI-H1581	Non Small Cell Lung Cancer	Lung	Carcinoma	0.871
0.266	0.119	H9	T-lymphoid	Haematopoietic and lymphoid tissue	Leukemia	0.872
0.265	0.152	8505C	Thyroid gland undifferentiated (anaplastic) carcinoma	Thyroid	Carcinoma	0.832
0.247	0.128	OVCAR-5	Ovarian adenocarcinoma	Ovarium	Adenocarcinoma	0.866
0.243	0.014	T98G	Glioblastoma	Brain	Carcinoma	0.912
0.243	0.128	U-266	Plasma cell myeloma	Blood	Myeloma	0.870
0.231	0.102	MRC5	Embryonic lung fibroblast	Lung	Normal	0.916
0.230	0.089	U2OS	Osteosarcoma	Bone	Sarcoma	0.885
0.229	0.120	A-427	Lung carcinoma	Lung	Carcinoma	0.872
0.229	0.128	SK-OV-3	Ovarian carcinoma	Ovarium	Carcinoma	0.896
0.218	0.116	HOP-18	Non-small cell lung carcinoma	Lung	Carcinoma	0.933
0.206	0.146	CAPAN-1	Pancreas Adenocarcinoma	Pancreas	Adenocarcinoma	0.889
0.199	0.180	RKO	Colon carcinoma	Colon	Carcinoma	0.879
0.194	0.063	HT-1080	Fibrosarcoma	Soft tissue	Sarcoma	0.918
0.194	0.099	KG-1	Acute myelogenous leukemia	Blood	Leukemia	0.901
0.193	0.016	NCI-H2228	Non-small cell lung cancer	Lung	Adenocarcinoma	0.920
0.193	0.071	SW480	Colon adenocarcinoma	Colon	Adenocarcinoma	0.932
0.190	0.186	SW1990	Pancreatic adenocarcinoma	Pancreas	Adenocarcinoma	0.876
0.175	0.165	OCI-Ly10	Diffuse large B-cell lymphoma activated B-cell type	Lymphoid tissue	Lymphoma	0.919
0.174	0.126	SK-HEP1	Hepatocellular carcinoma	Liver	Carcinoma	0.912
0.173	0.164	NCI-H460	Non-small cell lung carcinoma	Lung	Carcinoma	0.906
0.171	0.024	IMR-32	Neuroblastoma	Nervous system	Neuroblastoma	0.951
0.171	0.065	Mahlavu	Hepatocellular carcinoma	Liver	Carcinoma	0.937
0.160	0.022	SJSA-1	Osteosarcoma	Bone	Sarcoma	0.940
0.148	0.057	SKM-1	Adult acute myeloid leukemia	Blood	Leukemia	0.917

0.146	0.015	KARPAS-299	Anaplastic large cell lymphoma	Haematopoietic and lymphoid tissue	Leukemia	0.939
0.131	0.082	Jurkat	Acute leukemic T-cells	Blood	Leukemia	0.946
0.129	0.090	DLD-1	Colon adenocarcinoma	Colon	Adenocarcinoma	0.949
0.124	0.116	KOPTK1	Childhood T acute lymphoblastic leukemia	Blood	Leukemia	0.808
0.117	0.061	KK-47	Bladder carcinoma	Bladder	Carcinoma	0.887
0.099	0.029	BEAS-2B	Epithelial cells	bronchial epithelium	Normal	1.000
0.086	0.076	HA22T	Hepatocellular carcinoma	Liver	Carcinoma	0.946
0.045	0.015	M21	Melanoma	Skin	Melanoma	0.985
0.030	0.010	JY	EBV-positive lymphoblastoid B cell line	Blood	Normal	1.000
0.011	0.009	QG-56	Squamous cell lung carcinoma	Lung	Carcinoma	1.000

Table S8. CLC-Pred data of the azaheterocyclic derivative **3h**.

Pa	Pi	Cell-line	Description	Tissue/Organ	Type	IAP*
0.633	0.005	SW48	Colorectal Adenocarcinoma	Colon	Adenocarcinoma	0.806
0.571	0.124	A2780cisR	Cisplatin-resistant ovarian carcinoma	Ovarium	Carcinoma	0.838
0.518	0.042	SK-LU-1	Adenocarcinoma	Lung	Carcinoma	0.810
0.469	0.103	MCF7	Breast carcinoma	Breast	Carcinoma	0.836
0.442	0.052	HCC1937	Breast Carcinoma	Breast	Carcinoma	0.806
0.440	0.016	HEL299	Fibroblasts	Lung	Normal	0.899
0.437	0.031	UMUC3	Bladder Carcinoma	Urinary tract	Carcinoma	0.810
0.420	0.022	SK-MES-1	Squamous cell lung carcinoma	Lung	Carcinoma	0.834
0.403	0.109	DU-4475	Breast Carcinoma	Breast	Carcinoma	0.802
0.388	0.076	SNU-5	Gastric Carcinoma	Stomach	Carcinoma	0.828
0.383	0.047	COR-L23	Lung large cell carcinoma	Lung	Carcinoma	0.834
0.382	0.182	OCI-AML2	Adult acute myeloid leukemia	Blood	Leukemia	0.803
0.378	0.041	NCI-H292	Mucoepidermoid Pulmonary Carcinoma	Lung	Carcinoma	0.845
0.375	0.034	RD	Rhabdomyosarcoma	Muscle	Normal	0.828
0.372	0.036	HuP-T3	Pancreatic adenocarcinoma	Pancreas	Adenocarcinoma	0.868
0.365	0.114	NCI-H358	Bronchioalveolar Carcinoma	Lung; Bronchiole	Carcinoma	0.805
0.364	0.118	SK-MEL-1	Metastatic melanoma	Skin	Melanoma	0.808
0.362	0.004	SW1116	Colorectal Adenocarcinoma	Colon	Adenocarcinoma	0.864
0.361	0.091	OE33	Barrett adenocarcinoma	Esophagus	Adenocarcinoma	0.805
0.356	0.071	EOL1	Chronic eosinophilic leukemia, not otherwise specified	Blood	Leukemia	0.861
0.354	0.100	A172	Glioblastoma	Brain	Glioblastoma	0.807
0.352	0.157	NCI-H441	Papillary adenocarcinoma	Lung	Adenocarcinoma	0.813

0.343	0.089	KYSE-520	Esophageal squamous cell carcinoma	Esophagus	Carcinoma	0.858
0.342	0.061	HCC1806	Acantholytic Squamous Cell Carcinoma	Breast	Carcinoma	0.824
0.339	0.035	PA-1	Ovarian carcinoma	Ovarium	Carcinoma	0.854
0.336	0.136	CAL-51	Breast carcinoma	Breast	Carcinoma	0.824
0.332	0.044	HOS	Osteosarcoma	Bone	Sarcoma	0.863
0.330	0.031	MSTO-211H	Biphasic Mesothelioma	Lung	Mesothelioma	0.878
0.325	0.054	NCI-H520	Squamous Cell Carcinoma	Lung	Carcinoma	0.837
0.317	0.164	HT1197	Carcinoma	Urinary bladder	Carcinoma	0.815
0.315	0.058	NCI-H727	Carcinoid	Lung; Bronchus	Carcinoma	0.828
0.313	0.303	TMD8	Diffuse large B-cell lymphoma activated B-cell type	Lymphocytes	Lymphoma	0.811
0.303	0.119	HCC1954	Breast Carcinoma	Breast	Carcinoma	0.829
0.302	0.062	SR	Adult immunoblastic lymphoma	Haematopoietic and lymphoid tissue	Lymphoma	0.877
0.300	0.090	RPMI-7951	Malignant Melanoma	Skin	Melanoma	0.854
0.299	0.189	CAKI-2	Kidney carcinoma	Kidney	Carcinoma	0.829
0.298	0.158	RS4-11	Adult B acute lymphoblastic leukemia	Bone Marrow	Leukemia	0.835
0.294	0.084	SK-OV-3	Ovarian carcinoma	Ovarium	Carcinoma	0.896
0.293	0.050	AGS	Gastric adenocarcinoma	Stomach	Adenocarcinoma	0.875
0.291	0.170	DMS-114	Lung carcinoma	Lung	Carcinoma	0.834
0.282	0.015	CFPAC-1	Pancreatic carcinoma	Pancreas	Carcinoma	0.905
0.280	0.080	CAL-27	Squamous Cell Carcinoma	Tongue	Carcinoma	0.854
0.280	0.224	Kasumi-1	Acute myeloblastic leukemia	Blood	Leukemia	0.816
0.274	0.110	H9	T-lymphoid	Haematopoietic and lymphoid tissue	Leukemia	0.872
0.274	0.141	NCI-H1650	Bronchoalveolar carcinoma	Lung	Adenocarcinoma	0.839
0.273	0.192	NCI-H661	Lung carcinoma	Lung	Carcinoma	0.831
0.272	0.136	8505C	Thyroid gland undifferentiated (anaplastic) carcinoma	Thyroid	Carcinoma	0.832
0.270	0.103	A2058	Melanoma	Skin	Melanoma	0.859
0.266	0.099	NCI-H1581	Non Small Cell Lung Cancer	Lung	Carcinoma	0.871
0.258	0.114	OVCAR-5	Ovarian adenocarcinoma	Ovarium	Adenocarcinoma	0.866
0.240	0.230	EKVX	Non-small cell lung carcinoma	Lung	Carcinoma	0.841
0.238	0.093	MRC5	Embryonic lung fibroblast	Lung	Normal	0.916
0.235	0.016	T98G	Glioblastoma	Brain	Carcinoma	0.912
0.234	0.143	U-266	Plasma cell myeloma	Blood	Myeloma	0.870
0.233	0.121	RCC4	Clear cell renal cell carcinoma	Kidney	Carcinoma	0.845
0.229	0.091	U2OS	Osteosarcoma	Bone	Sarcoma	0.885
0.223	0.212	MKN-74	Gastric tubular adenocarcinoma	Stomach	Adenocarcinoma	0.856
0.221	0.140	A-427	Lung carcinoma	Lung	Carcinoma	0.872

0.212	0.162	SW1990	Pancreatic adenocarcinoma	Pancreas	Adenocarcinoma	0.876
0.209	0.138	CAPAN-1	Pancreas Adenocarcinoma	Pancreas	Adenocarcinoma	0.889
0.207	0.053	HT-1080	Fibrosarcoma	Soft tissue	Sarcoma	0.918
0.205	0.156	RKO	Colon carcinoma	Colon	Carcinoma	0.879
0.202	0.067	SW480	Colon adenocarcinoma	Colon	Adenocarcinoma	0.932
0.198	0.138	NCI-H460	Non-small cell lung carcinoma	Lung	Carcinoma	0.906
0.197	0.176	SW-620	Colon adenocarcinoma	Colon	Adenocarcinoma	0.887
0.194	0.145	HOP-18	Non-small cell lung carcinoma	Lung	Carcinoma	0.933
0.193	0.017	NCI-H2228	Non-small cell lung cancer	Lung	Adenocarcinoma	0.920
0.191	0.106	KG-1	Acute myelogenous leukemia	Blood	Leukemia	0.901
0.188	0.137	UACC-62	Melanoma	Skin	Melanoma	0.904
0.184	0.019	IMR-32	Neuroblastoma	Nervous system	Neuroblastoma	0.951
0.169	0.136	SK-HEP1	Hepatocellular carcinoma	Liver	Carcinoma	0.912
0.159	0.025	SJSA-1	Osteosarcoma	Bone	Sarcoma	0.940
0.159	0.080	Mahlavu	Hepatocellular carcinoma	Liver	Carcinoma	0.937
0.145	0.068	SKM-1	Adult acute myeloid leukemia	Blood	Leukemia	0.917
0.142	0.018	KARPAS-299	Anaplastic large cell lymphoma	Haematopoietic and lymphoid tissue	Leukemia	0.939
0.133	0.085	DLD-1	Colon adenocarcinoma	Colon	Adenocarcinoma	0.949
0.131	0.082	Jurkat	Acute leukemic T-cells	Blood	Leukemia	0.946
0.116	0.106	RWPE-1	Prostatic epithelial cell line	Prostate	Normal	0.922
0.090	0.064	HA22T	Hepatocellular carcinoma	Liver	Carcinoma	0.946
0.082	0.061	BEAS-2B	Epithelial cells	bronchial epithelium	Normal	1.000
0.044	0.016	M21	Melanoma	Skin	Melanoma	0.985
0.028	0.011	JY	EBV-positive lymphoblastoid B cell line	Blood	Normal	1.000
0.012	0.009	QG-56	Squamous cell lung carcinoma	Lung	Carcinoma	1.000

Table S9. CLC-Pred data of the azaheterocyclic derivative **3i**.

Pa	Pi	Cell-line	Description	Tissue/Organ	Type	IAP*
0.559	0.008	SW48	Colorectal Adenocarcinoma	Colon	Adenocarcinoma	0.806
0.493	0.091	MCF7	Breast carcinoma	Breast	Carcinoma	0.836
0.492	0.063	SK-LU-1	Adenocarcinoma	Lung	Carcinoma	0.810
0.454	0.014	HEL299	Fibroblasts	Lung	Normal	0.899
0.443	0.028	UMUC3	Bladder Carcinoma	Urinary tract	Carcinoma	0.810
0.439	0.055	HCC1937	Breast Carcinoma	Breast	Carcinoma	0.806
0.420	0.022	SK-MES-1	Squamous cell lung carcinoma	Lung	Carcinoma	0.834
0.393	0.183	TMD8	Diffuse large B-cell lymphoma activated B-cell type	Lymphocytes	Lymphoma	0.811
0.388	0.028	RD	Rhabdomyosarcoma	Muscle	Normal	0.828

0.387	0.128	DU-4475	Breast Carcinoma	Breast	Carcinoma	0.802
0.379	0.041	NCI-H292	Mucoepidermoid Pulmonary Carcinoma	Lung	Carcinoma	0.845
0.376	0.034	HuP-T3	Pancreatic adenocarcinoma	Pancreas	Adenocarcinoma	0.868
0.376	0.089	SNU-5	Gastric Carcinoma	Stomach	Carcinoma	0.828
0.374	0.103	NCI-H358	Bronchioalveolar Carcinoma	Lung; Bronchiole	Carcinoma	0.805
0.369	0.080	OE33	Barrett adenocarcinoma	Esophagus	Adenocarcinoma	0.805
0.367	0.064	COR-L23	Lung large cell carcinoma	Lung	Carcinoma	0.834
0.367	0.072	A172	Glioblastoma	Brain	Glioblastoma	0.807
0.346	0.005	SW1116	Colorectal Adenocarcinoma	Colon	Adenocarcinoma	0.864
0.345	0.118	CAL-51	Breast carcinoma	Breast	Carcinoma	0.824
0.345	0.253	A2780cisR	Cisplatin-resistant ovarian carcinoma	Ovarium	Carcinoma	0.838
0.343	0.089	KYSE-520	Esophageal squamous cell carcinoma	Esophagus	Carcinoma	0.858
0.342	0.060	HCC1806	Acantholytic Squamous Cell Carcinoma	Breast	Carcinoma	0.824
0.341	0.155	SK-MEL-1	Metastatic melanoma	Skin	Melanoma	0.808
0.340	0.089	EOL1	Chronic eosinophilic leukemia, not otherwise specified	Blood	Leukemia	0.861
0.337	0.232	OCI-AML2	Adult acute myeloid leukemia	Blood	Leukemia	0.803
0.326	0.035	MSTO-211H	Biphasic Mesothelioma	Lung	Mesothelioma	0.878
0.325	0.047	PA-1	Ovarian carcinoma	Ovarium	Carcinoma	0.854
0.322	0.048	NCI-H727	Carcinoid	Lung; Bronchus	Carcinoma	0.828
0.319	0.127	RS4-11	Adult B acute lymphoblastic leukemia	Bone Marrow	Leukemia	0.835
0.318	0.053	SR	Adult immunoblastic lymphoma	Haematopoietic and lymphoid tissue	Lymphoma	0.877
0.318	0.066	RPMI-7951	Malignant Melanoma	Skin	Melanoma	0.854
0.316	0.165	HT1197	Carcinoma	Urinary bladder	Carcinoma	0.815
0.316	0.219	NCI-H441	Papillary adenocarcinoma	Lung	Adenocarcinoma	0.813
0.314	0.067	NCI-H520	Squamous Cell Carcinoma	Lung	Carcinoma	0.837
0.313	0.089	HCC1954	Breast Carcinoma	Breast	Carcinoma	0.829
0.308	0.186	Kasumi-1	Acute myeloblastic leukemia	Blood	Leukemia	0.816
0.291	0.058	NCI-H1581	Non Small Cell Lung Cancer	Lung	Carcinoma	0.871
0.287	0.151	NCI-H661	Lung carcinoma	Lung	Carcinoma	0.831
0.280	0.081	CAL-27	Squamous Cell Carcinoma	Tongue	Carcinoma	0.854
0.277	0.106	HOS	Osteosarcoma	Bone	Sarcoma	0.863
0.276	0.201	DMS-114	Lung carcinoma	Lung	Carcinoma	0.834
0.274	0.073	AGS	Gastric adenocarcinoma	Stomach	Adenocarcinoma	0.875
0.271	0.100	OVCAR-5	Ovarian adenocarcinoma	Ovarium	Adenocarcinoma	0.866
0.264	0.168	NCI-H1650	Bronchoalveolar carcinoma	Lung	Adenocarcinoma	0.839

0.261	0.162	8505C	Thyroid gland undifferentiated (anaplastic) carcinoma	Thyroid	Carcinoma	0.832
0.258	0.131	H9	T-lymphoid	Haematopoietic and lymphoid tissue	Leukemia	0.872
0.255	0.020	CFPAC-1	Pancreatic carcinoma	Pancreas	Carcinoma	0.905
0.253	0.121	A2058	Melanoma	Skin	Melanoma	0.859
0.251	0.082	A-427	Lung carcinoma	Lung	Carcinoma	0.872
0.249	0.210	EKVX	Non-small cell lung carcinoma	Lung	Carcinoma	0.841
0.244	0.013	T98G	Glioblastoma	Brain	Carcinoma	0.912
0.240	0.120	SK-OV-3	Ovarian carcinoma	Ovarium	Carcinoma	0.896
0.239	0.172	Hs-578T	Invasive ductal breast carcinoma	Breast	Carcinoma	0.869
0.238	0.197	RL	Non-Hodgkin's Lymphoma	Ascites	Lymphoma	0.833
0.236	0.140	U-266	Plasma cell myeloma	Blood	Myeloma	0.870
0.221	0.196	TK-10	Renal carcinoma	Kidney	Carcinoma	0.854
0.219	0.116	RKO	Colon carcinoma	Colon	Carcinoma	0.879
0.216	0.061	SW480	Colon adenocarcinoma	Colon	Adenocarcinoma	0.932
0.213	0.114	CCRF-CEM	Childhood T acute lymphoblastic leukemia	Blood	Leukemia	0.913
0.213	0.205	CL1-0	Lung adenocarcinoma	Lung	Adenocarcinoma	0.830
0.209	0.159	SW-620	Colon adenocarcinoma	Colon	Adenocarcinoma	0.887
0.196	0.013	NCI-H2228	Non-small cell lung cancer	Lung	Adenocarcinoma	0.920
0.195	0.062	HT-1080	Fibrosarcoma	Soft tissue	Sarcoma	0.918
0.187	0.018	IMR-32	Neuroblastoma	Nervous system	Neuroblastoma	0.951
0.179	0.117	SK-HEP1	Hepatocellular carcinoma	Liver	Carcinoma	0.912
0.178	0.158	NCI-H460	Non-small cell lung carcinoma	Lung	Carcinoma	0.906
0.175	0.166	OCI-Ly10	Diffuse large B-cell lymphoma activated B-cell type	Lymphoid tissue	Lymphoma	0.919
0.156	0.084	A-431	Epidermoid carcinoma	Skin	Carcinoma	0.937
0.149	0.013	KARPAS-299	Anaplastic large cell lymphoma	Haematopoietic and lymphoid tissue	Leukemia	0.939
0.145	0.112	T-24	Bladder carcinoma	Urinary tract	Carcinoma	0.925
0.143	0.074	SKM-1	Adult acute myeloid leukemia	Blood	Leukemia	0.917
0.139	0.111	Mahlavu	Hepatocellular carcinoma	Liver	Carcinoma	0.937
0.137	0.101	SJSA-1	Osteosarcoma	Bone	Sarcoma	0.940
0.135	0.128	U-87MG	Glioblastoma	Brain	Blastoma	0.921
0.110	0.087	PC-14	Lung adenocarcinoma	Lung	Carcinoma	0.932
0.086	0.074	HA22T	Hepatocellular carcinoma	Liver	Carcinoma	0.946
0.062	0.038	NAMALVA	EBV-related Burkitt lymphoma	Blood	Lymphoma	0.986
0.045	0.015	M21	Melanoma	Skin	Melanoma	0.985
0.028	0.011	JY	EBV-positive lymphoblastoid B cell line	Blood	Normal	1.000

Table S10. CLC-Pred data of the azaheterocyclic derivative **3j**.

Pa	Pi	Cell-line	Description	Tissue/Organ	Type	IAP*
0.608	0.005	SW48	Colorectal Adenocarcinoma	Colon	Adenocarcinoma	0.806
0.527	0.076	MCF7	Breast carcinoma	Breast	Carcinoma	0.836
0.489	0.067	SK-LU-1	Adenocarcinoma	Lung	Carcinoma	0.810
0.444	0.028	UMUC3	Bladder Carcinoma	Urinary tract	Carcinoma	0.810
0.432	0.017	HEL299	Fibroblasts	Lung	Normal	0.899
0.424	0.071	HCC1937	Breast Carcinoma	Breast	Carcinoma	0.806
0.415	0.025	SK-MES-1	Squamous cell lung carcinoma	Lung	Carcinoma	0.834
0.392	0.122	DU-4475	Breast Carcinoma	Breast	Carcinoma	0.802
0.383	0.038	NCI-H292	Mucoepidermoid Pulmonary Carcinoma	Lung	Carcinoma	0.845
0.381	0.095	NCI-H358	Bronchioalveolar Carcinoma	Lung; Bronchiole	Carcinoma	0.805
0.376	0.090	SNU-5	Gastric Carcinoma	Stomach	Carcinoma	0.828
0.371	0.036	RD	Rhabdomyosarcoma	Muscle	Normal	0.828
0.369	0.215	TMD8	Diffuse large B-cell lymphoma activated B-cell type	Lymphocytes	Lymphoma	0.811
0.363	0.040	HuP-T3	Pancreatic adenocarcinoma	Pancreas	Adenocarcinoma	0.868
0.362	0.039	SR	Adult immunoblastic lymphoma	Haematopoietic and lymphoid tissue	Lymphoma	0.877
0.362	0.071	COR-L23	Lung large cell carcinoma	Lung	Carcinoma	0.834
0.356	0.004	SW1116	Colorectal Adenocarcinoma	Colon	Adenocarcinoma	0.864
0.351	0.106	OE33	Barrett adenocarcinoma	Esophagus	Adenocarcinoma	0.805
0.350	0.110	A172	Glioblastoma	Brain	Glioblastoma	0.807
0.345	0.116	CAL-51	Breast carcinoma	Breast	Carcinoma	0.824
0.343	0.151	SK-MEL-1	Metastatic melanoma	Skin	Melanoma	0.808
0.340	0.064	HCC1806	Acantholytic Squamous Cell Carcinoma	Breast	Carcinoma	0.824
0.339	0.025	MSTO-211H	Biphasic Mesothelioma	Lung	Mesothelioma	0.878
0.338	0.065	SK-OV-3	Ovarian carcinoma	Ovarium	Carcinoma	0.896
0.338	0.092	EOL1	Chronic eosinophilic leukemia, not otherwise specified	Blood	Leukemia	0.861
0.334	0.039	PA-1	Ovarian carcinoma	Ovarium	Carcinoma	0.854
0.334	0.103	KYSE-520	Esophageal squamous cell carcinoma	Esophagus	Carcinoma	0.858
0.332	0.047	NCI-H520	Squamous Cell Carcinoma	Lung	Carcinoma	0.837
0.332	0.050	RPMI-7951	Malignant Melanoma	Skin	Melanoma	0.854
0.317	0.129	RS4-11	Adult B acute lymphoblastic leukemia	Bone Marrow	Leukemia	0.835
0.315	0.057	NCI-H727	Carcinoid	Lung; Bronchus	Carcinoma	0.828
0.313	0.279	A2780cisR	Cisplatin-resistant ovarian carcinoma	Ovarium	Carcinoma	0.838

0.312	0.094	HCC1954	Breast Carcinoma	Breast	Carcinoma	0.829
0.305	0.239	NCI-H441	Papillary adenocarcinoma	Lung	Adenocarcinoma	0.813
0.301	0.079	OVCAR-5	Ovarian adenocarcinoma	Ovarium	Adenocarcinoma	0.866
0.294	0.130	NCI-H661	Lung carcinoma	Lung	Carcinoma	0.831
0.293	0.049	AGS	Gastric adenocarcinoma	Stomach	Adenocarcinoma	0.875
0.292	0.057	NCI-H1581	Non Small Cell Lung Cancer	Lung	Carcinoma	0.871
0.291	0.217	HT1197	Carcinoma	Urinary bladder	Carcinoma	0.815
0.289	0.212	Kasumi-1	Acute myeloblastic leukemia	Blood	Leukemia	0.816
0.288	0.177	DMS-114	Lung carcinoma	Lung	Carcinoma	0.834
0.278	0.084	CAL-27	Squamous Cell Carcinoma	Tongue	Carcinoma	0.854
0.276	0.016	CFPAC-1	Pancreatic carcinoma	Pancreas	Carcinoma	0.905
0.270	0.117	HOS	Osteosarcoma	Bone	Sarcoma	0.863
0.270	0.164	EKVX	Non-small cell lung carcinoma	Lung	Carcinoma	0.841
0.269	0.115	H9	T-lymphoid	Haematopoietic and lymphoid tissue	Leukemia	0.872
0.264	0.167	NCI-H1650	Bronchoalveolar carcinoma	Lung	Adenocarcinoma	0.839
0.263	0.155	8505C	Thyroid gland undifferentiated (anaplastic) carcinoma	Thyroid	Carcinoma	0.832
0.258	0.010	T98G	Glioblastoma	Brain	Carcinoma	0.912
0.253	0.190	PLC-PRF-5	Adult hepatocellular carcinoma	Liver	Carcinoma	0.827
0.250	0.108	SW-620	Colon adenocarcinoma	Colon	Adenocarcinoma	0.887
0.250	0.148	Hs-578T	Invasive ductal breast carcinoma	Breast	Carcinoma	0.869
0.238	0.165	OVCAR-4	Ovarian adenocarcinoma	Ovarium	Adenocarcinoma	0.859
0.237	0.105	NCI-H460	Non-small cell lung carcinoma	Lung	Carcinoma	0.906
0.236	0.144	A2058	Melanoma	Skin	Melanoma	0.859
0.232	0.173	TK-10	Renal carcinoma	Kidney	Carcinoma	0.854
0.227	0.124	A-427	Lung carcinoma	Lung	Carcinoma	0.872
0.225	0.162	U-266	Plasma cell myeloma	Blood	Myeloma	0.870
0.222	0.108	RKO	Colon carcinoma	Colon	Carcinoma	0.879
0.221	0.044	HT-1080	Fibrosarcoma	Soft tissue	Sarcoma	0.918
0.218	0.203	UACC-257	Melanoma	Skin	Melanoma	0.861
0.213	0.179	RXF393	Renal cell carcinoma	Kidney	Carcinoma	0.873
0.206	0.170	HCC2998	Colon adenocarcinoma	Colon	Adenocarcinoma	0.872
0.203	0.143	U2OS	Osteosarcoma	Bone	Sarcoma	0.885
0.201	0.123	CCRF-CEM	Childhood T acute lymphoblastic leukemia	Blood	Leukemia	0.913
0.200	0.121	UACC-62	Melanoma	Skin	Melanoma	0.904
0.199	0.167	CAPAN-1	Pancreas Adenocarcinoma	Pancreas	Adenocarcinoma	0.889
0.199	0.174	KM12	Colon adenocarcinoma	Colon	Adenocarcinoma	0.875
0.197	0.166	MOLT-4	Acute T-lymphoblastic leukemia	Blood	Leukemia	0.885
0.196	0.012	NCI-H2228	Non-small cell lung cancer	Lung	Adenocarcinoma	0.920
0.195	0.193	SNB-75	Glioblastoma	Nervous system	Glioblastoma	0.877

0.193	0.016	IMR-32	Neuroblastoma	Nervous system	Neuroblastoma	0.951
0.191	0.177	CAKI-1	Kidney carcinoma	Kidney	Carcinoma	0.880
0.190	0.072	SW480	Colon adenocarcinoma	Colon	Adenocarcinoma	0.932
0.189	0.101	SK-HEP1	Hepatocellular carcinoma	Liver	Carcinoma	0.912
0.185	0.157	MRC5	Embryonic lung fibroblast	Lung	Normal	0.916
0.182	0.054	Mahlavu	Hepatocellular carcinoma	Liver	Carcinoma	0.937
0.181	0.155	HOP-62	Non-small cell lung carcinoma	Lung	Carcinoma	0.898
0.177	0.170	HOP-18	Non-small cell lung carcinoma	Lung	Carcinoma	0.933
0.176	0.167	SN12C	Renal carcinoma	Kidney	Carcinoma	0.896
0.155	0.036	SJSA-1	Osteosarcoma	Bone	Sarcoma	0.940
0.148	0.013	KARPAS-299	Anaplastic large cell lymphoma	Haematopoietic and lymphoid tissue	Leukemia	0.939
0.140	0.089	SKM-1	Adult acute myeloid leukemia	Blood	Leukemia	0.917
0.134	0.112	A-431	Epidermoid carcinoma	Skin	Carcinoma	0.937
0.132	0.086	DLD-1	Colon adenocarcinoma	Colon	Adenocarcinoma	0.949
0.121	0.087	RWPE-1	Prostatic epithelial cell line	Prostate	Normal	0.922
0.100	0.045	HA22T	Hepatocellular carcinoma	Liver	Carcinoma	0.946
0.088	0.062	ZR-75-1	Breast carcinoma	Breast	Carcinoma	0.965
0.068	0.058	KBM5	Chronic myelogenous leukemia, BCR-ABL1 positive	Blood	Leukemia	0.931
0.067	0.028	NAMALVA	EBV-related Burkitt lymphoma	Blood	Lymphoma	0.986
0.045	0.015	M21	Melanoma	Skin	Melanoma	0.985
0.027	0.013	JY	EBV-positive lymphoblastoid B cell line	Blood	Normal	1.000
0.011	0.010	QG-56	Squamous cell lung carcinoma	Lung	Carcinoma	1.000

Table S11. CLC-Pred data of the azaheterocyclic derivative **3k**.

Pa	Pi	Cell-line	Description	Tissue/Organ	Type	IAP*
0.569	0.007	SW48	Colorectal Adenocarcinoma	Colon	Adenocarcinoma	0.806
0.520	0.079	MCF7	Breast carcinoma	Breast	Carcinoma	0.836
0.491	0.064	SK-LU-1	Adenocarcinoma	Lung	Carcinoma	0.810
0.454	0.023	UMUC3	Bladder Carcinoma	Urinary tract	Carcinoma	0.810
0.446	0.049	HCC1937	Breast Carcinoma	Breast	Carcinoma	0.806
0.437	0.016	HEL299	Fibroblasts	Lung	Normal	0.899
0.414	0.026	SK-MES-1	Squamous cell lung carcinoma	Lung	Carcinoma	0.834
0.387	0.128	DU-4475	Breast Carcinoma	Breast	Carcinoma	0.802
0.372	0.036	RD	Rhabdomyosarcoma	Muscle	Normal	0.828
0.372	0.045	NCI-H292	Mucoepidermoid Pulmonary Carcinoma	Lung	Carcinoma	0.845
0.372	0.062	A172	Glioblastoma	Brain	Glioblastoma	0.807
0.371	0.095	SNU-5	Gastric Carcinoma	Stomach	Carcinoma	0.828

0.367	0.112	NCI-H358	Bronchioalveolar Carcinoma	Lung; Bronchiole	Carcinoma	0.805
0.362	0.041	HuP-T3	Pancreatic adenocarcinoma	Pancreas	Adenocarcinoma	0.868
0.360	0.074	COR-L23	Lung large cell carcinoma	Lung	Carcinoma	0.834
0.346	0.147	SK-MEL-1	Metastatic melanoma	Skin	Melanoma	0.808
0.341	0.121	OE33	Barrett adenocarcinoma	Esophagus	Adenocarcinoma	0.805
0.340	0.127	CAL-51	Breast carcinoma	Breast	Carcinoma	0.824
0.339	0.005	SW1116	Colorectal Adenocarcinoma	Colon	Adenocarcinoma	0.864
0.339	0.065	HCC1806	Acantholytic Squamous Cell Carcinoma	Breast	Carcinoma	0.824
0.338	0.092	EOL1	Chronic eosinophilic leukemia, not otherwise specified	Blood	Leukemia	0.861
0.337	0.098	KYSE-520	Esophageal squamous cell carcinoma	Esophagus	Carcinoma	0.858
0.336	0.046	SR	Adult immunoblastic lymphoma	Haematopoietic and lymphoid tissue	Lymphoma	0.877
0.334	0.236	OCI-AML2	Adult acute myeloid leukemia	Blood	Leukemia	0.803
0.332	0.047	NCI-H520	Squamous Cell Carcinoma	Lung	Carcinoma	0.837
0.328	0.279	TMD8	Diffuse large B-cell lymphoma activated B-cell type	Lymphocytes	Lymphoma	0.811
0.325	0.036	MSTO-211H	Biphasic Mesothelioma	Lung	Mesothelioma	0.878
0.324	0.046	NCI-H727	Carcinoid	Lung; Bronchus	Carcinoma	0.828
0.324	0.058	RPMI-7951	Malignant Melanoma	Skin	Melanoma	0.854
0.323	0.050	PA-1	Ovarian carcinoma	Ovarium	Carcinoma	0.854
0.323	0.207	NCI-H441	Papillary adenocarcinoma	Lung	Adenocarcinoma	0.813
0.310	0.141	RS4-11	Adult B acute lymphoblastic leukemia	Bone Marrow	Leukemia	0.835
0.307	0.107	HCC1954	Breast Carcinoma	Breast	Carcinoma	0.829
0.302	0.042	NCI-H1581	Non Small Cell Lung Cancer	Lung	Carcinoma	0.871
0.302	0.194	HT1197	Carcinoma	Urinary bladder	Carcinoma	0.815
0.293	0.084	OVCAR-5	Ovarian adenocarcinoma	Ovarium	Adenocarcinoma	0.866
0.293	0.167	DMS-114	Lung carcinoma	Lung	Carcinoma	0.834
0.283	0.161	NCI-H661	Lung carcinoma	Lung	Carcinoma	0.831
0.280	0.224	Kasumi-1	Acute myeloblastic leukemia	Blood	Leukemia	0.816
0.277	0.069	AGS	Gastric adenocarcinoma	Stomach	Adenocarcinoma	0.875
0.277	0.085	CAL-27	Squamous Cell Carcinoma	Tongue	Carcinoma	0.854
0.276	0.106	H9	T-lymphoid	Haematopoietic and lymphoid tissue	Leukemia	0.872
0.272	0.252	SNU-475	Hepatocellular carcinoma	Liver	Carcinoma	0.819
0.268	0.100	SK-OV-3	Ovarian carcinoma	Ovarium	Carcinoma	0.896
0.264	0.128	HOS	Osteosarcoma	Bone	Sarcoma	0.863
0.264	0.154	8505C	Thyroid gland undifferentiated (anaplastic) carcinoma	Thyroid	Carcinoma	0.832
0.262	0.152	PLC-PRF-5	Adult hepatocellular carcinoma	Liver	Carcinoma	0.827

0.255	0.119	A2058	Melanoma	Skin	Melanoma	0.859
0.253	0.202	EKVX	Non-small cell lung carcinoma	Lung	Carcinoma	0.841
0.249	0.084	A-427	Lung carcinoma	Lung	Carcinoma	0.872
0.249	0.150	Hs-578T	Invasive ductal breast carcinoma	Breast	Carcinoma	0.869
0.248	0.168	RL	Non-Hodgkin's Lymphoma	Ascites	Lymphoma	0.833
0.244	0.023	CFPAC-1	Pancreatic carcinoma	Pancreas	Carcinoma	0.905
0.244	0.232	NCI-H1650	Bronchoalveolar carcinoma	Lung	Adenocarcinoma	0.839
0.236	0.015	T98G	Glioblastoma	Brain	Carcinoma	0.912
0.227	0.158	U-266	Plasma cell myeloma	Blood	Myeloma	0.870
0.224	0.192	OVCAR-4	Ovarian adenocarcinoma	Ovarium	Adenocarcinoma	0.859
0.222	0.194	TK-10	Renal carcinoma	Kidney	Carcinoma	0.854
0.220	0.145	SW-620	Colon adenocarcinoma	Colon	Adenocarcinoma	0.887
0.219	0.110	CCRF-CEM	Childhood T acute lymphoblastic leukemia	Blood	Leukemia	0.913
0.217	0.060	SW480	Colon adenocarcinoma	Colon	Adenocarcinoma	0.932
0.215	0.123	NCI-H460	Non-small cell lung carcinoma	Lung	Carcinoma	0.906
0.214	0.210	UACC-257	Melanoma	Skin	Melanoma	0.861
0.212	0.134	RKO	Colon carcinoma	Colon	Carcinoma	0.879
0.211	0.192	HL-60	Promyeloblast leukemia	Haematopoietic and lymphoid tissue	Leukemia	0.881
0.209	0.052	HT-1080	Fibrosarcoma	Soft tissue	Sarcoma	0.918
0.207	0.193	RXF393	Renal cell carcinoma	Kidney	Carcinoma	0.873
0.196	0.013	NCI-H2228	Non-small cell lung cancer	Lung	Adenocarcinoma	0.920
0.194	0.194	SNB-75	Glioblastoma	Nervous system	Glioblastoma	0.877
0.190	0.017	IMR-32	Neuroblastoma	Nervous system	Neuroblastoma	0.951
0.188	0.183	MOLT-4	Acute T-lymphoblastic leukemia	Blood	Leukemia	0.885
0.187	0.104	SK-HEP1	Hepatocellular carcinoma	Liver	Carcinoma	0.912
0.179	0.158	HOP-62	Non-small cell lung carcinoma	Lung	Carcinoma	0.898
0.179	0.167	HOP-18	Non-small cell lung carcinoma	Lung	Carcinoma	0.933
0.174	0.169	SN12C	Renal carcinoma	Kidney	Carcinoma	0.896
0.173	0.155	UACC-62	Melanoma	Skin	Melanoma	0.904
0.159	0.081	A-431	Epidermoid carcinoma	Skin	Carcinoma	0.937
0.155	0.099	T-24	Bladder carcinoma	Urinary tract	Carcinoma	0.925
0.148	0.013	KARPAS-299	Anaplastic large cell lymphoma	Haematopoietic and lymphoid tissue	Leukemia	0.939
0.147	0.098	Mahlavu	Hepatocellular carcinoma	Liver	Carcinoma	0.937
0.137	0.125	U-87MG	Glioblastoma	Brain	Blastoma	0.921
0.133	0.132	SKM-1	Adult acute myeloid leukemia	Blood	Leukemia	0.917
0.116	0.043	PC-14	Lung adenocarcinoma	Lung	Carcinoma	0.932
0.115	0.110	DLD-1	Colon adenocarcinoma	Colon	Adenocarcinoma	0.949
0.088	0.069	HA22T	Hepatocellular carcinoma	Liver	Carcinoma	0.946
0.062	0.038	NAMALVA	EBV-related Burkitt lymphoma	Blood	Lymphoma	0.986

0.045	0.015	M21	Melanoma	Skin	Melanoma	0.985
0.026	0.014	JY	EBV-positive lymphoblastoid B cell line	Blood	Normal	1.000

Table S12. CLC-Pred data of the azaheterocyclic derivative **3l**.

Pa	Pi	Cell-line	Description	Tissue/Organ	Type	IAP*
0.589	0.005	SW48	Colorectal Adenocarcinoma	Colon	Adenocarcinoma	0.806
0.511	0.047	SK-LU-1	Adenocarcinoma	Lung	Carcinoma	0.810
0.434	0.013	NCI-H292	Mucoepidermoid Pulmonary Carcinoma	Lung	Carcinoma	0.845
0.423	0.041	UMUC3	Bladder Carcinoma	Urinary tract	Carcinoma	0.810
0.421	0.046	SNU-5	Gastric Carcinoma	Stomach	Carcinoma	0.828
0.416	0.006	CFPAC-1	Pancreatic carcinoma	Pancreas	Carcinoma	0.905
0.416	0.020	RD	Rhabdomyosarcoma	Muscle	Normal	0.828
0.415	0.082	HCC1937	Breast Carcinoma	Breast	Carcinoma	0.806
0.402	0.022	HEL299	Fibroblasts	Lung	Normal	0.899
0.401	0.038	SK-MES-1	Squamous cell lung carcinoma	Lung	Carcinoma	0.834
0.375	0.056	COR-L23	Lung large cell carcinoma	Lung	Carcinoma	0.834
0.373	0.004	SW1116	Colorectal Adenocarcinoma	Colon	Adenocarcinoma	0.864
0.369	0.155	DU-4475	Breast Carcinoma	Breast	Carcinoma	0.802
0.369	0.197	OCI-AML2	Adult acute myeloid leukemia	Blood	Leukemia	0.803
0.363	0.041	HuP-T3	Pancreatic adenocarcinoma	Pancreas	Adenocarcinoma	0.868
0.363	0.059	KYSE-520	Esophageal squamous cell carcinoma	Esophagus	Carcinoma	0.858
0.350	0.024	NCI-H727	Carcinoid	Lung; Bronchus	Carcinoma	0.828
0.349	0.079	EOL1	Chronic eosinophilic leukemia, not otherwise specified	Blood	Leukemia	0.861
0.346	0.121	A172	Glioblastoma	Brain	Glioblastoma	0.807
0.345	0.056	HCC1806	Acantholytic Squamous Cell Carcinoma	Breast	Carcinoma	0.824
0.345	0.177	MCF7	Breast carcinoma	Breast	Carcinoma	0.836
0.343	0.147	NCI-H358	Bronchioalveolar Carcinoma	Lung; Bronchiole	Carcinoma	0.805
0.342	0.153	SK-MEL-1	Metastatic melanoma	Skin	Melanoma	0.808
0.331	0.170	YAPC	Pancreatic carcinoma	Pancreas	Carcinoma	0.817
0.330	0.140	OE33	Barrett adenocarcinoma	Esophagus	Adenocarcinoma	0.805
0.328	0.044	PA-1	Ovarian carcinoma	Ovarium	Carcinoma	0.854
0.326	0.054	SW-620	Colon adenocarcinoma	Colon	Adenocarcinoma	0.887
0.320	0.213	NCI-H441	Papillary adenocarcinoma	Lung	Adenocarcinoma	0.813
0.309	0.283	A2780cisR	Cisplatin-resistant ovarian carcinoma	Ovarium	Carcinoma	0.838
0.307	0.188	Kasumi-1	Acute myeloblastic leukemia	Blood	Leukemia	0.816

0.306	0.146	RS4-11	Adult B acute lymphoblastic leukemia	Bone Marrow	Leukemia	0.835
0.297	0.065	SR	Adult immunoblastic lymphoma	Haematopoietic and lymphoid tissue	Lymphoma	0.877
0.295	0.060	MSTO-211H	Biphasic Mesothelioma	Lung	Mesothelioma	0.878
0.294	0.237	CAL-51	Breast carcinoma	Breast	Carcinoma	0.824
0.292	0.101	RPMI-7951	Malignant Melanoma	Skin	Melanoma	0.854
0.290	0.160	HCC1954	Breast Carcinoma	Breast	Carcinoma	0.829
0.289	0.221	HT1197	Carcinoma	Urinary bladder	Carcinoma	0.815
0.287	0.132	5637	Urothelial bladder carcinoma	Urinary tract	Carcinoma	0.839
0.281	0.100	HOS	Osteosarcoma	Bone	Sarcoma	0.863
0.278	0.083	U-266	Plasma cell myeloma	Blood	Myeloma	0.870
0.277	0.018	KG-1	Acute myelogenous leukemia	Blood	Leukemia	0.901
0.275	0.092	CAL-27	Squamous Cell Carcinoma	Tongue	Carcinoma	0.854
0.272	0.101	A2058	Melanoma	Skin	Melanoma	0.859
0.269	0.159	NCI-H520	Squamous Cell Carcinoma	Lung	Carcinoma	0.837
0.255	0.114	NCI-H1581	Non Small Cell Lung Cancer	Lung	Carcinoma	0.871
0.251	0.190	8505C	Thyroid gland undifferentiated (anaplastic) carcinoma	Thyroid	Carcinoma	0.832
0.249	0.124	OVCAR-5	Ovarian adenocarcinoma	Ovarium	Adenocarcinoma	0.866
0.240	0.159	H9	T-lymphoid	Haematopoietic and lymphoid tissue	Leukemia	0.872
0.238	0.142	AGS	Gastric adenocarcinoma	Stomach	Adenocarcinoma	0.875
0.225	0.209	MKN-74	Gastric tubular adenocarcinoma	Stomach	Adenocarcinoma	0.856
0.215	0.156	A-427	Lung carcinoma	Lung	Carcinoma	0.872
0.213	0.026	T98G	Glioblastoma	Brain	Carcinoma	0.912
0.210	0.194	MDA-MB-361	Breast adenocarcinoma	Breast	Adenocarcinoma	0.872
0.200	0.175	RKO	Colon carcinoma	Colon	Carcinoma	0.879
0.199	0.163	G-361	Melanoma	Skin	Melanoma	0.890
0.196	0.013	NCI-H2228	Non-small cell lung cancer	Lung	Adenocarcinoma	0.920
0.195	0.163	SK-OV-3	Ovarian carcinoma	Ovarium	Carcinoma	0.896
0.187	0.091	ASPC1	Pancreatic ductal adenocarcinoma	Pancreas	Adenocarcinoma	0.901
0.181	0.150	MOLM-14	Adult acute myeloid leukemia	Blood	Leukemia	0.888
0.175	0.162	NCI-H460	Non-small cell lung carcinoma	Lung	Carcinoma	0.906
0.172	0.071	A-431	Epidermoid carcinoma	Skin	Carcinoma	0.937
0.167	0.006	KARPAS-299	Anaplastic large cell lymphoma	Haematopoietic and lymphoid tissue	Leukemia	0.939
0.164	0.088	SW480	Colon adenocarcinoma	Colon	Adenocarcinoma	0.932
0.163	0.149	SK-HEP1	Hepatocellular carcinoma	Liver	Carcinoma	0.912
0.162	0.019	SJSA-1	Osteosarcoma	Bone	Sarcoma	0.940
0.157	0.141	SUM149PT	Breast inflammatory carcinoma	Breast	Carcinoma	0.983

0.154	0.032	IMR-32	Neuroblastoma	Nervous system	Neuroblastoma	0.951
0.150	0.070	DLD-1	Colon adenocarcinoma	Colon	Adenocarcinoma	0.949
0.149	0.053	SKM-1	Adult acute myeloid leukemia	Blood	Leukemia	0.917
0.147	0.144	HT-1080	Fibrosarcoma	Soft tissue	Sarcoma	0.918
0.145	0.066	Jurkat	Acute leukemic T-cells	Blood	Leukemia	0.946
0.133	0.089	MV4-11	Myeloid leukemia	Haematopoietic and lymphoid tissue	Leukemia	0.947
0.115	0.113	RWPE-1	Prostatic epithelial cell line	Prostate	Normal	0.922
0.110	0.106	SK-BR-3	Breast adenocarcinoma	Breast	Adenocarcinoma	0.950
0.103	0.095	WI-38	Embryonic lung fibroblast	Lung	Normal	0.943
0.102	0.098	HFF	Foreskin fibroblast	Skin	Normal	0.955
0.078	0.077	BEAS-2B	Epithelial cells	bronchial epithelium	Normal	1.000
0.042	0.020	M21	Melanoma	Skin	Melanoma	0.985
0.040	0.005	JY	EBV-positive lymphoblastoid B cell line	Blood	Normal	1.000
0.021	0.005	QG-56	Squamous cell lung carcinoma	Lung	Carcinoma	1.000

Table S13. CLC-Pred data of the azahetetrocyclic derivative **3m**.

Pa	Pi	Cell-line	Description	Tissue/Organ	Type	IAP*
0.545	0.010	SW48	Colorectal Adenocarcinoma	Colon	Adenocarcinoma	0.806
0.458	0.100	SK-LU-1	Adenocarcinoma	Lung	Carcinoma	0.810
0.453	0.024	UMUC3	Bladder Carcinoma	Urinary tract	Carcinoma	0.810
0.425	0.019	SK-MES-1	Squamous cell lung carcinoma	Lung	Carcinoma	0.834
0.415	0.020	HEL299	Fibroblasts	Lung	Normal	0.899
0.410	0.018	HuP-T3	Pancreatic adenocarcinoma	Pancreas	Adenocarcinoma	0.868
0.406	0.106	DU-4475	Breast Carcinoma	Breast	Carcinoma	0.802
0.360	0.043	RD	Rhabdomyosarcoma	Muscle	Normal	0.828
0.355	0.045	HCC1806	Acantholytic Squamous Cell Carcinoma	Breast	Carcinoma	0.824
0.354	0.004	SW1116	Colorectal Adenocarcinoma	Colon	Adenocarcinoma	0.864
0.348	0.176	HCC1937	Breast Carcinoma	Breast	Carcinoma	0.806
0.340	0.256	A2780cisR	Cisplatin-resistant ovarian carcinoma	Ovarium	Carcinoma	0.838
0.332	0.146	SNU-5	Gastric Carcinoma	Stomach	Carcinoma	0.828
0.329	0.157	Kasumi-1	Acute myeloblastic leukemia	Blood	Leukemia	0.816
0.328	0.104	EOL1	Chronic eosinophilic leukemia, not otherwise specified	Blood	Leukemia	0.861
0.327	0.097	NCI-H292	Mucoepidermoid Pulmonary Carcinoma	Lung	Carcinoma	0.845
0.324	0.051	HOS	Osteosarcoma	Bone	Sarcoma	0.863
0.320	0.071	HCC1954	Breast Carcinoma	Breast	Carcinoma	0.829

0.319	0.252	OCI-AML2	Adult acute myeloid leukemia	Blood	Leukemia	0.803
0.318	0.149	COR-L23	Lung large cell carcinoma	Lung	Carcinoma	0.834
0.317	0.198	A172	Glioblastoma	Brain	Glioblastoma	0.807
0.314	0.137	KYSE-520	Esophageal squamous cell carcinoma	Esophagus	Carcinoma	0.858
0.312	0.026	U2OS	Osteosarcoma	Bone	Sarcoma	0.885
0.311	0.065	PA-1	Ovarian carcinoma	Ovarium	Carcinoma	0.854
0.306	0.082	RPMI-7951	Malignant Melanoma	Skin	Melanoma	0.854
0.300	0.090	NCI-H520	Squamous Cell Carcinoma	Lung	Carcinoma	0.837
0.299	0.189	OE33	Barrett adenocarcinoma	Esophagus	Adenocarcinoma	0.805
0.298	0.044	AGS	Gastric adenocarcinoma	Stomach	Adenocarcinoma	0.875
0.287	0.149	NCI-H661	Lung carcinoma	Lung	Carcinoma	0.831
0.287	0.270	SK-MEL-1	Metastatic melanoma	Skin	Melanoma	0.808
0.282	0.125	NCI-H727	Carcinoid	Lung; Bronchus	Carcinoma	0.828
0.281	0.270	NCI-H358	Bronchioalveolar Carcinoma	Lung; Bronchiole	Carcinoma	0.805
0.274	0.256	HT1197	Carcinoma	Urinary bladder	Carcinoma	0.815
0.261	0.133	CAL-27	Squamous Cell Carcinoma	Tongue	Carcinoma	0.854
0.258	0.190	EKVX	Non-small cell lung carcinoma	Lung	Carcinoma	0.841
0.257	0.133	H9	T-lymphoid	Haematopoietic and lymphoid tissue	Leukemia	0.872
0.256	0.101	MSTO-211H	Biphasic Mesothelioma	Lung	Mesothelioma	0.878
0.255	0.109	U-266	Plasma cell myeloma	Blood	Myeloma	0.870
0.251	0.076	G-361	Melanoma	Skin	Melanoma	0.890
0.247	0.022	CFPAC-1	Pancreatic carcinoma	Pancreas	Carcinoma	0.905
0.247	0.148	TK-10	Renal carcinoma	Kidney	Carcinoma	0.854
0.245	0.130	A2058	Melanoma	Skin	Melanoma	0.859
0.243	0.134	NCI-H1581	Non Small Cell Lung Cancer	Lung	Carcinoma	0.871
0.241	0.224	8505C	Thyroid gland undifferentiated (anaplastic) carcinoma	Thyroid	Carcinoma	0.832
0.240	0.138	OVCAR-5	Ovarian adenocarcinoma	Ovarium	Adenocarcinoma	0.866
0.234	0.007	IMR-32	Neuroblastoma	Nervous system	Neuroblastoma	0.951
0.198	0.138	NCI-H460	Non-small cell lung carcinoma	Lung	Carcinoma	0.906
0.198	0.142	MRC5	Embryonic lung fibroblast	Lung	Normal	0.916
0.197	0.177	SW1990	Pancreatic adenocarcinoma	Pancreas	Adenocarcinoma	0.876
0.195	0.041	T98G	Glioblastoma	Brain	Carcinoma	0.912
0.191	0.020	NCI-H2228	Non-small cell lung cancer	Lung	Adenocarcinoma	0.920
0.189	0.100	SK-HEP1	Hepatocellular carcinoma	Liver	Carcinoma	0.912
0.189	0.169	SK-OV-3	Ovarian carcinoma	Ovarium	Carcinoma	0.896
0.179	0.056	DLD-1	Colon adenocarcinoma	Colon	Adenocarcinoma	0.949
0.177	0.081	HT-1080	Fibrosarcoma	Soft tissue	Sarcoma	0.918
0.143	0.077	SKM-1	Adult acute myeloid leukemia	Blood	Leukemia	0.917

0.139	0.021	KARPAS-299	Anaplastic large cell lymphoma	Haematopoietic and lymphoid tissue	Leukemia	0.939
0.134	0.111	A-431	Epidermoid carcinoma	Skin	Carcinoma	0.937
0.116	0.052	Daoy	Desmoplastic Cerebellar Medulloblastoma	Brain	Medulloblastoma	0.908
0.104	0.093	WI-38	Embryonic lung fibroblast	Lung	Normal	0.943
0.043	0.017	M21	Melanoma	Skin	Melanoma	0.985
0.031	0.009	JY	EBV-positive lymphoblastoid B cell line	Blood	Normal	1.000

Table S14. CLC-Pred data of the azaheterocyclic derivative **3n**.

Pa	Pi	Cell-line	Description	Tissue/Organ	Type	IAP*
0.548	0.009	SW48	Colorectal Adenocarcinoma	Colon	Adenocarcinoma	0.806
0.528	0.076	MCF7	Breast carcinoma	Breast	Carcinoma	0.836
0.517	0.042	SK-LU-1	Adenocarcinoma	Lung	Carcinoma	0.810
0.436	0.032	UMUC3	Bladder Carcinoma	Urinary tract	Carcinoma	0.810
0.429	0.027	SR	Adult immunoblastic lymphoma	Haematopoietic and lymphoid tissue	Lymphoma	0.877
0.403	0.026	A172	Glioblastoma	Brain	Glioblastoma	0.807
0.400	0.053	DMS-114	Lung carcinoma	Lung	Carcinoma	0.834
0.397	0.041	SK-MES-1	Squamous cell lung carcinoma	Lung	Carcinoma	0.834
0.393	0.049	M19-MEL	Melanoma	Skin	Melanoma	0.925
0.393	0.121	DU-4475	Breast Carcinoma	Breast	Carcinoma	0.802
0.392	0.024	HEL299	Fibroblasts	Lung	Normal	0.899
0.392	0.027	RD	Rhabdomyosarcoma	Muscle	Normal	0.828
0.388	0.034	NCI-H292	Mucoepidermoid Pulmonary Carcinoma	Lung	Carcinoma	0.845
0.375	0.034	HuP-T3	Pancreatic adenocarcinoma	Pancreas	Adenocarcinoma	0.868
0.366	0.026	NCI-H520	Squamous Cell Carcinoma	Lung	Carcinoma	0.837
0.351	0.005	SW1116	Colorectal Adenocarcinoma	Colon	Adenocarcinoma	0.864
0.348	0.111	OE33	Barrett adenocarcinoma	Esophagus	Adenocarcinoma	0.805
0.348	0.122	SNU-5	Gastric Carcinoma	Stomach	Carcinoma	0.828
0.344	0.057	HCC1806	Acantholytic Squamous Cell Carcinoma	Breast	Carcinoma	0.824
0.341	0.047	H9	T-lymphoid	Haematopoietic and lymphoid tissue	Leukemia	0.872
0.341	0.137	A549	Lung carcinoma	Lung	Carcinoma	0.856
0.340	0.061	OVCAR-5	Ovarian adenocarcinoma	Ovarium	Adenocarcinoma	0.866
0.340	0.108	COR-L23	Lung large cell carcinoma	Lung	Carcinoma	0.834
0.334	0.138	CAL-51	Breast carcinoma	Breast	Carcinoma	0.824
0.334	0.167	SK-MEL-1	Metastatic melanoma	Skin	Melanoma	0.808

0.330	0.102	EOL1	Chronic eosinophilic leukemia, not otherwise specified	Blood	Leukemia	0.861
0.321	0.186	NCI-H358	Bronchioalveolar Carcinoma	Lung; Bronchiole	Carcinoma	0.805
0.320	0.058	HOP-62	Non-small cell lung carcinoma	Lung	Carcinoma	0.898
0.318	0.055	PA-1	Ovarian carcinoma	Ovarium	Carcinoma	0.854
0.317	0.058	SN12C	Renal carcinoma	Kidney	Carcinoma	0.896
0.309	0.078	RPMI-7951	Malignant Melanoma	Skin	Melanoma	0.854
0.308	0.080	RXF393	Renal cell carcinoma	Kidney	Carcinoma	0.873
0.307	0.148	KYSE-520	Esophageal squamous cell carcinoma	Esophagus	Carcinoma	0.858
0.304	0.061	Hs-578T	Invasive ductal breast carcinoma	Breast	Carcinoma	0.869
0.303	0.069	SF-295	Glioblastoma	Brain	Glioblastoma	0.884
0.295	0.257	HCC1937	Breast Carcinoma	Breast	Carcinoma	0.806
0.291	0.077	HCC2998	Colon adenocarcinoma	Colon	Adenocarcinoma	0.872
0.289	0.128	EKVX	Non-small cell lung carcinoma	Lung	Carcinoma	0.841
0.288	0.085	NCI-H226	Non-small cell lung carcinoma	Lung	Carcinoma	0.880
0.286	0.097	MKN-74	Gastric tubular adenocarcinoma	Stomach	Adenocarcinoma	0.856
0.284	0.055	UACC-62	Melanoma	Skin	Melanoma	0.904
0.284	0.091	A2058	Melanoma	Skin	Melanoma	0.859
0.282	0.014	CFPAC-1	Pancreatic carcinoma	Pancreas	Carcinoma	0.905
0.279	0.090	KM12	Colon adenocarcinoma	Colon	Adenocarcinoma	0.875
0.278	0.114	OVCAR-4	Ovarian adenocarcinoma	Ovarium	Adenocarcinoma	0.859
0.277	0.077	MSTO-211H	Biphasic Mesothelioma	Lung	Mesothelioma	0.878
0.272	0.117	TK-10	Renal carcinoma	Kidney	Carcinoma	0.854
0.264	0.009	T98G	Glioblastoma	Brain	Carcinoma	0.912
0.264	0.119	UACC-257	Melanoma	Skin	Melanoma	0.861
0.262	0.176	NCI-H1650	Bronchoalveolar carcinoma	Lung	Adenocarcinoma	0.839
0.261	0.230	NCI-H661	Lung carcinoma	Lung	Carcinoma	0.831
0.261	0.250	Kasumi-1	Acute myeloblastic leukemia	Blood	Leukemia	0.816
0.259	0.084	OVCAR-3	Ovarian adenocarcinoma	Ovarium	Adenocarcinoma	0.907
0.258	0.204	NCI-H727	Carcinoid	Lung; Bronchus	Carcinoma	0.828
0.257	0.108	SK-OV-3	Ovarian carcinoma	Ovarium	Carcinoma	0.896
0.256	0.149	CAL-27	Squamous Cell Carcinoma	Tongue	Carcinoma	0.854
0.253	0.083	HOP-18	Non-small cell lung carcinoma	Lung	Carcinoma	0.933
0.247	0.238	5637	Urothelial bladder carcinoma	Urinary tract	Carcinoma	0.839
0.246	0.206	8505C	Thyroid gland undifferentiated (anaplastic) carcinoma	Thyroid	Carcinoma	0.832
0.245	0.113	SW-620	Colon adenocarcinoma	Colon	Adenocarcinoma	0.887
0.242	0.164	HL-60	Promyeloblast leukemia	Haematopoietic and lymphoid tissue	Leukemia	0.881
0.240	0.139	AGS	Gastric adenocarcinoma	Stomach	Adenocarcinoma	0.875
0.240	0.176	HOS	Osteosarcoma	Bone	Sarcoma	0.863

0.240	0.236	C8166	Leukemic T-cells	Blood	Leukemia	0.839
0.237	0.006	IMR-32	Neuroblastoma	Nervous system	Neuroblastoma	0.951
0.235	0.111	SF-539	Glioblastoma	Brain	Glioblastoma	0.888
0.233	0.130	SNB-75	Glioblastoma	Nervous system	Glioblastoma	0.877
0.232	0.122	RPMI-8226	Multiple myeloma	Haematopoietic and lymphoid tissue	Myeloma	0.883
0.217	0.148	DU-145	Prostate carcinoma	Prostate	Carcinoma	0.896
0.216	0.122	M14	Melanoma	Skin	Melanoma	0.894
0.197	0.165	CAKI-1	Kidney carcinoma	Kidney	Carcinoma	0.880
0.196	0.171	SK-MEL-28	Melanoma	Skin	Melanoma	0.879
0.195	0.194	786-0	Renal carcinoma	Kidney	Carcinoma	0.877
0.192	0.135	SK-MEL-5	Melanoma	Skin	Melanoma	0.903
0.191	0.132	CCRF-CEM	Childhood T acute lymphoblastic leukemia	Blood	Leukemia	0.913
0.189	0.023	NCI-H2228	Non-small cell lung cancer	Lung	Adenocarcinoma	0.920
0.189	0.147	COLO-205	Colorectal Adenocarcinoma	Colon	Adenocarcinoma	0.901
0.188	0.183	MOLT-4	Acute T-lymphoblastic leukemia	Blood	Leukemia	0.885
0.183	0.087	BXPC-3	Pancreatic adenocarcinoma	Pancreas	Adenocarcinoma	0.917
0.173	0.081	SW480	Colon adenocarcinoma	Colon	Adenocarcinoma	0.932
0.172	0.089	HT-1080	Fibrosarcoma	Soft tissue	Sarcoma	0.918
0.171	0.165	NCI-H460	Non-small cell lung carcinoma	Lung	Carcinoma	0.906
0.146	0.069	SJSA-1	Osteosarcoma	Bone	Sarcoma	0.940
0.135	0.026	KARPAS-299	Anaplastic large cell lymphoma	Haematopoietic and lymphoid tissue	Leukemia	0.939
0.134	0.055	JeKo-1	Lymphoma	Blood	Lymphoma	0.948
0.122	0.100	DLD-1	Colon adenocarcinoma	Colon	Adenocarcinoma	0.949
0.108	0.084	Ramos	Burkitts lymhoma B-cells	Blood	Leukemia	0.958
0.107	0.040	ZR-75-1	Breast carcinoma	Breast	Carcinoma	0.965
0.086	0.073	HA22T	Hepatocellular carcinoma	Liver	Carcinoma	0.946
0.064	0.010	Daudi	Burkitts Lymphoma	Blood	Lymphoma	0.990
0.047	0.021	K562/Adr	Blast phase chronic myelogenous leukemia, BCR-ABL1 positive	Haematopoietic and lymphoid tissue	Leukemia	0.972
0.042	0.021	M21	Melanoma	Skin	Melanoma	0.985
0.030	0.010	JY	EBV-positive lymphoblastoid B cell line	Blood	Normal	1.000
0.012	0.008	QG-56	Squamous cell lung carcinoma	Lung	Carcinoma	1.000
